# An interconnected data infrastructure to support large-scale rare disease research

**DOI:** 10.1093/gigascience/giae058

**Published:** 2024-09-20

**Authors:** Lennart F Johansson, Steve Laurie, Dylan Spalding, Spencer Gibson, David Ruvolo, Coline Thomas, Davide Piscia, Fernanda de Andrade, Gerieke Been, Marieke Bijlsma, Han Brunner, Sandi Cimerman, Farid Yavari Dizjikan, Kornelia Ellwanger, Marcos Fernandez, Mallory Freeberg, Gert-Jan van de Geijn, Roan Kanninga, Vatsalya Maddi, Mehdi Mehtarizadeh, Pieter Neerincx, Stephan Ossowski, Ana Rath, Dieuwke Roelofs-Prins, Marloes Stok-Benjamins, K Joeri van der Velde, Colin Veal, Gerben van der Vries, Marc Wadsley, Gregory Warren, Birte Zurek, Thomas Keane, Holm Graessner, Sergi Beltran, Morris A Swertz, Anthony J Brookes, Solve-RD consortium, Olaf Riess, Tobias B Haack, Holm Graessner, Birte Zurek, Kornelia Ellwanger, Stephan Ossowski, German Demidov, Marc Sturm, Julia M Schulze-Hentrich, Rebecca Schüle, Jishu Xu, Christoph Kessler, Melanie Kellner, Matthis Synofzik, Carlo Wilke, Andreas Traschütz, Ludger Schöls, Holger Hengel, Holger Lerche, Josua Kegele, Peter Heutink, Han Brunner, Hans Scheffer, Nicoline Hoogerbrugge, Alexander Hoischen, Peter A C ’t Hoen, Lisenka E L M Vissers, Christian Gilissen, Wouter Steyaert, Karolis Sablauskas, Richarda M de Voer, Erik-Jan Kamsteeg, Bart van de Warrenburg, Nienke van Os, Iris te Paske, Erik Janssen, Elke de Boer, Marloes Steehouwer, Burcu Yaldiz, Tjitske Kleefstra, Anthony J Brookes, Colin Veal, Spencer Gibson, Vatsalya Maddi, Mehdi Mehtarizadeh, Umar Riaz, Greg Warren, Farid Yavari Dizjikan, Thomas Shorter, Ana Töpf, Volker Straub, Chiara Marini Bettolo, Jordi Diaz Manera, Sophie Hambleton, Karin Engelhardt, Jill Clayton-Smith, Siddharth Banka, Elizabeth Alexander, Adam Jackson, Laurence Faivre, Christel Thauvin, Antonio Vitobello, Anne-Sophie Denommé-Pichon, Yannis Duffourd, Ange-Line Bruel, Christine Peyron, Aurore Pélissier, Sergi Beltran, Ivo Glynne Gut, Steven Laurie, Davide Piscia, Leslie Matalonga, Anastasios Papakonstantinou, Gemma Bullich, Alberto Corvo, Marcos Fernandez-Callejo, Carles Hernández, Daniel Picó, Ida Paramonov, Hanns Lochmüller, Gulcin Gumus, Virginie Bros-Facer, Ana Rath, Marc Hanauer, David Lagorce, Oscar Hongnat, Maroua Chahdil, Emeline Lebreton, Giovanni Stevanin, Alexandra Durr, Claire-Sophie Davoine, Léna Guillot-Noel, Anna Heinzmann, Giulia Coarelli, Gisèle Bonne, Teresinha Evangelista, Valérie Allamand, Isabelle Nelson, Rabah Ben Yaou, Corinne Metay, Bruno Eymard, Enzo Cohen, Antonio Atalaia, Tanya Stojkovic, Milan Macek, Marek Turnovec, Dana Thomasová, Radka Pourová Kremliková, Vera Franková, Markéta Havlovicová, Petra Lišková, Pavla Doležalová, Helen Parkinson, Thomas Keane, Mallory Freeberg, Coline Thomas, Dylan Spalding, Peter Robinson, Daniel Danis, Glenn Robert, Alessia Costa, Christine Patch, Mike Hanna, Henry Houlden, Mary Reilly, Jana Vandrovcova, Stephanie Efthymiou, Heba Morsy, Elisa Cali, Francesca Magrinelli, Sanjay M Sisodiya, Jonathan Rohrer, Francesco Muntoni, Irina Zaharieva, Anna Sarkozy, Vincent Timmerman, Jonathan Baets, Geert de Vries, Jonathan De Winter, Danique Beijer, Peter de Jonghe, Liedewei Van de Vondel, Willem De Ridder, Sarah Weckhuysen, Vincenzo Nigro, Margherita Mutarelli, Manuela Morleo, Michele Pinelli, Alessandra Varavallo, Sandro Banfi, Annalaura Torella, Francesco Musacchia, Giulio Piluso, Alessandra Ferlini, Rita Selvatici, Francesca Gualandi, Stefania Bigoni, Rachele Rossi, Marcella Neri, Stefan Aretz, Isabel Spier, Anna Katharina Sommer, Sophia Peters, Carla Oliveira, Jose Garcia-Pelaez, Rita Barbosa-Matos, Celina São José, Marta Ferreira, Irene Gullo, Susana Fernandes, Luzia Garrido, Pedro Ferreira, Fátima Carneiro, Morris A Swertz, Lennart Johansson, Joeri K van der Velde, Gerben van der Vries, Pieter B Neerincx, David Ruvolo, Kristin M Abbott, Wilhemina S Kerstjens Frederikse, Eveline Zonneveld-Huijssoon, Dieuwke Roelofs-Prins, Marielle van Gijn, Sebastian Köhler, Alison Metcalfe, Alain Verloes, Séverine Drunat, Delphine Heron, Cyril Mignot, Boris Keren, Jean-Madeleine de Sainte Agathe, Caroline Rooryck, Didier Lacombe, Aurelien Trimouille, Manuel Posada De la Paz, Eva Bermejo Sánchez, Estrella López Martín, Beatriz Martínez Delgado, F Javier Alonso García de la Rosa, Andrea Ciolfi, Bruno Dallapiccola, Simone Pizzi, Francesca Clementina Radio, Marco Tartaglia, Alessandra Renieri, Simone Furini, Chiara Fallerini, Elisa Benetti, Peter Balicza, Maria Judit Molnar, Ales Maver, Borut Peterlin, Alexander Münchau, Katja Lohmann, Rebecca Herzog, Martje Pauly, Alfons Macaya, Ana Cazurro-Gutiérrez, Belén Pérez-Dueñas, Francina Munell, Clara Franco Jarava, Laura Batlle Masó, Anna Marcé-Grau, Roger Colobran, Andrés Nascimento Osorio, Daniel Natera de Benito, Hanns Lochmüller, Rachel Thompson, Kiran Polavarapu, Bodo Grimbacher, David Beeson, Judith Cossins, Peter Hackman, Mridul Johari, Marco Savarese, Bjarne Udd, Rita Horvath, Patrick F Chinnery, Thiloka Ratnaike, Fei Gao, Katherine Schon, Gabriel Capella, Laura Valle, Elke Holinski-Feder, Andreas Laner, Verena Steinke-Lange, Evelin Schröck, Andreas Rump, Ayşe Nazlı Başak, Dimitri Hemelsoet, Bart Dermaut, Nika Schuermans, Bruce Poppe, Hannah Verdin, Davide Mei, Annalisa Vetro, Simona Balestrini, Renzo Guerrini, Kristl Claeys, Gijs W E Santen, Emilia K Bijlsma, Mariette J V Hoffer, Claudia A L Ruivenkamp, Kaan Boztug, Matthias Haimel, Isabelle Maystadt, Isabell Cordts, Marcus Deschauer, Ioannis Zaganas, Evgenia Kokosali, Mathioudakis Lambros, Athanasios Evangeliou, Martha Spilioti, Elisabeth Kapaki, Mara Bourbouli, Pasquale Striano, Federico Zara, Antonella Riva, Michele Iacomino, Paolo Uva, Marcello Scala, Paolo Scudieri, Maria-Roberta Cilio, Evelina Carpancea, Chantal Depondt, Damien Lederer, Yves Sznajer, Sarah Duerinckx, Sandrine Mary, Christel Depienne, Andreas Roos, Patrick May

**Affiliations:** Department of Genetics, University of Groningen, University Medical Center Groningen, HPC CB50, P.O. Box 30001, Groningen, 9700 RB, The Netherlands; Centro Nacional de Análisis Genómico, C/Baldiri Reixac 4, 08028, Barcelona, Spain; Universitat de Barcelona (UB), Gran Via de les Corts Catalanes, 585, L’Eixample, 08007, Barcelona, Spain; European Molecular Biology Laboratory, European Bioinformatics Institute, Wellcome Genome Campus, Hinxton, Cambridge, CV10 1SD, UK; Department of Genetics, Genomics and Cancer Sciences, University of Leicester, University Road, Leicester, Leicester, LE1 7RH, UK; Department of Genetics, University of Groningen, University Medical Center Groningen, HPC CB50, P.O. Box 30001, Groningen, 9700 RB, The Netherlands; European Molecular Biology Laboratory, European Bioinformatics Institute, Wellcome Genome Campus, Hinxton, Cambridge, CV10 1SD, UK; Centro Nacional de Análisis Genómico, C/Baldiri Reixac 4, 08028, Barcelona, Spain; Universitat de Barcelona (UB), Gran Via de les Corts Catalanes, 585, L’Eixample, 08007, Barcelona, Spain; Department of Genetics, University of Groningen, University Medical Center Groningen, HPC CB50, P.O. Box 30001, Groningen, 9700 RB, The Netherlands; Department of Genetics, University of Groningen, University Medical Center Groningen, HPC CB50, P.O. Box 30001, Groningen, 9700 RB, The Netherlands; Department of Genetics, University of Groningen, University Medical Center Groningen, HPC CB50, P.O. Box 30001, Groningen, 9700 RB, The Netherlands; Department of Human Genetics, Radboud University Medical Center, Geert Grooteplein Zuid 10, Nijmegen, 6525 GA, The Netherlands; Donders Institute for Brain, Cognition and Behaviour, Radboud University Medical Center, P.O.Box 9103, Nijmegen, 6500 HD, The Netherlands; Department of Clinical Genetics, Maastricht University Medical Centre, P. Debyelaan 25, Maastricht, 6229 HX, The Netherlands; Department of Genetics, University of Groningen, University Medical Center Groningen, HPC CB50, P.O. Box 30001, Groningen, 9700 RB, The Netherlands; Department of Genetics, Genomics and Cancer Sciences, University of Leicester, University Road, Leicester, Leicester, LE1 7RH, UK; Institute of Medical Genetics and Applied Genomics, University of Tübingen, Calwerstraße 7, Tübingen 72076, Germany; Centro Nacional de Análisis Genómico, C/Baldiri Reixac 4, 08028, Barcelona, Spain; Universitat de Barcelona (UB), Gran Via de les Corts Catalanes, 585, L’Eixample, 08007, Barcelona, Spain; European Molecular Biology Laboratory, European Bioinformatics Institute, Wellcome Genome Campus, Hinxton, Cambridge, CV10 1SD, UK; Department of Genetics, University of Groningen, University Medical Center Groningen, HPC CB50, P.O. Box 30001, Groningen, 9700 RB, The Netherlands; Department of Genetics, University of Groningen, University Medical Center Groningen, HPC CB50, P.O. Box 30001, Groningen, 9700 RB, The Netherlands; Department of Genetics, Genomics and Cancer Sciences, University of Leicester, University Road, Leicester, Leicester, LE1 7RH, UK; Department of Genetics, Genomics and Cancer Sciences, University of Leicester, University Road, Leicester, Leicester, LE1 7RH, UK; Department of Genetics, University of Groningen, University Medical Center Groningen, HPC CB50, P.O. Box 30001, Groningen, 9700 RB, The Netherlands; Institute of Medical Genetics and Applied Genomics, University of Tübingen, Calwerstraße 7, Tübingen 72076, Germany; Institute for Bioinformatics and Medical Informatics (IBMI), University of Tübingen, Geschwister-Scholl-Platz, Tübingen 72074, Germany; INSERM, US‐14 Orphanet, 96 rue Didot, Paris 75014, France; Department of Genetics, University of Groningen, University Medical Center Groningen, HPC CB50, P.O. Box 30001, Groningen, 9700 RB, The Netherlands; Department of Genetics, University of Groningen, University Medical Center Groningen, HPC CB50, P.O. Box 30001, Groningen, 9700 RB, The Netherlands; Department of Genetics, University of Groningen, University Medical Center Groningen, HPC CB50, P.O. Box 30001, Groningen, 9700 RB, The Netherlands; Department of Genetics, Genomics and Cancer Sciences, University of Leicester, University Road, Leicester, Leicester, LE1 7RH, UK; Department of Genetics, University of Groningen, University Medical Center Groningen, HPC CB50, P.O. Box 30001, Groningen, 9700 RB, The Netherlands; Department of Genetics, Genomics and Cancer Sciences, University of Leicester, University Road, Leicester, Leicester, LE1 7RH, UK; Department of Genetics, Genomics and Cancer Sciences, University of Leicester, University Road, Leicester, Leicester, LE1 7RH, UK; Institute of Medical Genetics and Applied Genomics, University of Tübingen, Calwerstraße 7, Tübingen 72076, Germany; European Molecular Biology Laboratory, European Bioinformatics Institute, Wellcome Genome Campus, Hinxton, Cambridge, CV10 1SD, UK; Institute of Medical Genetics and Applied Genomics, University of Tübingen, Calwerstraße 7, Tübingen 72076, Germany; Centre for Rare Diseases, University of Tübingen, Geschäftsstelle Eisenbahnstraße 63, Tübingen 72072, Germany; Centro Nacional de Análisis Genómico, C/Baldiri Reixac 4, 08028, Barcelona, Spain; Departament de Genètica, Microbiologia i Estadística, Facultat de Biologia, Universitat de Barcelona (UB), Diagonal, 643, 08028, Barcelona, Spain; Department of Genetics, University of Groningen, University Medical Center Groningen, HPC CB50, P.O. Box 30001, Groningen, 9700 RB, The Netherlands; Department of Genetics, Genomics and Cancer Sciences, University of Leicester, University Road, Leicester, Leicester, LE1 7RH, UK

**Keywords:** rare disease, genetics, bioinformatics, computational biology, fair data, infrastructure

## Abstract

The Solve-RD project brings together clinicians, scientists, and patient representatives from 51 institutes spanning 15 countries to collaborate on genetically diagnosing (“solving”) rare diseases (RDs). The project aims to significantly increase the diagnostic success rate by co-analyzing data from thousands of RD cases, including phenotypes, pedigrees, exome/genome sequencing, and multiomics data. Here we report on the data infrastructure devised and created to support this co-analysis. This infrastructure enables users to store, find, connect, and analyze data and metadata in a collaborative manner. Pseudonymized phenotypic and raw experimental data are submitted to the RD-Connect Genome-Phenome Analysis Platform and processed through standardized pipelines. Resulting files and novel produced omics data are sent to the European Genome-Phenome Archive, which adds unique file identifiers and provides long-term storage and controlled access services. MOLGENIS “RD3” and Café Variome “Discovery Nexus” connect data and metadata and offer discovery services, and secure cloud-based “Sandboxes” support multiparty data analysis. This successfully deployed and useful infrastructure design provides a blueprint for other projects that need to analyze large amounts of heterogeneous data.

## Background

Solve-RD is a Horizon 2020–supported EU flagship project that brings together >300 clinicians, scientists, and patient representatives from 51 institutes across 15 countries [1]. Solve-RD is built upon a core group of 4 European Reference Networks (ERNs; ERN-ITHACA, ERN-RND, ERN-Euro NMD, and ERN-GENTURIS) and 2 associated ERNs (ERN RITA and ERN-EpiCARE), as well as the Spanish and Italian national Undiagnosed Diseases Programs, which annually see more than 270,000 rare disease (RD) patients with varying pathologies. The main ambition of Solve-RD is to solve unsolved RD cases for which a molecular cause is not yet known. This is achieved through an innovative clinical research environment that introduces novel ways to organize expertise and data. Two major approaches are being pursued: (i) massive data reanalysis of >19,000 experiments (various forms of genetic testing) from individuals affected by a rare condition and their unaffected family members and (ii) combined analysis of diverse types of newly generated data, (“novel” omics data).

For the data reanalysis, ERN partners contributed pseudonymized data (phenotypic data, pedigree information, exome sequencing [ES] data/genome sequencing [GS] data, and associated metadata) for individuals affected by an RD who remained genetically undiagnosed after ES or GS. Data were submitted via the RD-Connect Genome-Phenome Analysis Platform (GPAP) [2]. In addition, novel omics data (short- and long-read GS, short and long-read RNA sequencing, epigenomics, metabolomics, deep-ES, and optical genomic mapping) are being generated by different service providers for cohorts defined by the Data Analysis Task Forces (DATF) from the 4 core collaborating ERNs [1]. Sample submitters from the ERNs upload their pseudonymised phenotypic and pedigree information in the RD-Connect GPAP PhenoStore module. From there, Phenopackets and pedigree descriptions in PLINK PED format are exported and submitted to the European Genome-Phenome Archive (EGA). When novel omics data are generated, the service providers upload them directly to the EGA together with a manifest that links it to the corresponding individual. With such an amount of data to be analyzed in a collaborative manner, downloading and analyzing on local compute facilities is not feasible for all centers. Therefore, also centralized analysis facilities were desired.

All this clearly highlights the project’s need for a supporting data infrastructure, particularly because diverse demographic, phenotypic, and multiomics data need to be securely submitted by a large number of clinical centers and other data providers over a multiyear period. The quality of data and the relationships between data and files need to be captured to enable optimal use of the available data. Furthermore, to enable researchers from different centers to work together on the same dataset, an accessible cloud infrastructure is required for all researchers.

To enable reproducibility of analyses, we organized the datasets in freezes of fixed sets of participants, which were updated with patches containing new information that became available over time. This information is captured within a MOLGENIS database [3, 4] and supplemented with an advanced discovery layer based on Café Variome [5] to enable identification of cases or sets of cases (virtual cohorts) based on a wide array of filters, including phenotypic or genotypic similarity metrics and federation with other RD data and sample resources. In addition, appropriate metadata (e.g., file checksum) are collected to ensure that file integrity is maintained during transfer between research centers. This allows researchers to select samples of interest (e.g., all affected individuals with a specific phenotype) and collect the associated files at their preferred analysis location. Similar discoverability features are available through the RD-Connect GPAP cohorts application. Furthermore, the RD-Connect GPAP is connected to MatchMaker Exchange [6] and the Network of Beacons [7], enabling bidirectional patient matchmaking queries to similar resources around the world.

The Solve-RD project infrastructure has been constructed by leveraging existing data platforms, tools, and standards wherever possible and by creating new tailored implementations where necessary, assembled into an interconnected infrastructure. We have operated on the core principle that we will reuse, enhance, and deploy existing solutions (for core analytics support, databasing, data discovery, and data sharing) wherever possible, according to Findable, Accessible, Interoperable, and Reusable (FAIR) data principles [8]. This article describes the current state of the infrastructure, which is fully operational, and indicates how we are further improving and extending its capabilities to ensure its future relevance and wider utility. We believe the resulting infrastructure could provide a template that future large-scale RD analysis projects can start from. Most of the components are tailored for RD research, but the general design and some components of the infrastructure can also be of use for groups focusing on other topics.

## Results

**Figure 1:**
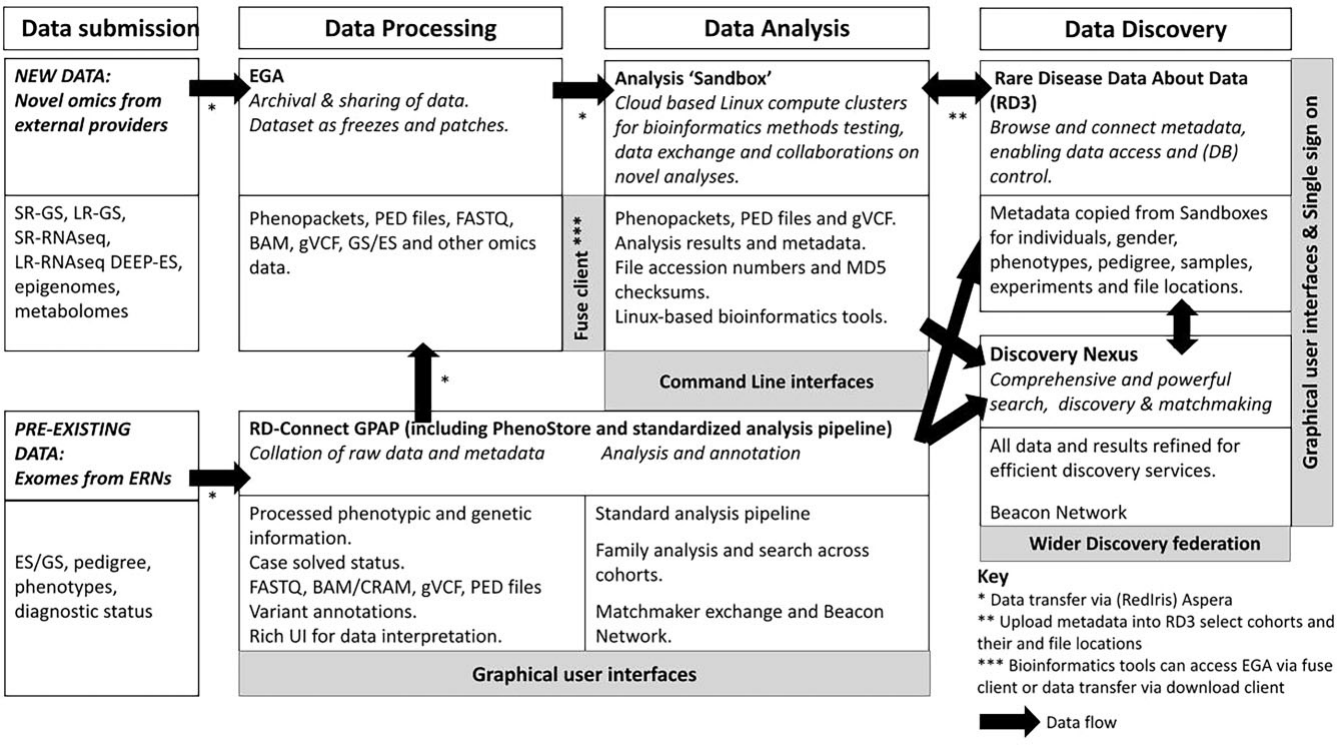
Rare disease analysis infrastructure overview. deep-ES: deep sequencing ES; EGA: European Genome-Phenome Archive; ERN: European Reference Network; ES: exome sequencing; GPAP: Genome-Phenome Analysis Platform; GS: genome sequencing; LR-GS: long-read genome sequencing; LR-RNAseq: long-read RNA-sequencing; SR-GS: short-read genome sequencing; SR-RNAseq: short-read RNA sequencing; UI: user interface. The Solve-RD dataset is also discoverable through the participation of the RD-Connect GPAP in Matchmaker exchange and the Beacon Network.

The data infrastructure we have developed for Solve-RD facilitates submission of input data, a common approach to processing and archiving, collaborative data analysis, and sophisticated data discovery. The overall design and data flow is summarized in Fig. 1.

### Data submission and processing

Experimental metadata are first submitted to the RD-Connect GPAP and corresponding phenotypic data submitted to the GPAP PhenoStore, where patient, phenotypic, and family information are stored. Associated omics and pedigree data files then follow 1 of 2 paths, as described in the Methods. Preexisting sequencing data are submitted to the RD-Connect GPAP, where they are processed through the RD-Connect standard analysis pipeline to homogenize results and facilitate systematic analysis, interpretation, and comparisons [9]. The raw and proceed data can then be downloaded by project partners and processed with a secondary tool (e.g., for the identification of copy number variants or short tandem repeat variants). After processing, raw data, alignments, and detected genetic variants are submitted to the GPAP analysis platform and forwarded to the EGA to be archived. Newly generated novel omics data are archived directly to the EGA. As described in our Methods section, the standard file formats used within our workflow led to easy hand-off capabilities between the different components.

#### Long-term storage

At the EGA, a unique identifier (UID) is added to each individual file and data are made available for download. A manifest file (Supplementary Table S1) with metadata provides background information on the origins of the files to aid in future data interpretation. In parallel, the Solve-RD Rare Disease Data about Data (RD3) database collects data and metadata on subjects, samples, experiments, and files from these sources and makes this available for discovery using the Discovery Nexus service, both described below.

#### Standard processing of reanalysis samples

Sequencing data originating from 43 different research centers were submitted together with a common set of required metadata for each participant and associated experiment. Solve-RD includes fully reanalyzed ES or GS data from 22,326 participants (data freezes 1–3) for whom routine diagnostic procedures failed to achieve a molecular diagnosis. Furthermore, novel omics data from 5,184 participants (2,280 short-read genome sequencing [SR-GS], 510 long-read genome sequencing [LR-GS], 634 short-read RNA sequencing [SR-RNAseq], 80 long-read RNA sequencing [LR-RNAseq], 480 Epigenomics, 930 deep-ES, 270 Metabolomics) have been newly generated and incorporated. All of these data will be fully processed within the project [1; Laurie et al., unpublished observations]. Solve-RD has archived over 750,000 files of primary and processed data at EGA totaling 818 terabytes. Impressively, this represents nearly 5% of all data archived at EGA, the second largest project at EGA to date. The data held by the EGA will be fully available, under controlled access, to the wider RD community, and the ES/GS variant data are available to browse and analyze by any registered RD-Connect GPAP user.

#### Freezes and patches

Data are structured into freezes and patches [1]. The Solve-RD project has generated 3 large freezes that consist of reanalysis data from subjects and experiments that have been submitted prior to 1 of 3 deadlines, meaning that each freeze consists of a fixed number of experiments and participants. The submission closing date for the first freeze was 30 September 2019, and it included data from 8,275 participants. The second and third freezes closed on 30 September 2020 and included data from 3,192 participants. The third freeze closed on 30 September 2021 and included data from 10,516 participants. Changes in data or metadata for these subjects are captured in patches, leaving the original dataset on which analyses have been performed intact, making reanalysis possible. In addition, 2 data freezes for the novel omics data have been generated. For a small number of participants, there were unintended duplications of datasets; a few cases had to be withdrawn from the collection for different reasons. To allow for data changes postsubmission (e.g., addition of new phenotypic information or correction of errors), serial patches were introduced for each freeze. Patched files were released with a date inserted between the preserved filename and its file type extension (i.e., FILENAME.YYYY-MM-DD.extension). For each original freeze or subsequent patch, all data were included in a uniquely identifiable EGA dataset (EGAD).

### Data analysis

Data analysis was performed by DATF teams, and interpretation of variants was done by Data Interpretation Task Force (DITF) teams. DATF activities were divided over several working groups [1] tackling ES and GS reanalysis and processing the newly generated “novel omics” data. Only approved researchers who had signed the project code of conduct (Supplementary Information 1) could access the data. Solve-RD partners can analyze data through 3 main approaches: the RD-Connect GPAP, a cloud-based “Sandbox,” and authorized local clusters.

While a wide range of analyses can be performed using the RD-Connect GPAP user interfaces (as described in the Methods section), new analysis methods to find or interpret new variants and solve cases are continuously being developed. Moreover, for the novel omics data, analysis protocols are not yet standardized and needed to be developed by Solve-RD partners. We therefore needed an extensive analysis infrastructure to enable project analyses. A data request and download option was provided for partners that had their own substantial local compute facilities after approval of the project steering committee.

#### Data management within analysis Sandbox

To support groups that did not have large compute and storage capacity, as well as to enable multicenter collaborative analyses, a centralized analysis “cloud” Sandbox was established. It supports existing and new research methods and allows collection and sharing of project results. The Sandbox approach provides a central analysis environment for bioinformaticians to collaborate and to use and develop new methods freely. Via the Sandbox, DATF and DITF working groups performed pilot studies using newly devised tools to assess their added value before undertaking an analysis of full datasets. The Sandbox functions as a “virtual/trusted research environment” (VRE/TRE) or “safe haven,” providing access to data for analysis while protecting patient confidentiality supported by trained staff and agreed processes [10]. Before users could access any Sandbox content, a project Code of Conduct had to be signed and approved.

The Solve-RD Sandbox provides a Linux-based high-performance computing (HPC) environment suited to bioinformaticians. To provide failover, we have deployed the Sandbox on 2 separate clouds. The Sandbox supports large-scale data storage organized as a high-performance temporary (tmp) section and a stable but slower back-upped permanent (prm) folder. The tmp folder supports data analysis and so has a free structure for individual users to manage. The prm folder has a fixed structure that was identical at both Solve-RD Sandboxes.

Within each of the 2 VREs, the tmp folder includes a single master folder containing original freeze files as well as patched files. For each freeze and patch, a folder exists that carries symlinks to the files included in the specific patch release, typically a mix with the majority of files included in the previous patch and some new changed files. Because of limited storage space, not all files from the project could be simultaneously held in the ​Sandbox. Therefore, larger files were omitted and reloaded as and when needed. In addition to these folders, an ega-fuse-client folder was present in the prm folder, giving direct access to the Solve-RD datasets archived at the EGA. This enables the large files to be accessed from within the VRE, even though no local copy was present.

**Figure 2:**
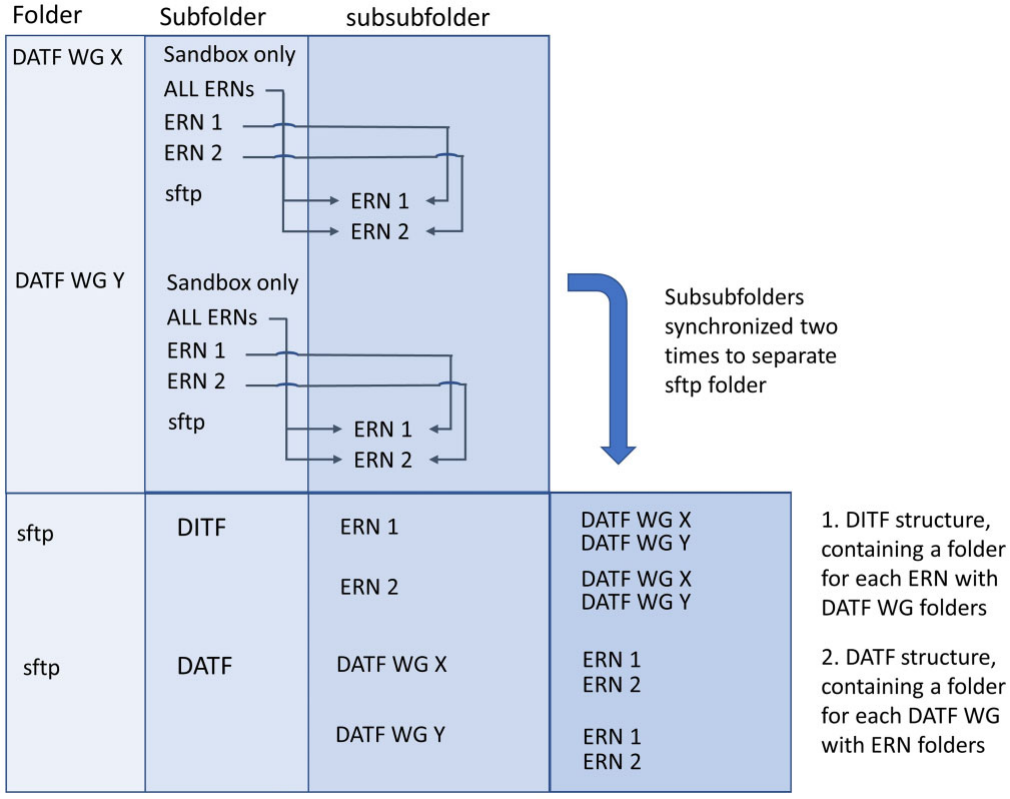
Sandbox folder structure. Data are organized by the data analysis working groups (DATF working group [WG]) in either folders per European Reference Network (ERN) or a common folder (for data intended for all ERNs). Additionally, large files that should be kept but not shared are stored in a “Sandbox only” folder. All data to be shared with the ERNs are linked to an sftp folder with a subfolder per ERN accessible via SFTP access protocol. Thin arrows indicate links between specific subfolders. These folders are further synchronized to 2 folders: DATF and DITF, each with the same information (indicated by the thick arrow). The DATF folder has the same structure as the initial sftp folder (a folder for each DATF WG with subfolders per ERN). The DITF folder has the converse structure (a folder for each DITF ERN with subfolders per WG). This structure makes it easy for both DATF and DITF to browse the data (e.g., all CNV data or all data from ERN-ITHACA).

To provide access to analysis results, a dedicated directory was created for each DATF working group. To store their analysis results, each DATF working group appointed a data manager who was allowed to copy, move, and remove data to and from the prm folders on the VREs (automatically synchronized between the 2 VRE instances). The folders were structured such that data sharing was optimally facilitated (Fig. 2).

### Data discovery

Many diverse data types and files exist within the Solve-RD project (multiomics, variant interpretation, phenotyping, demographics, etc). These are stored in different places and in different formats. The totality of metadata can be navigated via the RD3 database, based on the MOLGENIS technology [3, 4]. Via RD3 and the advanced discovery layer “Discovery Nexus,” DATF bioinformaticians can find samples and data of interest. To do this, they formulate queries that identify file identifiers (EGAF) for relevant data stored in EGA, to then access these data in the sandbox, in GPAP, or in their local cluster.

Additional data discovery functionalities are provided by the RD-Connect GPAP, as described in [2]. These consist of an internal “search across all” functionality, allowing users to search for specific types of variants in candidate genes of interest across all experiments. This can be further refined using the “cohorts” application, which allows identification of affected individuals with similar phenotypes within the RD-Connect GPAP, including data not submitted as part of Solve-RD. The RD-Connect GPAP is also an active node in the international MatchMaker Exchange network, facilitating patient matchmaking worldwide [6], and has also created a beacon within the Global Alliance for Genomics and Health (GA4GH) Beacon Network [11].

#### RD3–tracking files and metadata

**Figure 3:**
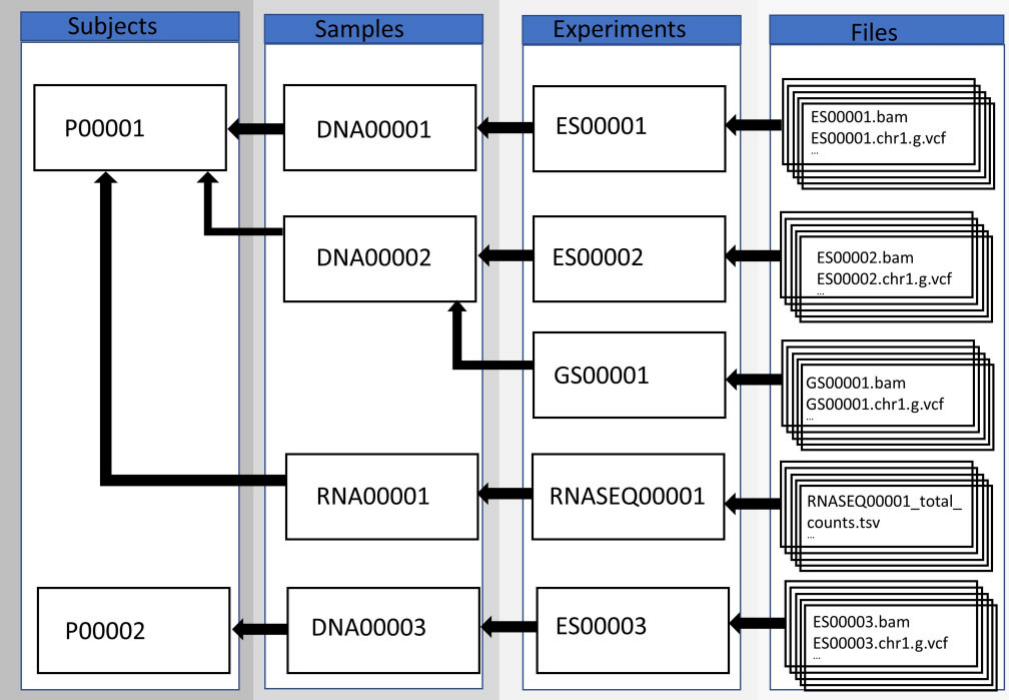
Data and metadata relations within Solve-RD. Arrows indicate the “derived from” direction (e.g., Sample DNA00001 is derived from Subject P00001). We distinguish 4 main data/metadata types: subject, sample, experiments, and files, with each derived from the former. This figure is actually a simplification as data are further organized in data releases we call “freezes” and can be used in different combinations as “analyses.”

Direct data navigation is supported by the “rare disease data about data” (RD3) system. This MOLGENIS database provides a complete listing of all patients/participants, samples, experiments, and data files in Solve-RD, including EGA UIDs. The data model of the Solve-RD project describing how data are organized is summarized in Fig. 3.

Some relationships are direct, such as the subject–sample relation (a sample is derived from a subject), whereas others are not so obvious and need to be discovered. RD3 is tightly integrated with Discovery Nexus, which also leverages useful extractions of various data files (e.g., extant variants, their frequency, host gene, mutation type, etc). Following a successful Discovery Nexus search, suitably permissioned users can click through to RD3 directly to access the discovered data files.

#### Discovery Nexus

Discovery Nexus supports data discovery via a range of approaches that help users initially establish the existence and location (rather than the substance) of data within the system. The interface provides filtering options by which users can distill a comprehensive overview of selected datasets that might be of value for their intended purpose. Querying by multiple data values is possible, driven by ontologies and ontology cross-mappings. Searches can look for identity or semantic similarity to an entered term, or any combination of terms, and even extend to bridging between concepts (e.g., searches by biochemical pathway leverage knowledge of which genes are in each pathway). It also supports the GA4GH standard Beacon-2 Application Programming Interface (API) [7] for wider interoperability.

## Discussion

To enable numerous researchers and clinicians to work together in parallel on a large dataset in Solve-RD, it was essential to establish a good data infrastructure that adheres to FAIR principles (see Box 1). The solution we created includes access policies and procedures, including the code of conduct (Supplementary Information 1), a network of databases, HPC clusters, long-term storage capabilities, federated discovery services, and tools and pipelines to provide the project with the ability to solve many RD cases that had not been solved using conventional strategies. The infrastructure can be used starting from 3 main goals: data submission and processing, data analysis, and data discovery, as described in the Results and Methods. For each of these goals, the most typical workflow is shown in Supplementary Fig. S1. Depending on the user, different parts of the infrastructure are used. Typically, clinicians will submit samples, whereas the researchers, split between the DITF and DATF, will set up cohorts of patients with similar phenotypes, find different types of genetic variants through various analyses, and zoom in on possible causal genetic variants. Using this infrastructure, the Solve-RD project has already made >500 new diagnoses [Laurie et al., unpublished observations], and many analyses powered by novel omics data are still ongoing.

The 2 parallel tracks, reanalysis of existing ES or GS data and novel omics data analysis, each created distinct challenges. One of the main challenges of the exome reanalysis stemmed from the heterogeneity of the submitted data. Cases were provided by institutions all around Europe, and exomes were enriched using various designs and versions, as well as sequenced using different short-read platforms, each of which will result in different biases. In addition, analyses prior to submission to Solve-RD had been performed using a range of different alignment and variant-calling algorithms. To facilitate data integration, the Solve-RD project reanalyzed primary sequence data from the earliest possible point, using a standardized workflow, thereby eliminating bioinformatic-related differences and providing a coherent set of files for each of the experiments submitted. In parallel, the RD-Connect GPAP processed participant metadata and pedigree information and exported these in standard file formats. This provided reusable and interoperable data enabling downstream analysis via the RD-Connect GPAP, the project Sandboxes, and local clusters.

Regarding novel omics, the main challenges from the perspective of the infrastructure were the different types of files produced and differences in accompanying metadata, which required a custom-made database format to capture these data.

Box 1: FAIR components of the Solve-RD infrastructure

**Findability**

- Infrastructure components are findable through bio.tools (GPAP), GitHub (RD3, Discovery Nexus, sandbox).
- Raw data have globally unique identifiers (EGAD and EGAF).
- Samples of interest are findable through RD3/Discovery Nexus.
- Structuring results by DATF and DITF allows data to be findable based on both technique and disease.

**Accessibility**

- Archival of files in the EGA ensures long-term accessibility of raw data.
- Phenotypic and variant data are accessible to registered users via GPAP.
- Metadata are stored separately from data through a manifest file.
- Aspera servers, ega-fuse-client, and download client can be used to transfer data to and from the EGA.
- Having multiple clusters accessible by all project members ensures accessibility of a data analysis infrastructure in case of maintenance.

**Interoperability**

- New HPC clusters can be deployed using Ansibl playbooks.
- Processing data starting from raw data using a standardized pipeline maximizes uniform output data.
- Output and export files follow file and ontology standards where possible.
- Seamless integration of RD3 and Discovery Nexus.
- GPAP provides interoperability via multiple APIs, including Beacon-2 MatchMakerExchange, Ensembl, OMIM, and Orphanet.

**Reusability**

- The creation of file patches allows for older versions of files to remain usable for reanalysis.
- Informed consent allows for data analysis after data access committee approval to data stored at EGA.

Data FAIRness was enhanced by placing the data within the EGA data archive for long-term storage, request, and access. To maximize user convenience, single sign-on capability was provided across different components supporting a single goal, such as RD3 and Discovery Nexus, or between the Sandboxes and EGA via the filesystem in userspace (FUSE) client, as described in the Methods. We also developed innovative methods to make data findable before and after data access is granted, using Discovery Nexus for preliminary searches (interoperable with GA4GH Beacon technology) and the RD3 database for full dataset navigation. Once the Solve-RD funding period is over, this same service will enable ERN data owners to advertise their data to researchers outside the project without directly releasing data too liberally or before access requests are reviewed and data sharing agreements set up. The data discovery service will also provide potential users with sufficient insight into the nature of available datasets to be confident that it is worth investing effort to request and analyze the data. The Solve-RD omics data (i.e., preexisting unsolved exomes and genomes as well as omics data generated within the project) are archived in the EGA (Supplementary Information S2) and will be made available to other rare disease researchers via a controlled access mechanism, governed by the Solve-RD data access committee (DAC). The DAC consists of 1 representative per ERN that contributed data and/or samples to Solve-RD as well as a patient representative. Researchers who would like to access a specific Solve-RD dataset need to request access from the Solve-RD DAC. To do this, they have to fill in and sign the Solve-RD data access agreement (DAA) (Supplementary Information S3) [12] and send it to the DAC office. The DAA lays out the terms under which access to Solve-RD data (including sequence and genotype data, other omics data, phenotypic data, and pedigree information) is being granted.

Within projects such as Solve-RD, concrete analyses are often conceived after the collection of data. This reflects the continuous expansion of associated knowledge and support tools. To facilitate this, we emphasized structured collection of rich metadata, thereby making the available data unambiguous in terms of its scope, quality, provenance, and location. RD3 was used to organize and provision these metadata, following FAIRGenomes guidelines [13]. In addition, the RD-Connect GPAP co-hosts sections of the metadata relevant to their content, and these metadata also allow cohort-building via both Discovery Nexus and the RD-Connect GPAP.

In conclusion, Solve-RD has devised, implemented, and validated an infrastructure for bringing together a set of reusable tools and best practices. As Solve-RD partners continue to use the infrastructure to perform many multiomics analyses, the operational support teams are actively working together with related projects, ensuring sustainability and further development of the different infrastructure components. For example, some of the components are being deployed and expanded in European projects such as the European Joint Programme on Rare Diseases (EJP-RD [14]), the EU Genome Data Infrastructure project (GDI [15]), and national initiatives such as the Dutch FAIR genomes/Health-RI genomics project [16]. Ongoing projects, such as GDI and the forthcoming ERDERA [17], support sustainability and future developments of the components for the future. Hence, the infrastructure described in this article can be used as a blueprint for future multiomics data (re)analysis projects and data hubs.

## Methods

The Solve-RD infrastructure consists of various interconnected parts, each playing a role in different workflows needed required by the project (Supplementary Fig. S1). The components described in the sections below are listed in Table 1.

**Table 1:** Components Solve-RD project infrastructure

Component	Version	Repository	License	Documentation	Registration
RedIris Aspera		https://github.com/IBM/aspera-cli	BSD-3-Clause	https://www.rediris.es/rediris/	
RD-Connect GPAP and PhenoStore	2.28.0	https://platform.rd-connect.eu	NA	https://platform.rd-connect.eu/gpap_doc/	https://bio.tools/rd-connect_platform
Standardized Analysis Pipeline	20210521	https://github.com/inab/Wetlab2Variations/	Apache-2.0	https://pubmed.ncbi.nlm.nih.gov/27604516/	https://workflowhub.eu/workflows/107
MOLGENIS	10.1.0	https://github.com/molgenis/molgenis	GNU LGPL-3.0	https://github.com/molgenis	https://bio.tools/molgenis
Sandbox (deployment)	23.04.1	https://github.com/rug-cit-hpc/league-of-robots	GNU GPL v3.0	https://docs.gcc.rug.nl/fender/	
Sandbox (Ansible pipelines)	1.1.0	https://github.com/molgenis/ansible-pipelines	GNU GPL v3.0		
RD3 database	v1.0	https://github.com/molgenis/RD3_database	GNU LGPL-3.0		
RD3 solve-rd	v1.0	https://github.com/molgenis/projects-solve-rd	GNU LGPL-3.0	https://solve-rd.gcc.rug.nl/	
Cafe Variome	2.3.2	https://github.com/Cafe-Variome/CafeVariome/	MIT	https://cafe-variome.gitbook.io/	
Discovery Nexus	v2.0.0-alpha	https://github.com/Cafe-Variome/RDNexus	MIT	https://cafe-variome.gitbook.io/	
Beacon-2	cineca.2021.03	https://github.com/Cafe-Variome/beacon-2.x	Apache-2.0	https://docs.genomebeacons.org/	
MatchMaker Exchange	V1.1.1	https://github.com/ga4gh/mme-apis	NA	https://www.matchmakerexchange.org/	https://bio.tools/matchmaker_exchange
downloadclient (pyEGA3)	5.1.0	https://github.com/EGA-archive/ega-download-client	Apache-2.0	https://ega-archive.org/access/download/files/pyega3/	
ega-fuse-client	3.0.0	https://github.com/EGA-archive/ega-fuse-client	Apache-2.0	https://ega-archive.org/access/download/visualisation/fuse-client/	

### Data submission and processing

Many types of data were provided by the ERNs or newly generated within the Solve-RD project, including demographic and phenotypic data of participants and metadata on samples, experiments, and files. Preexisting sequencing data are submitted to the RD-Connect GPAP as FASTQ [18], BAM [19], or CRAM [20] files via a RedIris Aspera server. Specifically, ES and GS reanalysis data and metadata were provided by partners of 6 different ERNs: ERN-ITHACA, ERN-RND, ERN-Euro NMD, ERN-GENTURIS, ERN RITA, and ERN-EpiCARE. For novel omics analysis, various other file types and concomitant metadata were produced.

Raw ES and GS read data for reanalysis, together with accompanying metadata and deep phenotypic descriptions of affected individuals, were submitted by Solve-RD partners to the RD-Connect GPAP (GPAP). Alignment and short variant calling was undertaken for all experiments using an identical variant calling workflow [9] to minimize bioinformatics-induced artifacts. All identified Single Nucleotide Variants (SNVs) and indels were made immediately available to Solve-RD collaborators for analysis in the GPAP Genomics module. Subsequently, the raw data and processed data in the form of BAM/CRAM and gVCF files were transferred to the EGA for longer-term archival and redistribution to other Solve-RD partners.

GPAP was used for collation of all phenotypic data and standardized processing of all short-read ES and GS data submitted to Solve-RD. Data collation was undertaken as described in Laurie et al. [2].

Briefly, in the first step, pseudonymised phenotypic descriptions of all affected individuals were uploaded to the RD-Connect GPAP PhenoStore module, using Human Phenotype Ontology (HPO), OMIM, and Orphanet terms to generate a detailed phenotypic description, together with a family tree linking individuals. Each individual receives a unique participant ID (P-ID), and for accompanying experiments, experiment IDs (E-IDs) were created. The relation between P-ID and full personal identifiers is known only to the submitter. In the second step, metadata describing the raw sequencing data to be submitted for reanalysis and linking them to the individual’s phenotypic record are uploaded to the GPAP Data Management module. Finally, the raw sequencing data themselves are transferred using a robust, high-speed Aspera data transfer service provided by RedIris, the Spanish academic and research network [21]. Once submission is complete, the data are automatically ingested and processed by the automated standard analysis pipeline.

#### Standard analysis pipeline

For joint data analysis, it is important that technical differences between experiments are minimized. Therefore, using the CNAG-CRG local HPC resources, all short-read ES and GS data submitted to Solve-RD were reprocessed using an identical standardized variant calling pipeline as described in Laurie et al. [9].

#### Data sources for preexisting and new data

For reanalysis of ES/GS, novel omics short-read (SR) GS, and deep-ES data, the starting point for reanalysis was the associated FASTQ files. When BAM or CRAM files were submitted, these were first transformed back to FASTQ. Using the standard analysis pipeline (Fig. 1), data were processed in a standardized manner as described above, producing a single BAM and 25 g.VCF files (autosomes, X, Y, and mitochondria), accompanied by .BAI and .TBI index files, respectively. Phenotypic information was exported from GPAP in Phenopacket format and pedigrees in PED file format. LR-GS files and RNA sequencing data were stored in BAM format. Data analysis produced output of various file formats, depending on the tools used for analysis.

#### Interoperability

To maximize interoperability for tool integration and reuse beyond Solve-RD and to overcome language barriers, we use widely adopted and machine-readable international and community standards and ontologies whenever possible. Within PhenoStore, deep phenotypic descriptions are recorded using Human Phenotype Ontology [22], Orphanet Rare Disease Ontology (ORDO) [23], and the Online Mendelian Inheritance in Man (OMIM) [24] terminology. Phenotypic records can be exported using the GA4GH approved Phenopacket format [25] and family trees in PLINK PED format [26, 27]. Genomic alignments are stored and transferred (e.g., to the EGA) in GA4GH-approved BAM, CRAM formats [18, 19, 28]. Variants are stored in GVCF format [29]. Biological annotations, available in the Data Analysis module, are provided by Ensembl VEP [30] and supplemented with data from other genomics community resources such as ClinVar (RRID:SCR_006169) [31], gnomAD (RRID:SCR_014964) [32], and PanelApp [33]. Data discovery and sharing is achieved through the implementation of GA4GH Beacon-V2 [7] and Matchmaker Exchange (MME) APIs [6]. Partner involvements in other initiatives also guided our work regarding other standardization strategies, not least Beyond One Million Genomes, GA4GH, FAIR genomes [13], European Life Sciences Infrastructure (ELIXIR), Biobanking and Biomolecular Resources Research Infrastructure, and EJP-RD.

#### EGA long-term data archiving and access

The EGA [34] is a service for permanent archiving and sharing of identifiable genetic and phenotypic data [35, 36]. Data archived at the EGA ensures long-term availability, interoperability, and identifiability during projects and beyond. The primary objects in the EGA data model are studies, datasets, and files (raw and processed). Each archived file is assigned an EGA accession (EGAF) functioning as a UID. Moreover, each file can be part of one or more datasets, each with its own accession number. After data are successfully archived and released, the EGA provides access to the data only upon approval by the associated DAC for specified individuals. Datasets can be accessed using the PyEGA3 streaming client [37] and a FUSE client [38].

To ensure data are FAIR, metadata are uploaded to EGA alongside data files. These metadata take the form of manifest files (Supplementary Table S1), which contain many attributes describing the data, for example, what library preparation and sequencing strategy was followed; what type of data analysis was done, including which reference genome was used; and minimal public information about the study subjects. Manifest files are converted to the EGA XML standard for representing metadata before being permanently archived. To guarantee data security and preservation of data integrity during file transfer and archival at EGA, data files are submitted to EGA in an encrypted format, and file checksums are compared at different points of the submission process. For example, encrypted file checksums are compared before and after upload to the EGA to ensure that the file was not corrupted during transfer. After being reencrypted at EGA with a symmetric key and stored in the permanent archive, 1 final checksum check is performed to ensure integrity of the permanently archived, encrypted file.

### Data analysis

#### RD-Connect GPAP

The RD-Connect GPAP allows users to perform variant analysis to identify potential disease-causing variants in a single proband or any family structure and allows user-defined queries across a cohort of affected individuals. These capabilities are provided via a user-friendly interface suitable for clinicians, genome scientists, and bioinformatics researchers.

A large variety of filters can be applied to identify known pathogenic variants (e.g., described in ClinVar) or prioritize variants that are potentially pathogenic for further investigation [2]. Furthermore, variants can be visualized in remotely hosted native BAM files on-the-fly, directly within the GPAP, through implementation of the GA4GH htsget streaming protocol and a client-side Integrative Genomics Viewer instance [39].

Analysis can be undertaken in 2 different ways, either interactively via a graphical user interface (GUI) or automated via a Python-based API. The interactive approach is ideal for analyzing individual families and applying different filter strategies. For processing large numbers of experiments, as undertaken in Solve-RD, programmatic batch analysis can be undertaken as described previously [40].

Intra-GPAP case-matching is possible via an instance of the GA4GH MME API [41] and by searching across cohort functionalities. External case matching can be achieved through the global MME API [6], and single variants can be found via the Beacon-V1 API [11].

#### Sandboxes for bespoke bioinformatics analyses

Bioinformatics methods often require a Linux command-line environment and extensive computing and storage capabilities. In line with this, we implemented 2 Sandboxes as Linux-based HPC clusters that can be remotely accessed and act as a VRE/TRE. To enable reproducibility and reusability (i.e., in future projects), these Sandboxes are implemented as a “cloud” service that can be automatically deployed at different cloud providers using the same playbook [42], using OpenStack for virtualization of Linux CentOS7 [43] with Spacewalk [44] for package distribution and management and using the LMOD module system [45] and Easybuild [46] to reproducibly install bioinformatics tools.

Because HPC systems typically need large maintenance windows where the service is offline, we have 2 separate Sandbox installations at different locations to prevent a single point of failure and ensure continuous operations to the partners: one at EMBASSY [47, 48], hosted by the EMBL European Bioinformatics Institute (EMBL-EBI), which has close connections to the EGA, and one at the University of Groningen Centre for Information Technology [49, 50] attached to the University Medical Centre Groningen. The EMBASSY VRE is only accessible by members of the Solve-RD project, while the UMCG VRE is a larger facility shared with other projects beyond Solve-RD. A dedicated Solve-RD group is present in the UMCG VRE with access restricted to Solve-RD members only. The EMBASSY VRE has 40 Tb of storage and 12 compute nodes with 14 cores/node and 56,072 Mb RAM/node. The UMCG VRE [51] has shared storage with other projects, with 200 Tb reserved for the Solve-RD project and a total of 10 compute nodes available with 22 cores/node and 205,490 Mb RAM/node.

#### Access to analysis results

Both clusters use internal networks that are not directly accessible from the internet. Access is possible via dedicated jumphosts, security-hardened machines not involved in any data storage or processing. Using asymmetric cryptography via a private–public key pair [52], users can log in to the jumphost to be directly redirected to the main HPC cluster. To allow for data access for non-bioinformaticians, we created an SFTP transfer server that could be accessed using a graphical user interface such as WinSCP [53], MobaXTerm [54], or Cyberduck [55] via a private–public key pair without the extra security of a jumphost.

### Data discovery

#### MOLGENIS RD3

To manage metadata on subjects, samples, experiments, and files of ES reanalysis and novel omics, we used the MOLGENIS RD3 database. A specific Solve-RD instance of this was created [56], accessible via a web interface [57]. In this database, metadata (e.g., file accession numbers) and data (e.g., average coverage for ES targets) are collected for all Solve-RD subjects and the associated samples, experiments, and files.

Content includes information on how samples were collected and the subjects they came from, as well as the analyses that were performed and the location of the files generated. RD3 acts as a hub for GPAP data on Solve-RD participants, data provided by the EGA, files located in the Sandbox, and metadata required for the Discovery Nexus tool. Using portal tables, relevant data and metadata are imported into RD3 using a manifest file provided by the EGA.

RD3 was built in MOLGENIS [3, 4], an open-source database platform for storing, managing, analyzing, and sharing data. Approved users can log in using a local login or through FusionAuth [58]. All the relevant metadata for the research are collected within the Solve-RD RD3. The core structure of RD3 consists of several tables matching the different types of information that should be selected (Fig. 3). ES reanalysis data were imported into RD3 using a system of freezes and patches as described in the Results. Each of these sections has the same format.

The subjects table contains information on the participants as collected in GPAP PhenoStore, imported via phenopackets and PED files archived at the EGA. Subjects are identified based on their P-ID. For each subject, the P-IDs of the parents are given if they were included in the project, as is the family number to identify all subjects who are part of the same family. Furthermore, the subject’s sex and a disease name or the phenotypes known to be present (or absent) are listed. For each subject, it is recorded if they are considered affected by a condition or not (e.g., a child is affected and both parents are unaffected by a condition). In addition, information is stored on the case submitter (e.g., if they are allowed to be recontacted in case of incidental findings or if the case is retracted). Finally, the subjects table shows if the sample is solved. Because this information is updated in the GPAP PhenoStore, a connection between the 2 programs allows the solved status to be updated daily.

Zero or more samples may be derived from each subject. Sample metadata are collected in the samples table. Each sample is given a sample ID (S-ID) for unique identification. Per S-ID, the P-ID of the subject from which it is derived is shown as well as the tissue type (e.g., whole blood) and other sample specifications.

**Figure 4:**
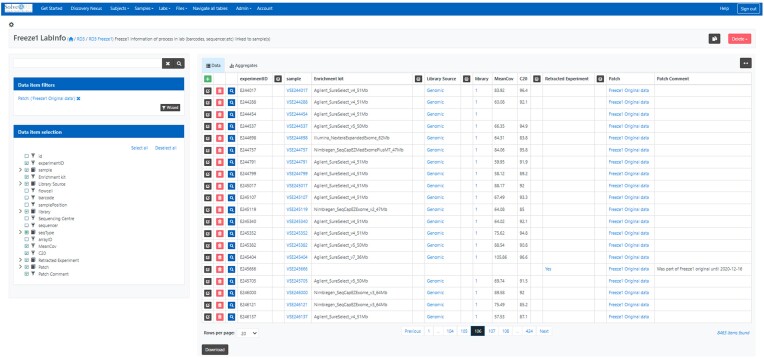
Solve-RD RD3 LabInfo screen showing a subset of the Freeze1 experiment data. On the left, entries are filtered on patch “Original data” and columns are filtered on interest. In the current view, the experimentID is connected to the sample on which the experiment was performed. In addition, information on the experiment is shown. For these samples, genomic data were the input for exome sequencing experiments on which various different enrichment kits were used. For most of the samples, statistics on the average target coverage (MeanCov) and number of bases covered by at least 20 sequencing reads (C20) was available. If a subject was retracted from the project, all metadata except identifiers were removed from the database and the experiment was labeled as retracted.

Zero or more experiments can be performed on each sample (e.g., ES on DNA isolated from the sample). Information on these experiments is collected in the experiments table (see Fig. 4). Each type of experiment has its own specific layout. For ES, the enrichment kit used is captured, as is the sample preparation method. The metrics “% of the target covered >20×” and “average target coverage” are also collected.

For each family, subject and experiment files are archived at the EGA. RD3 captures this information in the files table. Here, for each file, the path in the Sandbox and the VRE ega-fuse-client within the dataset are given with its checksum information enabling a sanity check on copies of this file. Information is recorded about the filetype, the experiment it belongs to, and the EGA accession number.

### Discovery Nexus

RD3 is seamlessly integrated with Discovery Nexus using a single sign-on option based on the open ID connect protocol (open ID connect [OIDC], implemented using FusionAuth), which is compatible with the life sciences authentication and authorization infrastructure (AAI), previously known as ELIXIR AAI [59], which we plan to implement in the future. The latter will enable users to sign in using their institute sign-in, which increases security and General Data Protection Regulation compliance and ensures removal when contracts terminate.

**Figure 5:**
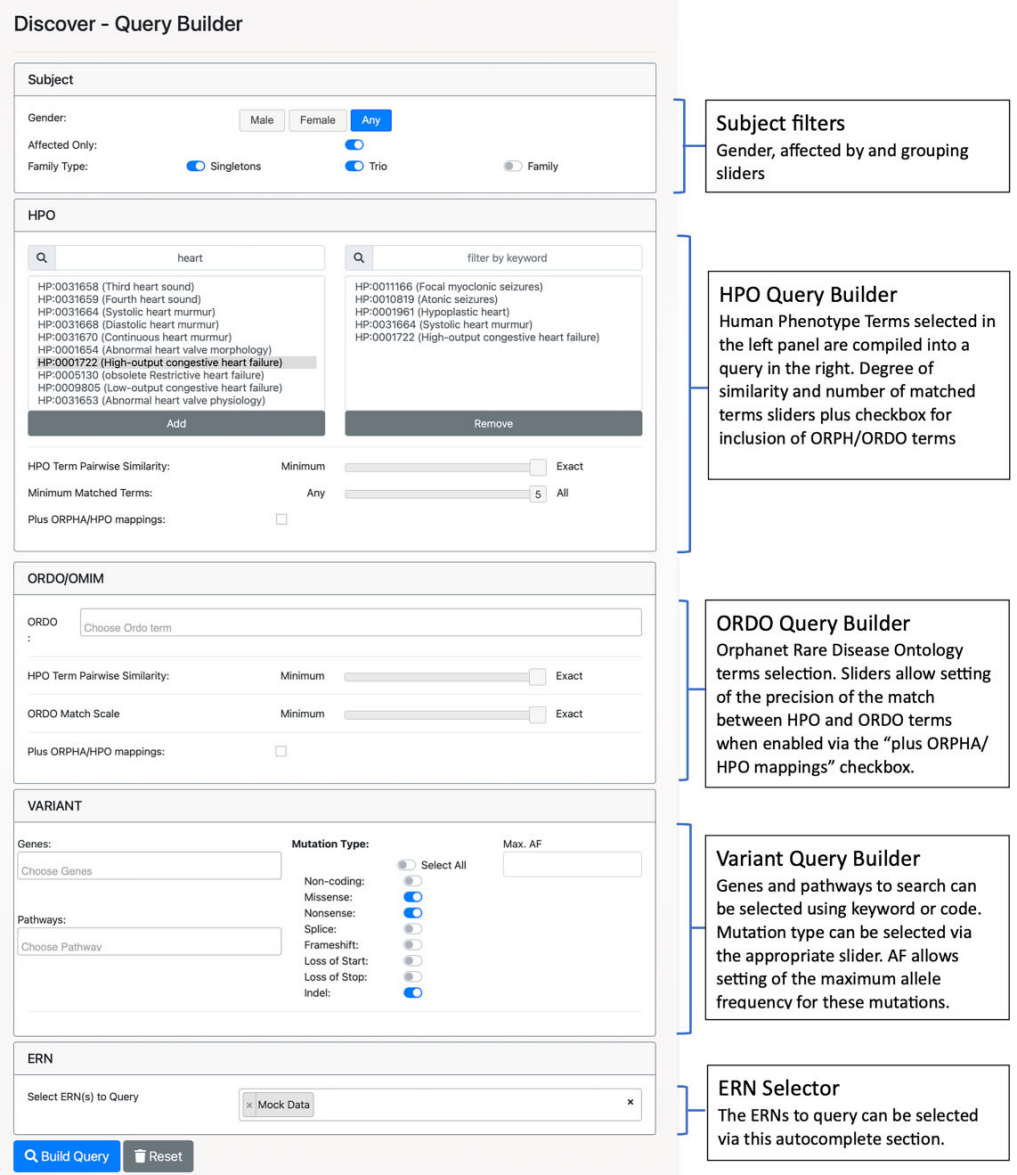

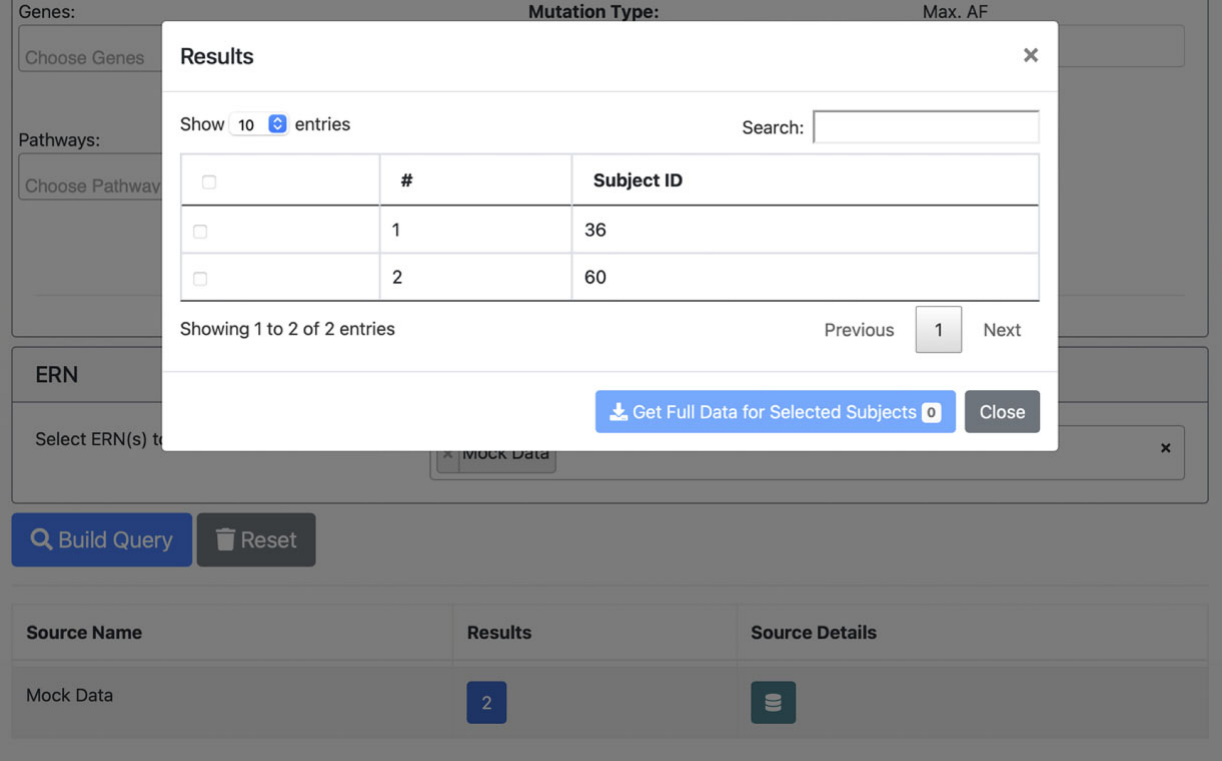
(A) Discovery Nexus query interface. This interface supports querying by any combination of various demographic and inheritance (Subject Filters), phenotypes (HPO Query Builder), diseases (ORDO Query Builders), or suspected variant filters (Variant Filter). In the HPO Query Builder, typing any part of an HPO phenotype term or code creates a visible list of relevant items to select from, whereupon they are transferred into the adjacent panel to form part of the query. Phenotype matching can specify matching on identical terms only (exact) or recover similar terms (based on a precomputed matrix of relationship scores and the position of the slider). The minimum number of matching terms can also be specified, creating an “OR” query, and settings above the minimum create a query that returns results that match at least the specified number of terms in any combination. HPO queries can also be instructed to interrogate phenotype data stored as ORDO terms. Matching of HPO to ORDO terms (in the ORDO Query Builder) is controlled by the HPO pairwise similarity slider, to define the number of HPO terms that should match an ORDO term as well as the ORDO match scale, defining the specificity of the HPO term(s) to the selected ORDO term (based on a precomputed matrix of their occurrence across all ORDO terms). Hence, when mapping ORDO to HPO terms, exact matching will traverse the mapping of these 2 term sets to find fewer but more specific HPO terms, while minimum matching will include more HPO terms, but these may match other ORDO terms as well. Variant data cannot be filtered at the specific base-change level (as this would raise privacy concerns) but are instead queryable by host gene, allele frequency, and mutation type using the Variant Query Builder. It is also possible to filter for variants based on affected biochemical pathways, given known relationships between genes and pathways (using the Reactome Knowledge base [60]). Finally, the ERN dataset to be queried must be explicitly stated and requires that the user has permission to query the specified ERNs. (B) Discovery Nexus Query Results. After submitting the query using the “Build query button,” the system will return a count for matching results in the resources selected. Clicking on the number in the blue box will bring up the summary pop-up window as shown above, giving basic details of the matches (again subject to the user having been assigned permissions). The blue “Get Full Data for Selected Subjects” will open a link to request access from the resources holding the required data (where this is available). Alternatively, clicking the green button in the source details will open a summary page with contact details for the resource, where a direct link to request the data is not available.

Discovery Nexus is a parallel component to RD3 that provides advanced and more powerful capabilities for quickly and deeply searching Solve-RD data stored in different locations and formats. Built on Café Variome [5], Discovery Nexus abstracts direct database-style queries to concept-based queries; for example, phenotypes and diseases are based on common ontologies that Discovery Nexus dynamically maps to ontologies and hierarchies within ontologies used in the underlying subject phenotyping. This is also extended to querying using semantic similarity between and across ontologies. This abstraction allows Discovery Nexus to represent searches in an intuitive query builder interface focused on elements that make queries based on demographics, phenotypes, diseases, variants, biochemical pathways, mutation characteristics, solved-or-not status, and data availability (Fig. 5). This separation of query from database language also provides protection to subjects and studies identification as the actual data are not queried or represented in the query or results. For example, variants are not directly queried in Discovery Nexus; instead, the query interface allows searches for types of variant mutations in genes or gene families.

#### Handoff from Discovery Nexus to RD3 to get data

Discovery Nexus and RD3 operate under a federated single sign-on for authentication using the industry standard OIDC provided by RD3, with only users authorized by Solve-RD able to access either application. This allows the 2 parallel systems to interoperate seamlessly, with a handoff facility allowing search results in Discovery Nexus to be prepopulated in RD3, so that the user can access information about the underlying data without logging in again.

## Supplementary Material

giae058_GIGA-D-23-00271_Original_Submission

giae058_GIGA-D-23-00271_Revision_1

giae058_GIGA-D-23-00271_Revision_2

giae058_Response_to_Reviewer_Comments_Original_Submission

giae058_Response_to_Reviewer_Comments_Revision_1

giae058_Reviewer_1_Report_Original_Submission Anna Bernasconi, Ph.D. -- 1/6/2024 Reviewed

giae058_Reviewer_1_Report_Revision_1 Anna Bernasconi, Ph.D. -- 4/7/2024 Reviewed

giae058_Reviewer_2_Report_Original_Submission Bernie Pope, Ph.D. -- 1/15/2024 Reviewed

giae058_Reviewer_2_Report_Revision_1 Bernie Pope, Ph.D. -- 5/9/2024 Reviewed

giae058_Supplemental_Files

## Data Availability

Data are deposited at EGA. All raw and processed data files will be made available at the EGA (Solve-RD study EGAS00001003851) upon approval by the data access committee. Access can be requested via the document in Supplementary Information S2: Data Access Agreement. Accession numbers of additional datasets will be made available on Solve-RD website [12]. Current datasets are listed in Supplementary Information S3: Dataset-Specific Conditions. Pseudonymized phenotypic information for all individuals and their genetic variants is accessible through the RD-Connect GPAP [61] upon validated registration. The ethics committee of the Eberhard Karl University of Tübingen gave ethical approval for this work.

## Additional Files

**Supplementary Table S1**. EGA manifest file.

**Supplementary Fig. S1**. Common workflows.

**Supplementary Information S1**. Solve-RD Code of Conduct.

**Supplementary Information S2**. Dataset-specific conditions.

**Supplementary Information S3**. Data Access Agreement.

## Abbreviations

AAI: authentication and authorization infrastructure; API: Application Programming Interface; C20: number of bases covered by at least 20 sequencing reads; DAA: data access agreement; DAC: data access committee; DATF: Data Analysis Task Force; DITF: Data Interpretation Task Force; EGA: European Genome-Phenome Archive; EGAD: EGA dataset; EGAF: EGA file identifiers; E-ID: experiment ID; EJP-RD: European Joint Programme on Rare Diseases; ELIXIR: European Life Sciences Infrastructure; ERN: European Reference Network; ES: exome sequencing; FAIR: Findable, Accessible, Interoperable, and Reusable; FUSE: filesystem in userspace; GA4GH: Global Alliance for Genomics and Health; GDI: Genome Data Infrastructure project; GPAP: RD-Connect Genome-Phenome Analysis Platform; GS: genome sequencing; GUI: graphical user interface; HPC: high-performance computing; HPO: Human Phenotype Ontology; LR: long read; MME: Matchmaker Exchange; OIDC: open ID connect; OMIM: Online Mendelian Inheritance in Man; ORDO: Orphanet Rare Disease Ontology; P-ID: participant ID; prm: permanent storage; RD: rare disease; RD3: Rare Disease Data about Data; SR: short read; tmp: temporary storage; TRE: trusted research environment; UID: unique identifier; VRE: virtual research environment.
