# Supplementary material for: An interconnected data infrastructure to support large-scale rare disease research
Source: Gigascience. 2024 Sep 20;13:giae058. doi: 10.1093/gigascience/giae058 (PMC11413801; doi:10.1093/gigascience/giae058)
Supplement: giae058_GIGA-D-23-00271_Revision_2 [file giae058_giga-d-23-00271_revision_2.pdf]

GigaScience

An interconnected data infrastructure to support large-scale rare disease research

--Manuscript Draft--

Manuscript Number:	GIGA-D-23-00271R2
Full Title:	An interconnected data infrastructure to support large-scale rare disease research
Article Type:	Research
Funding Information:	Horizon 2020 Framework Programme (779257) — Dr Sergi Beltran, Prof Holm Graessner<br>Horizon 2020 Framework Programme (305444) — Dr Sergi Beltran<br>Horizon 2020 Framework Programme (825575) — Prof Ana Rath, Dr Sergi Beltran<br>Instituto Nacional de Salud (PT13/0001/0044) — Dr Sergi Beltran<br>Instituto Nacional de Salud (PT17/0009/0019) — Dr Sergi Beltran<br>Horizon 2020 Framework Programme (825775) — Dr Thomas Keane<br>Nederlandse Organisatie voor Wetenschappelijk Onderzoek (VIDI 917.164.455) — Prof Morris A Swertz
Abstract:	The Solve-RD project brings together clinicians, scientists, and patient representatives from 51 institutes spanning 15 countries to collaborate on genetically diagnosing (“solving”) rare diseases (RDs). The project aims to significantly increase the diagnostic success rate by co-analysing data from thousands of RD cases, including phenotypes, pedigrees, exome/genome sequencing and multi-omics data. Here we report on the data infrastructure devised and created to support this co-analysis. This infrastructure enables users to store, find, connect, and analyse data and metadata in a collaborative manner. Pseudonymised phenotypic and raw experimental data are submitted to the RD-Connect Genome-Phenome Analysis Platform and processed through standardised pipelines. Resulting files and novel produced omics data are sent to the European Genome-phenome Archive, which adds unique file identifiers and provides long-term storage and controlled access services. MOLGENIS “RD3” and Café Variome “Discovery Nexus” connect data and metadata and offer discovery services, and secure cloud-based “Sandboxes” support multi-party data analysis. This succesfully deployed and useful infrastructure design provides a blueprint for other projects that need to analyse large amounts of heterogeneous data.
Corresponding Author:	Morris Swertz<br>UMCG: Universitair Medisch Centrum Groningen<br>Groningen, NETHERLANDS
Corresponding Author Secondary Information:	
Corresponding Author's Institution:	UMCG: Universitair Medisch Centrum Groningen
Corresponding Author's Secondary Institution:	
First Author:	Lennart F Johansson
First Author Secondary Information:	
Order of Authors:	Lennart F Johansson<br>Steve Laurie<br>Dylan Spalding<br>Spencer Gibson

Powered by Editorial Manager® and ProduXion Manager® from Aries Systems Corporation

	David Ruvolo
	Coline Thomas
	Davide Piscia
	Fernanda de Andrade
	Gerieke Been
	Marieke Bijlsma
	Han Brunner
	Sandi Cimerman
	Farid Yavari Dizjikan
	Kornelia Ellwanger
	Marcos Fernandez
	Mallory Freeberg
	Gert-Jan van de Geijn
	Roan Kanninga
	Vatsalya Maddi
	Mehdi Mehtarizadeh
	Pieter Neerincx
	Stephan Ossowski
	Ana Rath
	Dieuwke Roelofs-Prins
	Marloes Stok-Benjamins
	K Joeri van der Velde
	Colin Veal
	Gerben Van der Vries
	Marc Wadsley
	Gregory S Warren
	Birte Zurek
	Thomas Keane
	Sergi Beltran
	Holm Graessner
	Morris A Swertz
	Anthony J Brookes
**Order of Authors Secondary Information:**	
**Response to Reviewers:**	We thank the editor and both reviewers for the work put in to the review and the further comments. We believe the manuscript has benefitted a lot from the useful comments.<br><br>Reviewer reports:<br>Reviewer #1: I appreciate the addition of Supplement Figure S1 which helps clarifying the different steps of the workflow. The higher quality figures allowed me to assess their information. All my other comments have been addressed.<br><br>Thank you.

Powered by Editorial Manager® and ProduXion Manager® from Aries Systems Corporation

	Reviewer #2: Thank you to the authors for careful consideration of the comments made in the previous review.<br><br>I think the paper has benefitted substantially from the changes that have been made.<br><br>Overall I'm happy with the paper and only have some minor suggestions below.<br><br>I still think that the paper is probably a bit too long, so it would benefit from some further editing to make it a bit more concise, however, I will leave that to the authors and editors to decide on the appropriate length.<br><br>Here are my minor comments. I don't think another round of reviewing is needed if these items can be considered by the authors:<br><br>We have corrected the typo's<br><br>page 10 "Consortium Code Of Conduct" previously this was called "project code of conduct" maybe standardise on the name?<br><br>We now have also called it the project Code of Conduct on page 10.<br><br>page 11 "has also lit a beacon" I understand that "lighting a beacon" is the terminology used within the Beacon community, but I find it to be less helpful for those outside the community. Many readers will not know that this is special jargon in the community and it will be confusing. By all means, use the jargon, but it would be useful to have its meaning also spelled out for non-specialists.<br>We have changed "lit a beacon" to "created a beacon"<br><br>page 16 "the operational support teams are actively synergising with related projects" I'm not really sure what this means specifically, maybe it could be stated more clearly?<br>We have changed "synergising" to "working together"<br><br>page 17 "pseudonymised" perhaps you can elaborate on specifically what was done to pseudo-anonymise the data?<br>The pseudonimization process here means that only the submitter knows the full identifiers of a participant submitted to RD-Connect GPAP. Essentially the pseudonimization entails the assignment of a P-ID to each participant.<br>We have now added the sentence "The relation between P-ID and full personal identifiers is known only to the submitter." To the section on p17/18.
**Additional Information:**	
**Question**	**Response**
Are you submitting this manuscript to a special series or article collection?	No
**Experimental design and statistics**<br><br>Full details of the experimental design and statistical methods used should be given in the Methods section, as detailed in our Minimum Standards Reporting Checklist. Information essential to interpreting the data presented should be made available in the figure legends.<br><br>Have you included all the information	Yes

requested in your manuscript?	
**Resources**<br><br>A description of all resources used, including antibodies, cell lines, animals and software tools, with enough information to allow them to be uniquely identified, should be included in the Methods section. Authors are strongly encouraged to cite Research Resource Identifiers (RRIDs) for antibodies, model organisms and tools, where possible.<br><br>Have you included the information requested as detailed in our Minimum Standards Reporting Checklist?	Yes
**Availability of data and materials**<br><br>All datasets and code on which the conclusions of the paper rely must be either included in your submission or deposited in publicly available repositories (where available and ethically appropriate), referencing such data using a unique identifier in the references and in the “Availability of Data and Materials” section of your manuscript.<br><br>Have you have met the above requirement as detailed in our Minimum Standards Reporting Checklist?	Yes

Manuscript

Click here to access/download;Manuscript;Manuscript_v4-with editor comments_corrected.docx

An interconnected data infrastructure to support large-scale rare disease research

Lennart F. Johansson[1,b], Steve Laurie[2], Dylan Spalding[3], Spencer Gibson[4], David Ruvolo[1], Coline Thomas[3], Davide Piscia[2], Fernanda de Andrade[1], Gerieke Been[1], Marieke Bijlsma[1], Han Brunner[5,6,7]. Sandi Cimerman[1], Farid Yavari Dizjikan[4], Kornelia Ellwanger[8], Marcos Fernandez[2], Mallory Freeberg[3], Gert-Jan van de Geijn[1], Roan Kanninga[1], Vatsalya Maddi[4], Mehdi Mehtarizadeh[4], Pieter Neerincx[1], Stephan Ossowski[8,13], Ana Rath[9], Dieuwke Roelofs-Prins[1], Marloes Stok-Benjamins[1], K. Joeri van der Velde[1], Colin Veal[4], Gerben van der Vries[1], Marc Wadsley[4], Gregory Warren[4], Birte Zurek[8], Thomas Keane[3], Holm Graessner[8,10], Solve-RD consortium*, Sergi Beltran[2,11,12], Morris A. Swertz[1,a,b] and Anthony J. Brookes[4,a]

[a] Shared last authors

[b] Corresponding author

*Full lists of all authors available at the end of this document: Solve-RD consortium

[1] University of Groningen, University Medical Centre Groningen, Groningen, Department of Genetics, Genomics Coordination Centre, The Netherlands

[2] CNAG-CRG, Centre for Genomic Regulation (CRG), The Barcelona Institute of Science and Technology, Barcelona, Spain

[3] European Bioinformatics Institute, European Molecular Biology Laboratory, Wellcome Genome Campus, Hinxton, Cambridge, UK

[4] Department of Genetics and Genome Biology, University of Leicester, Leicester, UK

[5] Department of Human Genetics, Radboud University Medical Center, Nijmegen, The Netherlands.

[6] Donders Institute for Brain, Cognition and Behaviour, Radboud University Medical Center, Nijmegen, The Netherlands.

[7] Department of Clinical Genetics, Maastricht University Medical Centre, Maastricht, the Netherlands.

[8] Institute of Medical Genetics and Applied Genomics, University of Tübingen, Tübingen, Germany

[9] INSERM, US- 14 Orphanet, Paris, France

[10] Centre for Rare Diseases, University of Tübingen, Tübingen, Germany

[11] Universitat Pompeu Fabra (UPF), Barcelona, Spain

[12] Departament de Genètica, Microbiologia i Estadística, Facultat de Biologia, Universitat de Barcelona (UB), Barcelona, Spain

[13] Institute for Bioinformatics and Medical Informatics (IBMI), University of Tübingen, Tübingen, Germany

Correspondence to m.a.swertz@gmail.com or l.johansson@umcg.nl

ORCID iDs:

Lennart F Johansson [0000-0002-4914-3737]; Steven Laurie [0000-0003-3913-5829]; Dylan Spalding [0000-0002-4285-2493]; Spencer Gibson [0000-0003-0768-1542]; David Ruvolo [0000-0002-5745-5298]; Coline Thomas [0000-0003-2253-1171]; Davide Piscia [0000-0002-0468-0408]; Fernanda de Andrade [0000-0003-4689-6319]; Gerieke Been [0009-0002-6667-5951]; Marieke Bijlsma; Han Brunner [0000-0001-9274-8865]; Sandi Cimerman [0009-0009-9918-2185]; Farid Yavari Dizjikan; Kornelia Ellwanger [0000-0003-4845-5795]; Marcos Fernandez [0000-0002-9968-3766]; Mallory Freeberg [0000-0003-2949-3921]; Gert-Jan van de Geijn [0000-0001-6161-0476]; Roan Kanninga; Vatsalya Maddi [0000-0002-6188-7384]; Mehdi Medtarizadeh; Pieter Neerincx [0000-0002-9800-8638]; Stephan Ossowski [0000-0002-7416-9568]; Ana Rath [0000-0003-4308-6337]; Dieuwke Roelofs-Prins; Marloes Stok-Benjamins [0000-0001-8534-378X]; K Joeri van der Velde [0000-0002-0934-8375]; Colin Veal [0000-0002-9840-2512]; Gerben van der Vries [0009-0000-1422-1435]; Marc Wadsley [0000-0002-7014-2595]; Gregory Warren; Birte Zurek [0000-0002-8200-7542]; Thomas Keane [0000-0001-7532-6898]; Holm Graessner [0000-0001-9803-7183]; Sergi Beltran [0000-0002-2810-3445]; Morris A Swertz [0000-0002-0979-3401]; Anthony J Brookes [0000-0001-8686-0017];

Abstract

The Solve-RD project brings together clinicians, scientists, and patient representatives from 51 institutes spanning 15 countries to collaborate on genetically diagnosing ("solving") rare diseases (RDs). The project aims to significantly increase the diagnostic success rate by co-analysing data from thousands of RD cases, including phenotypes, pedigrees, exome/genome sequencing and multi-omics data. Here we report on the data infrastructure devised and created to support this co-analysis. This infrastructure enables users to store, find, connect, and analyse data and metadata in a collaborative manner. Pseudonymised phenotypic and raw experimental data are submitted to the RD-Connect Genome-Phenome Analysis Platform and processed through standardised pipelines. Resulting files and novel produced omics data are sent to the European Genome-phenome Archive, which adds unique file identifiers and provides long-term storage and controlled access services. MOLGENIS "RD3" and Café Variome "Discovery Nexus" connect data and metadata and offer discovery services, and secure cloud-based "Sandboxes" support multi-party data analysis. This succesfully deployed and useful infrastructure design provides a blueprint for other projects that need to analyse large amounts of heterogeneous data.

Keywords

Rare disease, genetics, bioinformatics, computational biology, fair data, infrastructure

BACKGROUND

Solve-RD is a Horizon 2020 supported EU flagship project that brings together >300 clinicians, scientists, and patient representatives from 51 institutes across 15 countries [1]. Solve-RD is built upon a core group of four European Reference Networks (ERNs) ERN-ITHACA, ERN-RND, ERN-Euro NMD and ERN-GENTURIS) and two associated ERNs (ERN RITA and ERN-EpiCARE), and the Spanish and Italian national Undiagnosed Diseases Programs, which annually see more than 270,000 rare disease (RD) patients with varying pathologies. The main ambition of Solve-RD is to solve unsolved RD cases for which a molecular cause is not yet known. This is achieved through an innovative clinical research environment that introduces novel ways to organise expertise and data. Two major approaches are being pursued: (i) massive data reanalysis of >19,000 experiments (various forms of genetic testing) from individuals affected by a rare condition and their unaffected family members and (ii) combined analysis of diverse types of newly-generated data, ('novel' omics data).

For the data reanalysis, ERN partners contributed pseudonymised data (phenotypic data, pedigree information, exome sequencing (ES) data / genome sequencing (GS) data and associated metadata) for individuals affected by a RD who remained genetically undiagnosed after ES or GS. Data were submitted via the RD-Connect Genome-Phenome Analysis Platform (GPAP) [2]. In addition, novel omics data (short- and long-read GS, short and long-read RNA-sequencing, epigenomics, metabolomics, deep-ES, and optical genomic mapping) are being generated by different service providers for cohorts defined by the Data Interpretation Task Forces (DATF) from the four core collaborating ERNs [1]. Sample submitters from the ERNs upload their pseudonymised phenotypic and pedigree information in the RD-Connect GPAP PhenoStore module.

From there, Phenopackets and pedigree descriptions in PLINK PED format are exported and submitted to the European Genome-phenome Archive (EGA). When novel omics data is generated, the service providers upload it directly to the EGA together with a manifest that links it to the corresponding individual. With such an amount of data to be analysed in a collaborative manner, downloading and analysing on local compute facilities is not feasible for all centers. Therefore, also centralised analysis facilities were desired.

All this clearly highlights the project's need for a supporting data infrastructure. In particular because diverse demographic, phenotypic and multi-omics data needs to be securely submitted by a large number of clinical centers and other data providers, over a multi-year period. The quality of data and the relationships between data and files need to be captured to enable optimal use of the available data. Furthermore, to enable researchers from different centers to work together on the same dataset an accessible cloud infrastructure is required for all researchers.

To enable reproducibility of analyses we organised the datasets in freezes of fixed sets of participants, that were updated with patches containing new information that became available over time. This information is captured within a MOLGENIS database [3; 4] and supplemented with an advanced discovery layer based on Café Variome [5] to enable identification of cases or sets of cases (virtual cohorts) based on a wide array of filters, including phenotypic or genotypic similarity metrics and federation with other RD data and sample resources. In addition, appropriate metadata (e.g. file checksum) is collected to ensure that file integrity is maintained during transfer between research centres. This allows researchers to select samples of interest, for instance all affected individuals with a specific phenotype, and collect the associated files at their preferred analysis location.

Similar discoverability features are available through the RD-Connect GPAP cohorts application. Furthermore, the RD-Connect GPAP is connected to MatchMaker Exchange [19] and the Network of Beacons [6], enabling bidirectional patient matchmaking queries to similar resources around the world.

The Solve-RD project infrastructure has been constructed by leveraging existing data platforms, tools and standards wherever possible, and by creating new tailored implementations where necessary, assembled into an interconnected infrastructure. We have operated on the core principle that we will reuse, enhance and deploy existing solutions (for core analytics support, databasing, data discovery and data sharing) wherever possible, according to FAIR data principles [7]. This paper describes the current state of the infrastructure which is fully operational, and indicates how we are further improving and extending its capabilities to ensure its future relevance and wider utility. We believe the resulting infrastructure could provide a template which future large scale RD analysis projects can start from. Most of the components are tailored for RD research, however, the general design and some components of the infrastructure can also be of use for groups focusing on other topics.

RESULTS

The data infrastructure we have developed for Solve-RD facilitates submission of input data, a common approach to processing and archiving, collaborative data analysis, and sophisticated data discovery. The overall design and data flow is summarised in Figure 1.

DATA SUBMISSION AND PROCESSING

Experimental metadata are first submitted to the RD-Connect GPAP, and corresponding phenotypic data submitted to the GPAP PhenoStore, where patient, phenotypic and family information are stored. Associated omics and pedigree data files then follow one of two paths, as described in the methods. Pre-existing sequencing data are submitted to the RD-Connect GPAP where they are processed through the RD-Connect standard analysis pipeline to homogenise results and facilitate systematic analysis, interpretation, and comparisons [8]. The raw and proceed data can then be downloaded by project partners and processed with a secondary tool e.g. for the identification of copy number variants or short tandem repeat variants. After processing, raw data, alignments and detected genetic variants are submitted to the GPAP analysis platform and forwarded to the EGA to be archived. Newly-generated novel omics data are archived directly to the EGA. As described in our Methods section, the standard file formats used within our workflow, led to easy hand-off capabilities between the different components.

Long-term storage

At the EGA a unique identifier (UID) is added to each individual file and data are made available for download. A manifest file (supplementary table S1) with metadata provides background information on the origins of the files to aid in future data interpretation. In parallel the Solve-RD Rare Disease Data about Data (RD3) database collects data and metadata on subjects, samples, experiments and files from these sources, and makes this available for discovery using the Discovery Nexus service, both described below.

Standard Processing of reanalysis samples

Sequencing data originating from 43 different research centres was submitted together with a common set of required metadata for each participant and associated experiment. Solve-RD includes fully reanalysed ES or GS data from 22,326 participants (data freezes 1-3) for whom routine diagnostic procedures failed to achieve a molecular diagnosis. Furthermore, novel omics data from 5,184 participants (2,280 SR-GS, 510 LR-GS, 634 SR-RNAseq, 80 LR-RNAseq, 480 Epigenomics, 930 deep-ES, 270 Metabolomics) has been newly generated and incorporated. All of these data will be fully processed within the project [1; Laurie et al., unpublished observations] Solve-RD has archived over 750,000 files of primary and processed data at EGA totalling 818 terabytes. Impressively, this represents nearly 5% of all data archived at EGA, the second largest project at EGA to-date. The data held by the EGA will be fully available, under controlled access, to the wider RD community, and the ES/GS variant data is available to browse and analyse by any registered RD-Connect GPAP user.

Freezes and patches

Data are structured into freezes and patches [1]. The Solve-RD project has generated three large freezes that consist of reanalysis data from subjects and experiments that have been submitted prior to one of three deadlines, meaning that each freeze consists of a fixed number of experiments and participants. The submission closing date for the first freeze was 30 September 2019 and it included data from 8,275 participants. The second and third freeze closed on 30 September 2020, and included data from 3,192 participants. The third freeze closed on 30 September 2021 and included data from

10,516 participants. Changes in data or metadata for these subjects are captured in patches, leaving the original dataset on which analyses have been performed intact, making reanalysis possible. In addition, two data freezes for the novel omics data have been generated. For a small number of participants there were unintended duplications of datasets; a few cases had to be withdrawn from the collection for different reasons. To allow for data changes post-submission (e.g. addition of new phenotypic information or correction of errors), serial patches were introduced for each freeze. Patched files were released with a date inserted between the preserved filename and its file type extension (i.e., FILENAME.YYYY-MM-DD.extension). For each original freeze or subsequent patch all data was included in a uniquely identifiable EGA dataset (EGAD).

DATA ANALYSIS

Data analysis was performed by data analysis task force (DATF) teams and interpretation of variants was done by data interpretation task force (DITF) teams. DATF activities were divided over several working groups [1] tackling ES and GS reanalysis and processing the newly generated 'novel omics' data. Only approved researchers who had signed the project code of conduct (Supplementary information 1) could access the data. Solve-RD partners can analyse data through three main approaches: the RD-Connect GPAP, a cloud-based 'Sandbox' and authorised local clusters.

While a wide range of analyses can be performed using the RD-Connect GPAP user interfaces (as described in the methods section) new analysis methods to find or interpret new variants and solve cases are continuously being developed. Moreover, for the novel omics data, analysis protocols are not yet standardised and needed to be developed by

Solve-RD partners. We therefore needed an extensive analysis infrastructure to enable project analyses. A data request and download option was provided for partners that had their own substantial local compute facilities after approval of the project steering committee.

Data management within analysis Sandbox

To support groups that did not have large compute and storage capacity, and also to enable multi-centre collaborative analyses, a centralised analysis ‘cloud’ Sandbox was established. It supports existing and new research methods and allows collection and sharing of project results. The Sandbox approach provides a central analysis environment for bioinformaticians to collaborate and to use and develop new methods freely. Via the Sandbox, DATF and DITF working groups performed pilot studies using newly devised tools to assess their added value, before undertaking an analysis of full datasets. The Sandbox functions as “Virtual/Trusted Research Environment” (VRE/TRE) or ‘Safe Haven’, providing access to data for analysis while protecting patient confidentiality supported by trained staff and agreed processes [9]. Before users could access any Sandbox content, a project Code Of Conduct had to be signed and approved.

The Solve-RD Sandbox provides a Linux-based high performance compute (HPC) environment suited to bioinformaticians. To provide failover, we have deployed the Sandbox on two separate clouds. The Sandbox supports large-scale data storage organised as a high-performance temporary (tmp) section and a stable but slower back-upped permanent (prm) folder. The tmp folder supports data analysis and so has a free

structure for individual users to manage. The prm folder has a fixed structure that was identical at both Solve-RD Sandboxes.

Within each of the two VREs the tmp folder includes a single master folder containing original freeze files as well as patched files. For each freeze and patch a folder exists that carries symlinks to the files included in the specific patch release, typically a mix with the majority of files included in the previous patch and some new changed files. Because of limited storage space not all files from the project could be simultaneously held in the Sandbox. Therefore, larger files were omitted and reloaded as and when needed. In addition to these folders an ega-fuse-client folder was present in the prm folder, giving direct access to the Solve-RD datasets archived at the EGA. This enables the large files to be accessed from within the VRE even though no local copy was present.

To provide access to analysis results, a dedicated directory was created for each DATF working group. To store their analysis results, each DATF working group appointed a data manager who was allowed to copy, move and remove data to and from the prm folders on the VREs (automatically synchronised between the two VRE instances). The folders were structured such that data sharing was optimally facilitated (Figure 2).

DATA DISCOVERY

Many diverse data types and files exist within the Solve-RD project (multi-omics, variant interpretation, phenotyping, demographics, etc). These are stored in different places and in different formats. The totality of metadata can be navigated via the RD3 database, based on the MOLGENIS technology [3;4]. Via RD3 and the advanced discovery layer

‘Discovery Nexus’, DATF bioinformaticians can find samples and data of interest. To do this they formulate queries that identify file identifiers (EGAF) for relevant data stored in EGA, to then access these data in the sandbox, in GPAP, or in their local cluster.

Additional data discovery functionalities are provided by the RD-Connect GPAP, as described in [2]. These consist of an internal “search across all” functionality, allowing users to search for specific types of variant in candidate genes of interest across all experiments. This can be further refined using the “cohorts” application which allows identification of affected individuals with similar phenotypes within the RD-Connect GPAP, including data not submitted as part of Solve-RD. The RD-Connect GPAP is also an active node in the international MatchMaker Exchange network, facilitating patient matchmaking worldwide [10], and has also created a beacon within the GA4GH Beacon Network [11].

RD3 - tracking files and metadata

Direct data navigation is supported by the ‘rare disease data about data’ (RD3) system. This MOLGENIS database provides a complete listing of all patients/participants, samples, experiments and data files in Solve-RD, including EGA UIDs. The data model of the Solve-RD project describing how data is organized is summarised in Figure 3.

Some relationships are direct, such as the subject-sample relation (a sample is derived from a subject), whereas others are not so obvious and need to be discovered. RD3 is tightly integrated with Discovery Nexus, which also leverages useful extractions of various data files (e.g. extant variants, their frequency, host gene, mutation type, etc).

Following a successful Discovery Nexus search, suitably permissioned users can click through to RD3 directly to access the discovered data files.

Discovery Nexus

Discovery Nexus supports data discovery via a range of approaches that help users initially establish the existence and location (rather than the substance) of data within the system. The interface provides filtering options by which users can distil a comprehensive overview of selected datasets that might be of value for their intended purpose. Querying by multiple data values is possible, driven by ontologies and ontology cross-mappings. Searches can look for identity or semantic similarity to an entered term, or any combination of terms, and even extends to bridging between concepts (e.g., searches by biochemical pathway leverage knowledge of which genes are in each pathway). It also supports the GA4GH standard Beacon-2 API [6] for wider interoperability.

Discussion

To enable numerous researchers and clinicians to work together in parallel on a large dataset in Solve-RD, it was essential to establish a good data infrastructure that adheres to FAIR principles (See Box 1). The solution we created includes access policies and procedures, including the code of conduct (supplementary information 1), a network of databases, HPC clusters, long-term storage capabilities, federated discovery services, tools and pipelines to provide the project with the ability to solve many RD cases that had not been solved using conventional strategies. The infrastructure can be used starting from three main goals: data submission and processing, data analysis and data

discovery, as described in the results and methods. For each of these goals the most typical workflow is shown in supplementary figure S1. Depending on the user, different parts of the infrastructure are used. Typically, clinicians will submit samples, whereas the researchers, split between the DITF and DATF will set up cohorts of patients with similar phenotypes, find different types of genetic variants through various analyses and zoom in on possible causal genetic variants. Using this infrastructure, the Solve-RD project has already made >500 new diagnoses [Laurie et al., unpublished observations], and many analyses powered by novel omics data are still ongoing.

The two parallel tracks, reanalysis of existing ES or GS data and novel omics data analysis, each created distinct challenges. One of the main challenges of the exome reanalysis stemmed from the heterogeneity of the submitted data. Cases were provided by institutions all around Europe and exomes were enriched using various designs and versions, and sequenced using different short-read platforms, each of which will result in different biases. In addition, analyses prior to submission to Solve-RD had been performed using a range of different alignment and variant-calling algorithms. To facilitate data integration, the Solve-RD project reanalysed primary sequence data from the earliest possible point, using a standardised workflow, thereby eliminating bioinformatic-related differences and providing a coherent set of files for each of the experiments submitted. In parallel, the RD-Connect GPAP processed participant metadata and pedigree information and exported these in standard file formats. This provided reusable and interoperable data enabling downstream analysis via the RD-Connect GPAP, the project Sandboxes and local clusters.

Regarding novel-omics the main challenges from the perspective of the infrastructure were the different types of files produced and differences in accompanying metadata, which required a custom-made database format to capture this data.

Data FAIRness was enhanced by placing the data within the EGA data archive for long-term storage, request and access. To maximise user convenience, single-sign-on capability was provided across different components supporting a single goal, such as RD3 and Discovery Nexus, or between the Sandboxes and EGA via the filesystem in userspace (FUSE) client, as described in the methods. We also developed innovative methods to make data findable before and after data access is granted, using Discovery Nexus for preliminary searches (interoperable with GA4GH Beacon technology), and the RD3 database for full dataset navigation. Once the Solve-RD funding period is over, this same service will enable ERN data owners to advertise their data to researchers outside the project without directly releasing data too liberally or before access requests are reviewed and data sharing agreements set up. The data discovery service will also provide potential users with sufficient insight into the nature of available datasets to be confident that it is worth investing effort to request and analyse the data. The Solve-RD omics data (i.e. pre-existing unsolved exomes and genomes as well as omics data generated within the project) are archived in the European Genome-phenome Archive (EGA) (Supplementary information S2) and will be made available to other rare disease researchers via a controlled access mechanism, governed by the Solve-RD Data Access Committee (DAC). The DAC consists of one representative per ERN that contributed data and/or samples to Solve-RD as well as a patient representative. Researchers who would like to access a specific Solve-RD dataset need to request access from the Solve-

RD DAC. To do this they have to fill and sign the Solve-RD Data Access Agreement (DAA) (Supplementary information S3) [12], and send it to the DAC office. The DAA lays out the terms under which access to Solve-RD data (including sequence and genotype data, other omics data, phenotypic data and pedigree information) is being granted.

Within projects such as Solve-RD, concrete analyses are often conceived after the collection of data. This reflects the continuous expansion of associated knowledge and support tools. To facilitate this, we emphasised structured collection of rich metadata, thereby making the available data unambiguous in terms of its scope, quality, provenance and location. RD3 was used to organise and provision these metadata, following FAIRGenomes guidelines [13]. In addition, the RD-Connect GPAP co-hosts sections of the metadata relevant to their content, and this metadata also allows cohort-building via both Discovery Nexus and the RD-Connect GPAP.

In conclusion, Solve-RD has devised, implemented, and validated an infrastructure for bringing together a set of reusable tools and best practices. As Solve-RD partners continue to use the infrastructure to perform many multi-omics analyses, the operational support teams are actively working together with related projects, ensuring sustainability and further development of the different infrastructure components. For example, some of the components are being deployed and expanded in European projects such as the European Joint Programme on Rare Diseases (EJP-RD, https://www.ejprarediseases.org), the EU Genome Data Infrastructure project (GDI, https://gdi.onemilliongenomes.eu/), and national initiatives such as the Dutch FAIR genomes/Health-RI genomics project [14]. Ongoing projects, such as GDI and the forthcoming ERDERA [15] support sustainability and future developments of the

components for the future. Hence, the infrastructure described in this paper can be used as a blueprint for future multi-omics data (re)analysis projects and data hubs.

Methods

The Solve-RD infrastructure consists of various interconnected parts, each playing a role in different workflows needed required by the project (supplementary figure S1). The components described in the sections below are listed in table 1.

INSERT TABLE 1 HERE

DATA SUBMISSION AND PROCESSING

Many types of data were provided by the ERNs or newly generated within the Solve-RD project, including demographic and phenotypic data of participants and metadata on samples, experiments and files. Pre-existing sequencing data are submitted to the RD-Connect GPAP as FASTQ [16], BAM [17], or CRAM [18] files via a RedIris Aspera server. Specifically, ES and GS reanalysis data and metadata was provided by partners of six different ERNs: ERN-ITHACA, ERN-RND, ERN-Euro NMD, ERN-GENTURIS, ERN RITA, ERN-EpiCARE. For novel omics analysis, various other file types and concomitant metadata were produced.

Raw ES and GS read data for reanalysis, together with accompanying metadata and deep phenotypic descriptions of affected individuals were submitted by Solve-RD partners to the RD-Connect GPAP (GPAP). Alignment and short variant calling was undertaken for all experiments using anidentical variant calling workflow [8], in order to

minimise bioinformatics induced artefacts. All identified SNVs and InDels were made immediately available to Solve-RD collaborators for analysis in the GPAP Genomics module. Subsequently the raw data, and processed data in the form of BAM/CRAM and gVCF files were transferred to the European Genome-phenome Archive (EGA) for longer-term archival and redistribution to other Solve-RD partners.

The RD-connect Genome-Phenome Analysis Platform (GPAP) was used for collation of all phenotypic data, and standardised processing of all short-read ES and GS data submitted to Solve-RD. Data collation was undertaken as described in Laurie et al., 2022 [2].

Briefly, in the first step pseudonymised phenotypic descriptions of all affected individuals were uploaded to the RD-Connect GPAP PhenoStore module, using HPO, OMIM and Orphanet terms to generate a detailed phenotypic description, together with a family tree linking individuals. Each individual receives a unique participant ID (P-ID) and for accompanying experiments E-IDs (experiment IDs) were created. The relation between P-ID and full personal identifiers is known only to the submitter. In the second step, metadata describing the raw sequencing data to be submitted for reanalysis and linking it to the individual's phenotypic record, is uploaded to the GPAP Data Management module. Finally, the raw sequencing data itself is transferred using a robust, high-speed Aspera data transfer service provided by RedIris, the Spanish academic and research network [19]. Once submission is complete, the data is automatically ingested and processed by the automated standard analysis pipeline.

Standard analysis pipeline

For joint data analysis, it is important that technical differences between experiments are minimised. Therefore, using the CNAG-CRG local HPC resources, all short-read ES and GS data submitted to Solve-RD were reprocessed using an identical standardised variant calling pipeline as described in Laurie et al., 2016 [8].

Data sources for pre-existing and new data

For reanalysis of ES/GS, novel omics short read (SR)-GS, and deep-ES data, the starting point for reanalysis was the associated FASTQ files. When BAM or CRAM files were submitted, these were first transformed back to FASTQ. Using the standard analysis pipeline (Figure 1), data were processed in a standardised manner as described above, producing a single BAM and 25 g.VCF files (autosomes, X, Y and MT), accompanied by .BAI and .TBI index files, respectively. Phenotypic information was exported from GPAP in Phenopacket format and pedigrees in PED file format. LR-GS files and RNA-sequencing data were stored in BAM format. Data analysis produced output of various file formats, depending on the tools used for analysis.

Interoperability

To maximise interoperability for tool integration and reuse beyond Solve-RD and to overcome language barriers, we use widely adopted and machine-readable international and community standards and ontologies whenever possible. Within PhenoStore, deep phenotypic descriptions are recorded using Human Phenotype Ontology [20], Orphanet Rare Disease Ontology (ORDO) [21] and the Online Mendelian Inheritance in Man (OMIM) [22] terminology. Phenotypic records can be exported using the GA4GH approved Phenopacket format [23], and family trees in PLINK PED format [24; 25].

Genomic alignments are stored and transferred (e.g., to the EGA) in GA4GH approved BAM, CRAM formats [16;17;26]. Variants are stored in GVCF format [27]. Biological annotations, available in the Data Analysis module, are provided by Ensembl VEP [28] and supplemented with data from other genomics community resources such as ClinVar (RRID:SCR_006169) [29], gnomAD (RRID:SCR_014964) [30], and PanelApp [31]. Data discovery and sharing is achieved through the implementation of GA4GH Beacon-V2 [6], and MME APIs [10]. Partner involvements in other initiatives also guided our work regarding other standardisation strategies, not least B1MG, GA4GH, FAIR genomes [13], ELIXIR, BBMRI and EJP-RD.

EGA long-term data archiving and access

The European Genome-phenome Archive (EGA) [32] is a service for permanent archiving and sharing of identifiable genetic and phenotypic data [33;34]. Data archived at the EGA ensures long term availability, interoperability, and identifiability during projects and beyond. The primary objects in the EGA data model are studies, datasets, and files (raw and processed). Each archived file is assigned an EGA accession (EGAF) functioning as a UID. Moreover, each file can be part of one or more datasets, each with its own accession number. After data are successfully archived and released, the EGA provides access to the data only upon approval by the associated DAC for specified individuals. Datasets can be accessed using the PyEGA3 streaming client [35] and a filesystem in userspace (FUSE) client [36].

To ensure data are FAIR, metadata are uploaded to EGA alongside data files. These metadata take the form of manifest files (supplementary table S1) which contain many

attributes describing the data, for example what library preparation and sequencing strategy was followed, what type of data analysis was done including which reference genome was used, and minimal public information about the study subjects. Manifest files are converted to the EGA XML standard for representing metadata before being permanently archived. To guarantee data security and preservation of data integrity during file transfer and archival at EGA, data files are submitted to EGA in an encrypted format and file checksums are compared at different points of the submission process. For example, encrypted file checksums are compared before and after upload to the EGA to ensure that the file was not corrupted during transfer. After being re-encrypted at EGA with a symmetric key and stored in the permanent archive, one final checksum check is performed to ensure integrity of the permanently archived, encrypted file.

DATA ANALYSIS

RD-Connect GPAP

The RD-Connect GPAP allows users to perform variant analysis to identify potential disease-causing variants in a single proband or any family structure and allows user-defined queries across a cohort of affected individuals. These capabilities are provided via a user-friendly interface suitable for clinicians, genome scientists and bioinformatics researchers.

A large variety of filters can be applied in order to identify known pathogenic variants, e.g. described in ClinVar, or prioritise variants that are potentially pathogenic for further investigation [2]. Furthermore, variants can be visualised in remotely hosted native BAM

files on-the-fly, directly within the GPAP, through implementation of the GA4GH htsget streaming protocol and a client-side Integrative Genomics Viewer instance [37].

Analysis can be undertaken in two different ways, either interactively via a graphical user interface (GUI) or automated via a Python-based API. The interactive approach is ideal for analysing individual families and applying different filter strategies. For processing large numbers of experiments, as undertaken in Solve-RD, programmatic batch analysis can be undertaken as described previously [38].

Intra-GPAP case-matching is possible via an instance of the GA4GH MME API [39] and by searching across cohort functionalities. External case matching can be achieved through the global MME API [10], and single variants can be found via the Beacon-V1 API [11].

Sandboxes for bespoke bioinformatics analyses

Bioinformatics methods often require a Linux command-line environment and extensive computing and storage capabilities. In line with this, we implemented two Sandboxes as Linux-based HPC clusters that can be remotely accessed and act as a VRE/TRE. To enable reproducibility and reusability (i.e. in future projects) these Sandboxes are implemented as a ‘cloud’ service that can be automatically deployed at different cloud providers using the same playbook [40], using OpenStack for virtualisation of Linux CentOS7 [41] with Spacewalk [42] for package distribution and management and using the LMOD module system [43] and Easybuild [44] to reproducibly install bioinformatics tools.

Because HPC systems typically need large maintenance windows where the service is offline, we have two separate Sandbox installations at different locations to prevent a single point of failure and ensure continuous operations to the partners: one at EMBASSY [45;46], hosted by the EMBL European Bioinformatics Institute (EMBL-EBI), which has close connections to the EGA, and one at the University of Groningen Centre for Information Technology [47;48] attached to the University Medical Centre Groningen. The EMBASSY VRE is only accessible by members of the Solve-RD project, while the UMCG VRE is a larger facility shared with other projects beyond Solve-RD. A dedicated Solve-RD group is present in the UMCG VRE with access restricted to Solve-RD members only. The EMBASSY VRE has 40 Tb of storage and 12 compute nodes with 14 cores/node and 56072 Mb RAM/node. The UMCG VRE [49] has shared storage with other projects, with 200 Tb reserved for the Solve-RD project and a total of 10 compute nodes available with 22 cores/node and 205490 Mb RAM/node.

Access to analysis results

Both clusters use internal networks that are not directly accessible from the internet. Access is possible via dedicated jumphosts, security hardened machines not involved in any data storage or processing. Using asymmetric cryptography via a private-public key pair [50], users can login to the jumphost to be directly redirected to the main HPC cluster. To allow for data access for non-bioinformaticians, we created an SFTP transfer server that could be accessed using a graphical user interface such as WinSCP [51], MobaXTerm [52] or Cyberduck [53] via a private-public keypair without the extra security of a jumphost.

DATA DISCOVERY

MOLGENIS RD3

To manage metadata on subjects, samples, experiments and files of ES reanalysis and novel omics, we used the MOLGENIS RD3 database. A specific Solve-RD instance of this was created [54], accessible via a web-interface [55]. In this database, metadata (e.g. file accession numbers) and data (e.g. average coverage for ES targets) are collected for all Solve-RD subjects and the associated samples, experiments and files.

Content includes information on how samples were collected and the subjects they came from, as well as the analyses that were performed and the location of the files generated. RD3 acts as a hub for GPAP data on Solve-RD participants, data provided by the EGA, files located in the Sandbox, and metadata required for the Discovery Nexus tool. Using portal tables, relevant data and metadata are imported into RD3 using a manifest file provided by the EGA.

RD3 was built in MOLGENIS [3,4], an open-source database platform for storing, managing, analysing, and sharing data. Approved users can log in using a local login or through FusionAuth [56] . All the relevant metadata for the research is collected within the Solve-RD RD3. The core structure of RD3 consists of several tables matching the different types of information that should be selected (Figure 3). ES reanalysis data was imported into RD3 using a system of freezes and patches as described in the results. Each of these sections has the same format.

The subjects table contains information on the participants as collected in GPAP PhenoStore, imported via phenopackets and PED files archived at the EGA. Subjects are identified based on their P-ID. For each subject the P-IDs of the parents are given if they were included in the project, as is the family number to identify all subjects who are part of the same family. Furthermore, the subject's sex and a disease name or the phenotypes known to be present (or absent) are listed. For each subject, it is recorded if they are considered to be affected by a condition or not (e.g. a child is affected and both parents are unaffected by a condition). In addition, information is stored on the case submitter, e.g. if they are allowed to be recontacted in case of incidental findings or if the case is retracted. Finally, the subjects table shows if the sample is solved. Because this information is updated in the GPAP PhenoStore, a connection between the two programs allows the solved status to be updated daily.

Zero or more samples may be derived from each subject. Sample metadata is collected in the samples table. Each sample is given a sample-ID (S-ID) for unique identification. Per S-ID, the P-ID of the subject from which it is derived is shown as well as the tissue type (e.g. whole blood) and other sample specifications.

Zero or more experiments can be performed on each sample (e.g. ES on DNA isolated from the sample). Information on these experiments is collected in the experiments table (see Figure 4). Each type of experiment has its own specific lay-out. For ES the enrichment kit used is captured as is the sample preparation method. The metrics "% of the target covered >20x" and "average target coverage" are also collected.

For each family, subject and experiment files are archived at the EGA. RD3 captures this information in the files table. Here, for each file, the path in the Sandbox and the VRE ega-fuse-client within the dataset are given with its checksum information enabling a sanity check on copies of this file. Information is recorded about the filetype, the experiment it belongs to and the EGA accession number.

Discovery Nexus

RD3 is seamlessly integrated with Discovery Nexus using a single sign-on option based on the open ID connect protocol (OIDC, implemented using FusionAuth), which is compatible with the life sciences AAI, previously known as ELIXIR AAI [57], which we plan to implement in the future. The latter will enable users to sign in using their institute sign in, which increases security and GDPR compliance and ensures removal when contracts terminate.

Discovery Nexus is a parallel component to RD3 that provides advanced and more powerful capabilities for quickly and deeply searching Solve-RD data stored in different locations and formats. Built on Café Variome [5], Discovery Nexus abstracts direct database-style queries to concept-based queries, for example, phenotypes and diseases are based on common ontologies that Discovery Nexus dynamically maps to ontologies and hierarchies within ontologies used in the underlying subject phenotyping. This is also extended to querying using semantic similarity between and across ontologies. This abstraction allows Discovery Nexus to represent searches in an intuitive query builder interface focussed on elements that make queries based on demographics, phenotypes, diseases, variants, biochemical pathways, mutation characteristics, solved-or-not status,

and data availability (Figure 5). This separation of query from database language also provides protection to subjects and studies identification as the actual data is not queried or represented in the query or results. For example, variants are not directly queried in Discovery Nexus; instead, the query interface allows searches for types of variant mutations in genes or gene families.

Handoff from Discovery Nexus to RD3 to get data

Discovery Nexus and RD3 operate under a federated single sign-on for authentication using the industry standard OIDC provided by RD3, with only users authorised by Solve-RD able to access either application. This allows the two parallel systems to interoperate seamlessly, with a handoff facility allowing search results in Discovery Nexus to be pre-populated in RD3, so that the user access information about the underlying data without logging in again.

Figures

Fig. 1: rare disease analysis infrastructure overview. GS, genome sequencing. ES, exome sequencing. LR-GS, long-read genome sequencing. SR-GS, short-read genome sequencing. LR-RNAseq, long-read RNA-sequencing. SR-RNAseq, short-read RNA-sequencing. deep-ES, Deep sequencing ES. EGA, European Genome-phenome Archive. ERN, European Reference Network. GPAP, Genome-phenome analysis platform. UI, user interface. The Solve-RD dataset is also discoverable through the participation of the RD-Connect GPAP in Matchmaker exchange and the Beacon Network.

Fig. 2: Sandbox folder structure. Data is organised by the data analysis working groups (DATF WG) in either folders per European Reference Network (ERN) or a common folder (for data intended for all ERNs). Additionally, large files that should be kept but not shared are stored in a 'Sandbox only' folder. All data to be shared with the ERNs is linked to an sftp folder with a subfolder per ERN accessible via SFTP access

protocol. Thin arrows indicate links between specific subfolders. These folders are further synchronised to two folders: DATF and DITF (data interpretation task force), each with the same information (indicated by the thick arrow). The DATF folder has the same structure as the initial sftp folder (a folder for each DATF WG with subfolders per ERN). The DITF folder has the converse structure (a folder for each DITF ERN with subfolders per WG). This structure makes it easy for both DATF and DITF to browse the data (e.g. all CNV data or all data from ERN-ITHACA).

Fig 3: Data and metadata relations within Solve-RD. Arrows indicate the 'derived from' direction, e.g. Sample DNA00001 is derived from Subject P00001. We distinguish four main data/metadata types: subject, sample, experiments and files, with each derived from the former. This figure is actually a simplification as data is further organised in data releases we call 'freezes' and can be used in different combinations as 'analyses'.

Fig. 4: Solve-RD RD3 LabInfo screen showing a subset of the Freeze1 experiment data. On the left entries are filtered on patch 'Original data' and columns are filtered on interest. In the current view, the experimentID is connected to the sample on which the experiment was performed. In addition, information on the experiment is shown. For these samples genomic data was the input for exome sequencing experiments on which various different enrichment kits were used. For most of the samples statistics on the average target coverage (MeanCov) and number of bases covered by at least 20 sequencing reads (C20) was available. If a subject was retracted from the project, all metadata except identifiers were removed from the database and the experiment was labelled as retracted.

Fig. 5A: Discovery Nexus query interface.

This interface supports querying by any combination of various demographic and inheritance (Subject Filters), phenotypes (HPO Query Builder), diseases (ORDO Query Builders) or suspected variant filters (Variant Filter). In the HPO Query Builder typing any part of an HPO phenotype term or code creates a visible list of relevant items to select from, whereupon they are transferred into the adjacent panel to form part of the query. Phenotype matching can specify matching on identical terms only (exact) or recover similar terms (based on a precomputed matrix of relationship scores and the position of the slider). The minimum number of matching terms can also be specified, creating an "OR" query, settings above the

minimum creates a query that returns results that match at least the specified number of terms in any combination. HPO queries can also be instructed to interrogate phenotype data stored as ORDO terms. Matching of HPO to ORDO terms (in the ORDO Query Builder) is controlled by the HPO pairwise similarity slider, to define the number of HPO terms that should match an ORDO term as well as the ORDO match scale, defining the specificity of the HPO term(s) to the selected ORDO term (based on a pre-computed matrix of their occurrence across all ORDO terms). Hence, when mapping ORDO to HPO terms, exact matching will traverse the mapping of these two term sets to find fewer but more specific HPO terms, while minimum matching will include more HPO terms but these may match other ORDO terms as well. Variant data cannot be filtered at the specific base-change level (as this would raise privacy concerns), but is instead queryable by host gene, allele frequency and mutation type using the Variant Query Builder. It is also possible to filter for variants based on affected biochemical pathways, given known relationships between genes and pathways (using the Reactome Knowledge base [58]*). Finally, the ERN dataset to be queried must be explicitly stated and requires that the user has permission to query the specified ERNs.*

5B: Discovery Nexus Query Results.

After submitting the query using the "Build query button" the system will return a count for matching results in the resources selected. Clicking on the number in the blue box will bring up the summary pop-up window as shown above, giving basic details of the matches (again subject to the user having been assigned permissions). The blue "Get Full Data for Selected Subjects" will open a link to request access from the resources holding the required data (where this is available). Alternatively, clicking the green button in the source details, will open a summary page with contact details for the resource, where a direct link to request the data is not available.

Table 1: Components Solve-RD project infrastructure

component	version	repository	licence	Documentation	Registration
RedIris Aspera		https://github.com/IBM/aspera-cli	BSD-3-Clause	https://www.rediris.es/rediris/	
RD-Connect GPAP and PhenoStore	2.28.0	https://platform.rd-connect.eu	NA	https://platform.rd-connect.eu/gpap_doc/	https://bio.tools/rd-connect_platform
Standardized Analysis Pipeline	20210521	https://github.com/inab/Wetlab2Variations/	Apache-2.0	https://pubmed.ncbi.nlm.nih.gov/27604516/	https://workflowhub.eu/workflows/107
MOLGENIS	10.1.0	https://github.com/molgenis/molgenis	GNU LGPL-3.0	https://github.com/molgenis	https://bio.tools/molgenis
Sandbox (deployment)	23.04.1	https://github.com/rug-cit-hpc/league-of-robots	GNU GPL v3.0	https://docs.gcc.rug.nl/fender/	
Sandbox (Ansible pipelines)	1.1.0	https://github.com/molgenis/ansible-pipelines	GNU GPL v3.0		
RD3 database	v1.0	https://github.com/molgenis/RD3_database	GNU LGPL-3.0		
RD3 solve-rd	v1.0	https://github.com/molgenis/projects-solve-rd	GNU LGPL-3.0	https://solve-rd.gcc.rug.nl/	
Cafe Variome	2.3.2	https://github.com/Cafe-Variome/CafeVariome/	MIT	https://cafe-variome.gitbook.io/	
Discovery Nexus	v2.0.0-alpha	https://github.com/Cafe-Variome/RDNexus	MIT	https://cafe-variome.gitbook.io/	
Beacon-2	cineca.2021.03	https://github.com/Cafe-Variome/beacon-2.x	Apache-2.0	https://docs.genomebeacons.org/	
MatchMaker Exchange	V1.1.1	https://github.com/ga4gh/mme-apis	NA	https://www.matchmakerexchange.org/	https://bio.tools/matchmaker_exchange
downloadclient (pyEGA3)	5.1.0	https://github.com/EGA-archive/ega-download-client	Apache-2.0	https://ega-archive.org/access/download/files/pyega3/	
ega-fuse-client	3.0.0	https://github.com/EGA-archive/ega-fuse-client	Apache-2.0	https://ega-archive.org/access/download/visualisation/fuse-client/	

Box 1: FAIR components of the Solve-RD infrastructure

Findability

- Infrastructure components are findable through bio.tools (GPAP), github (RD3, Discovery Nexus, sandbox).
- Raw data have globally Unique Identifiers (EGAD and EGAF).
- Samples of interest are findable through RD3/Discovery Nexus.
- Structuring results by DATF and DITF allows data to be findable based on both technique and disease.

Accessibility

- Archival of files in the EGA ensures long term accessibility of raw data.
- Phenotypic and variant data is accessible to registered users via GPAP.
- Metadata is stored separately from data through manifest file.
- Aspera servers, ega-fuse-client and download client can be used to transfer data to and from the EGA.
- Having multiple clusters accessible by all project members ensures accessibility of a data analysis infrastructure in case of maintenance.

Interoperability

- New HPC clusters can be deployed using Ansibl playbooks.
- Processing data starting from raw data using a standardized pipeline maximizes uniform output data.
- Output and export files follow file and ontology standards where possible.
- Seamless integration of RD3 and Discovery Nexus.
- GPAP provides interoperability via multiple APIs including Beacon-2 MatchMakerExchange, Ensembl, OMIM, Orphanet.

Reusability

- The creation of file patches allows for older versions of files to remain usable for reanalysis.
- Informed consent allows for data-analysis after DAC approval to data stored at EGA.

Data availability

Data is deposited at EGA. All raw and processed data files will be made available at the EGA (Solve-RD study EGAS00001003851) upon approval by data access committee. Access can be requested via the document in Supplementary information S2: Data Access Agreement. Accession numbers available datasets [12]. Current datasets are listed in Supplementary information S3: dataset specific conditions Pseudonymised phenotypic information for all individuals and their genetic variants are accessible through the RD-Connect GPAP [59] upon validated registration. The Ethics committee of the Eberhard Karl University of Tubingen gave ethical approval for this work.

List of abbreviations

AAI Authentication and authorization infrastructure
API Application Programming Interface
B1MG Beyond One Million Genomes
BBMRI Biobanking and Biomolecular Resources Research Infrastructure
C20 Number of bases covered by at least 20 sequencing reads
DAA Data Access Agreement
DAC Data Access Committee
DATF Data Interpretation Task Forces
DITF Data Interpretation Task Force
EGA European Genome-phenome Archive
EGAD EGA dataset
EGAF EGA File identifiers
E-ID experiment ID
EJP-RD European Joint Programme on Rare Diseases
ELIXIR European Life sciences infrastrucure
ERN European Reference Network
ES Exome Sequencing
FAIR Findable, Accessible, Interoperable and Reusable
FUSE Filesystem in Userspace
GA4GH Global Allicance for Genomics and Health

GDI Genome Data Infrastructure project
GDPR General Data Protection Regulation
GPAP RD-Connect Genome-Phenome Analysis Platform
GS Genome Sequencing
GUI graphical user interface
HPC High Performance Compute
HPO Human Phenotype Ontology
LR Long-read
MME Matchmaker Exchange
MT Mitochondria
OIDC open ID connect
OMIM Online Mendelian Inheritance in Man
ORDO Orphanet Rare Disease Ontology
P-ID participant ID
prm permanent storage
RD Rare Disease
RD3 Rare Disease Data about Data
SR Short-read
tmp temporary storage
TRE Trusted Research Environment
UID Unique Identifier
VRE Virtual Research Environment
WG Working Group

Supplementary data

Supplementary table S1: EGA manifest file

Supplementary information S1: Solve-RD Code of Conduct.

Supplementary figure S1: common workflows

Supplementary information S2: Dataset specific conditions

Supplementary information S3: Data Access Agreement

References

[1] Zurek B, Ellwanger K, Vissers LELM, et al. Solve-RD: systematic pan-European data sharing and collaborative analysis to solve rare diseases. Eur J Hum Genet 2021;29: 1325-31. https://doi.org/10.1038/s41431-021-00859-0.

[2] Laurie S, Piscia D, Matalong L, et al. The RD-Connect Genome-Phenome Analysis Platform: Accelerating diagnosis, research, and gene discovery for rare diseases. Hum Mutat 2022;43(6):717–33. https://doi.org/10.1002/humu.24353.

[3] Swertz MA, Dijkstra Mm, Adamusiak T, et al. The MOLGENIS toolkit: rapid prototyping of biosoftware at the push of a button. BMC Bioinformatics 2010;11(Supp12). https://doi.org/10.1186/1471-2105-11-S12-S12.

[4] van der Velde KJ, Imhann F, Charbon B, et al. MOLGENIS research: advanced bioinformatics data software for non-bioinformaticians. Bioinformatics 2019;35(6):1076-78. https://doi.org/10.1093/bioinformatics/bty742.

[5] Lancaster O, Beck T, Atlan D, et al. Cafe Variome: General-Purpose Software for Making Genotype–Phenotype Data Discoverable in Restricted or Open Access Contexts. Hum Mutation 2015;36(10):957-64. https://doi.org/10.1002/humu.22841.

[6] Rambla J, Baudis M, Ariosa R, et al. Beacon v2 and Beacon networks: A “lingua franca” for federated data discovery in biomedical genomics, and beyond. Hum Mutat 2022;**43**(6):791–99. https://doi.org/10.1002/humu.24369.

[7] Wilkinson M, Dumontier M, Aalbersberg IJ, et al. The FAIR Guiding Principles for scientific data management and stewardship. Sci Data 2016:3;160018. https://doi.org/10.1038/sdata.2016.18.

[8] Laurie S, Fernandez-Callejo F, Marco-Sola S, et al. From wet-lab to variations: concordance and speed of bioinformatics pipelines for whole genome and whole exome sequencing. Hum Mutat 2016:37(12): 1263-71. https://doi.org10.1002/humu.23114

[9] Kavianpour S, Sutherland J, Mansouri-Benssassi, et al. Next-Generation Capabilities in Trusted Research Environments: Interview Study. J Med Internet Res 2022;24(9):e33720. https://doi.org/10.2196/33720

[10] Boycott KM, Azzariti DR, Hamosh A, Rehm HL. Seven years since the launch of the Matchmaker Exchange: The evolution of genomic matchmaking. Hum Mutat *2022;*43(6):659–67. https://doi.org/10.1002/humu.24373.

[11] Fiume M, Cupak M, Keenan S, et al. Federated discovery and sharing of genomic data using Beacons. Nat Biotechnol;37(3):220–4. (2019). https://doi.org/10.1038/s41587-019-0046-x

[12] Solve-RD datasets. https://solve-rd.eu/results/solve-rd-data/. Accessed 15 Jul 2024.

[13] van der Velde KJ, Singh G, Kaliyaperumal R, et al. FAIR Genomes metadata schema promoting Next Generation Sequencing data reuse in Dutch healthcare and research. Sci Data 2022: 9(1);1–13. https://doi.org/10.1038/s41597-022-01265-x.

[14] Health-RI website. https://www.health-ri.nl/. Accessed 15 Jul 2024.

[15] ERDERA project website. https://www.ejprarediseases.org/erdera. Accessed 15 Jul 2024.

[16] Cock PJ, Fields CJ, Goto N, et al. The Sanger FASTQ file format for sequences with quality scores, and the Solexa/Illumina FASTQ variants. *Nucleic Acids Res 2010;*38(6):1767–71. https://doi.org/10.1093/nar/gkp1137

[17] Danecek P, Bonfield JK, Liddle J, et al. Twelve years of SAMtools and BCFtools. Gigascience. 2021 Feb16;10(2):giab008. doi: 10.1093/gigascience/giab008.

[18] Fritz MH-Y, Leinonen R, Cochrane G, Birney E. Efficient storage of high throughput DNA sequencing data using reference-based compression. Genome Res 2011;21(5):734-40. https://doi.org/10.1101/gr.114819.110

[19] Spanish academic and research network: RedIris. https://www.rediris.es/. Accessed 15-7-2024.

[20] Köhler S, Gargano M, Matentzoglu, et al. The Human Phenotype ontology in 2021. Nucleic Acids Res 2021;49(D1):D1207-D1217. https://doi.org10.1093/nar/gkaa1043.

[21] Vasant D, Chanas L, Malone J, et al. Ordo: an ontology connecting rare disease, epidemiology and genetic data. 2014, July. In *Proceedings of ISMB* (Vol. 30). researchgate.net. Ontologies can be obtained from: https://www.orphadata.com/ontologies/. Accessed 15 May 2024.

[22] Amberger, J.S., Bocchini, C.A., Schiettecatte, F., Scott, A.F. & Hamosh, A. OMIM.org: Online Mendelian Inheritance in Man (OMIM®), an online catalog of human genes and genetic disorders. *Nucleic Acids Research* **43**,(D1), D789–D798, (2015)

[23] Jacobsen JOB, Baudis M. Baynam GS, et al. The GA4GH Phenopacket schema defines a computable representation of clinical data. Nature Biotechnol 2022;40:817–20. https://doi.org/10.1038/s41587-022-01357-4.

[24] Chang CC, Chow CC, Tellier LC, Vattikuti S, Purcell SM, Lee JJ. Second-generation PLINK: ising to the challenge of larger and richer datasets. Gigascience. 2015 Feb 25;4:7. doi: 10.1186/s13742-015-0047-8.

[25] Caetano-Anolles D. PED – Pedigree format (Version September 30). https://gatk.broadinstitute.org/hc/en-us/articles/360035531972-PED-Pedigree-format. [Accessed 15 Jul 2024].

[26] GA4GH website. https://www.ga4gh.org/genomic-data-toolkit/. Accessed 15 Jul 2024.

[27] Caetano-Anolles, D. *GVCF - Genomic Variant Call Format*. (Version March 09, 2023). https://gatk.broadinstitute.org/hc/en-us/articles/360035531812-GVCF-Genomic-Variant-Call-Format. Accessed 15 Jul 2024.

[28] McLaren W, Gil L, Hunt SE, et al*.* The Ensembl Variant Effect Predictor. Genome Biol 2016;17:122. https://doi.org/10.1186/s13059-016-0974-4.

[29] Landrum MJ, Lee Jm, Benson M, et al. ClinVar: improving access to variant interpretations and supporting evidence. Nucleic Acids Res 2018:46(D1);D1062-D1067. https://doi.org/10.1093/nar/gkx1153

[30] Karczewski KJ, Francioli LC, Tlao G, et al. The mutational constraint spectrum quantified from variation in 141,456 humans. Nature 2020:581;434–43. https://doi.org/10.1038/s41586-020-2308-7.

[31] Martin AR, Williams E, Foulger RE, et al*.* PanelApp crowdsources expert knowledge to establish consensus diagnostic gene panels. Nat Genet 2019;51:1560–65. https://doi.org/10.1038/s41588-019-0528-2

[32] European Genome-Phenome Archive. https://ega-archive.org/. Accessed 15-7-2024

[33] Lappalainen I, Almeida-King J, Kumanduri V, et al*.* The European Genome-phenome Archive of human data consented for biomedical research. Nat Genet 2015;47, 692–95. https://doi.org/10.1038/ng.3312.

[34] Freeberg MA, Fromont LA, D'Altri T, et al. The European Genome-phenome Archive in 2021. Nucleic Acids Res 2022;50(D1):D980–D987. https://doi.org/10.1093/nar/gkab1059

[35] pyEGA3 github repository. https://github.com/EGA-archive/ega-download-client. Accessed 15-7-2024

[36] EGA fuse client github repository. https://github.com/EGA-archive/ega-fuse-client. Accessed 15-7-2024

[37] Corvò A, Matalonga L, Spalding D, et al. (2023). Remote visualization of large-scale genomic alignments for collaborative clinical research and diagnosis of rare diseases. *Cell Genom 2023;* 3(2):100246. https://doi.org/10.1016/j.xgen.2022.100246.

[38] Matalonga L, Hernández-Ferrer C, Piscia D, et al. Solving patients with rare diseases through programmatic reanalysis of genome-phenome data. Eur J Hum Genet 2021;29(9):1337–47. https://doi.org/10.1038/s41431-021-00852-7.

[39] Matchmaker Exchange API github repository. https://github.com/ga4gh/mme-apis. Accessed 15 Jul 2024.

[40] HPC cluster playbook github repository. https://github.com/rug-cit-hpc/league-of-robots. Accessed 15-7-2024.

[41] The CentOS Project. https://www.centos.org. Accessed 15 Jul 2024

[42] Spacewalk project. https://spacewalkproject.github.io. Accessed 15 Jul 2024.

[43] Lmod github repository. https://github.com/TACC/Lmod. Accessed 15 Jul 2024

[44] Easybuild github repository. https://github.com/easybuilders/easybuild. Accessed 15 Jul 2024.

[45] EMBL-EBI Embassy cloud. http://www.embassycloud.org/. Accessed 15 Jul 2024.

[46] Cook CE, Bergman MT, Finn RD, et al. The European Bioinformatics Institute in 2016: Data growth and integration. Nucleic Acids Res 2016;44(D1):D20–D26. https://doi.org/10.1093/nar/gkv1352.

[47] University Medical Center Groningen. Center for Information Technology. https://www.rug.nl/society-business/centre-for-information-technology/. Accessed 15 Jul 2024.

[48] Degen W, Scholtens S, Research Support in Nederland. De stand van zaken bij RUG en UMCG. *SURF*. 2019. https://www.surf.nl/files/2019-03/2018_rapport_research-support-in-nl_rug-umcg.pdf. Accessed 22 Mar 2023.

[49] The Gearshift High Performance Compute Cluster. http://docs.gcc.rug.nl/gearshift/. Accessed 15 Jul 2024.

[50] Salomaa, A. *Public-Key Cryptography*. Second edition. Springer-Verlag Berlin Heidelberg 1996. ISBN 978-3-662-03269-5 (eBook).

[51] WinSCP SFTP and FTP client. https://winscp.net. Accessed 15 Jul 2024.

[52] MobaXterm terminal for Windows. https://mobaxterm.mobatek.net. Accessed 15 Jul 2024.

[53] Cyberduck libre server and cloud storage browser. https://cyberduck.io. Accessed 15 Jul 2024.

[54] Rare Disease Data about Data github repository. https://github.com/molgenis/RD3_database. Accessed 15 Jul 2024.

[55] Solve-RD Rare Disease Data about Data database. https://solve-rd.gcc.rug.nl/. Accessed 15 Jul 2024.

[56] FusionAuth customer authentication platform. https://fusionauth.io/. Accessed 15 Jul 2024.

[57] Linden M, Prochazka M, Lappalainen I, et al. Common ELIXIR Service for Researcher Authentication and Authorisation. F1000Res 2018;7:ELIXIR-1199. https://doi.org/10.12688/f1000research.15161.1.

[58] Fabregate A, Jupe S, Matthews L, et al. The Reactome Pathway Knowledgebase. Nucleic Acids Res 2018;4;46(D1):D649-D655. https://doi.org/10.1093/nar/gkx1132.

[59] The Genome Phenome Analysis Platform. https://platform.rd-connect.eu/. Accessed 15 Jul 2024.

Funding

The Solve-RD project has received funding from the European Union's Horizon 2020 research and innovation programme under grant agreement No 779257. The RD-Connect Genome- Phenome Analysis Platform, received funding from EU projects RD-Connect, Solve-RD and EJP-RD (Grant Numbers FP7 305444, H2020 779257, H2020 825575), Instituto de Salud Carlos III (Grant Numbers PT13/0001/0044, PT17/0009/0019; Instituto Nacional de Bioinformática, INB) and ELIXIR Implementation Studies. The UMCG VRE and RD3 received funding from the EU projects Solve-RD, EJP-RD and CINECA Project (H2020 779257, H2020 825575, H2020 825775, respectively) and NWO VIDI grant number 917.164.455.

Acknowledgements

We acknowledge all Solve-RD partners (see Solve-RD consortium) and all hospitals and patients that shared data. We acknowledge RedIris (https://www.rediris.es/rediris/) for enabling data transfer from the data providers to GPAP.

Competing interests

The authors declare that they have no competing interests.

Corporate author lists

Solve-RD consortium

EKUT: Olaf Riess[1, 2], Tobias B. Haack[1], Holm Graessner[1, 2], Birte Zurek[1, 2], Kornelia Ellwanger[1, 2], Stephan Ossowski[1, 3], German Demidov[1], Marc Sturm[1], Julia M. Schulze-Hentrich[1], Rebecca Schüle[1, 2], Jishu Xu[4, 5], Christoph Kessler[4, 5], Melanie Kellner[4, 5], Matthis Synofzik[4, 5], Carlo Wilke[4, 5], Andreas Traschütz[4, 5], Ludger Schöls[4, 5], Holger Hengel[4, 5], Holger Lerche[1], Josua Kegele[6], Peter Heutink[4, 5]

RUMC: Han Brunner[7-9], Hans Scheffer[7, 8], Nicoline Hoogerbrugge[7, 10], Alexander Hoischen[7, 10, 11], Peter A.C. 't Hoen [10, 12], Lisenka E.L.M. Vissers[7, 8], Christian Gilissen[7, 10], Wouter Steyaert[7, 10], Karolis Sablauskas[7], Richarda M. de Voer[7, 10], Erik-Jan Kamsteeg[7], Bart van de Warrenburg[8, 13], Nienke van Os[8, 13], Iris te Paske[7, 10], Erik Janssen[7, 10], Elke de Boer[7, 8],Marloes Steehouwer[7], Burcu Yaldiz[7], Tjitske Kleefstra[7, 8]

University of Leicester: Anthony J. Brookes[14], Colin Veal[14], Spencer Gibson[14], Vatsalya Maddi[14], Mehdi Mehtarizadeh[14], Umar Riaz[14], Greg Warren[14], Farid Yavari Dizjikan[14], Thomas Shorter[14]

UNEW: Ana Töpf[15], Volker Straub[15], Chiara Marini Bettolo[15], Jordi Diaz Manera[15], Sophie Hambleton[16], Karin Engelhardt[16]

MUH: Jill Clayton-Smith[17, 18], Siddharth Banka[17, 18], Elizabeth Alexander[18], Adam Jackson[17, 18]

DIJON: Laurence Faivre[19-23], Christel Thauvin[19-23], Antonio Vitobello[21], Anne-Sophie Denommé-Pichon[21], Yannis Duffourd[21, 22], Ange-Line Bruel[21], Christine Peyron[24, 25], Aurore Pélissier[24, 25]

CNAG-CRG: Sergi Beltran[26, 27], Ivo Glynne Gut[26, 27], Steven Laurie[26], Davide Piscia[26], Leslie Matalonga[26], Anastasios Papakonstantinou[26], Gemma Bullich[26], Alberto Corvo[26], Marcos Fernandez-Callejo[26], Carles Hernández[26], Daniel Picó[26], Ida Paramonov[26], Hanns Lochmüller[26]

EURORDIS: Gulcin Gumus[28], Virginie Bros-Facer[29]

INSERM-Orphanet: Ana Rath[30], Marc Hanauer[30], David Lagorce[30],Oscar Hongnat[30],Maroua Chahdil[30],Emeline Lebreton[30]

INSERM-ICM: Giovanni Stevanin[31-35], Alexandra Durr[31-34, 36], Claire-Sophie Davoine[31-35], Léna Guillot-Noel[31-35], Anna Heinzmann [31-34, 37], Giulia Coarelli[31-34, 37]

INSERM-CRM: Gisèle Bonne[38], Teresinha Evangelista[38], Valérie Allamand[38], Isabelle Nelson[38], Rabah Ben Yaou[38-40], Corinne Metay[38, 41], Bruno Eymard[38, 39], Enzo Cohen[38], Antonio Atalaia[38], Tanya Stojkovic[38, 39]

Univerzita Karlova: Milan Macek Jr.[42], Marek Turnovec[42], Dana Thomasová[42], Radka Pourová Kremliková[42], Vera Franková[42], Markéta Havlovicová[42], Petra Lišková[43, 44], Pavla Doležalová[45]

EMBL-EBI: Helen Parkinson[46], Thomas Keane[46], Mallory Freeberg[46], Coline Thomas[46], Dylan Spalding[46]

Jackson Laboratory: Peter Robinson[47], Daniel Danis[47]

KCL: Glenn Robert[48], Alessia Costa[49], Christine Patch[49, 50]

UCL-IoN: Mike Hanna[51], Henry Houlden[52], Mary Reilly[51], Jana Vandrovcova[52], Stephanie Efthymiou[52], Heba Morsy[52], Elisa Cali[52], Francesca Magrinelli[53], Sanjay M. Sisodiya[54], Jonathan Rohrer[55]

UCL-ICH, Francesco Muntoni[56, 57], Irina Zaharieva[56], Anna Sarkozy[56]

Universiteit Antwerpen: Vincent Timmerman[58, 59], Jonathan Baets[60, 61], Geert de Vries[59, 60], Jonathan De Winter[59-61], Danique Beijer[58-60], Peter de Jonghe[59, 61], Liedewei Van de Vondel[58-60], Willem De Ridder[59-61], Sarah Weckhuysen[60, 62]

Uni Naples/Telethon UDP: Vincenzo Nigro[63, 64], Margherita Mutarelli[64, 65], Manuela Morleo[64], Michele Pinelli[64], Alessandra Varavallo[64], Sandro Banfi[63, 64], Annalaura Torella[63], Francesco Musacchia[63, 64], Giulio Piluso[63]

UNIFE: Alessandra Ferlini[66], Rita Selvatici[66], Francesca Gualandi[66], Stefania Bigoni[66], Rachele Rossi[66], Marcella Neri[66]

UKB: Stefan Aretz[67, 68], Isabel Spier[67, 68], Anna Katharina Sommer[67], Sophia Peters[67]

IPATIMUP: Carla Oliveira[69-71], Jose Garcia-Pelaez[69, 70, 72], Rita Barbosa-Matos[69, 70, 73], Celina São José[69, 70, 72] , Marta Ferreira[69, 70, 74], Irene Gullo[69-71, 75], Susana Fernandes[76], Luzia Garrido[75], Pedro Ferreira[69, 70, 77], Fátima Carneiro[69-71, 75]

UMCG: Morris A Swertz[78], Lennart Johansson[78], Joeri K van der Velde[78], Gerben van der Vries[78], Pieter B Neerincx[78], David Ruvolo[78], Kristin M Abbott[79], Wilhemina S Kerstjens Frederikse[79, 80], Eveline Zonneveld-Huijssoon[79, 81], Dieuwke Roelofs-Prins[78], Marielle van Gijn[79, 81]

Charité: Sebastian Köhler[82]

SHU: Alison Metcalfe[48, 83]

APHP: Alain Verloes[84, 85], Séverine Drunat[84, 85], Delphine Heron[86, 87], Cyril Mignot[86, 88], Boris Keren[86], Jean-Madeleine de Sainte Agathe[86]

CHU Bordeaux: Caroline Rooryck[89], Didier Lacombe[89], Aurelien Trimouille[90]

Spain UDP: Manuel Posada De la Paz[91], Eva Bermejo Sánchez[91], Estrella López Martín[91], Beatriz Martínez Delgado[91], F. Javier Alonso García de la Rosa[91]

Ospedale Pediatrico Bambino Gesù, Rome: Andrea Ciolfi[92], Bruno Dallapiccola[92], Simone Pizzi[92], Francesca Clementina Radio[92], Marco Tartaglia[92]

University of Siena: Alessandra Renieri[93-95], Simone Furini[93, 94], Chiara Fallerini[93, 94], Elisa Benetti[93, 94]

Semmelweis University Budapest: Peter Balicza[96], Maria Judit Molnar[96]

University of Ljubljana, Ales Maver[97], Borut Peterlin[97]

University of Lübeck: Alexander Münchau[98], Katja Lohmann[99], Rebecca Herzog[98, 100], Martje Pauly[98, 99]

Val d'Hebron Barcelona: Alfons Macaya[101, 102], Ana Cazurro-Gutiérrez[101], Belén Pérez-Dueñas[101], Francina Munell[101], Clara Franco Jarava[103, 104], Laura Batlle Masó[105, 106], Anna Marcé-Grau[101], Roger Colobran[103, 104, 107]

Hospital Sant Joan de Déu Barcelona: Andrés Nascimento Osorio[108], Daniel Natera de Benito[108]

University of Freiburg: Hanns Lochmüller[109-111], Rachel Thompson[111], Kiran Polavarapu[111], Bodo Grimbacher[112-116]

University of Oxford: David Beeson[117], Judith Cossins[117]

Folkhälsan Research Centre: Peter Hackman[118], Mridul Johari[118], Marco Savarese[118], Bjarne Udd[118-120]

University of Cambridge: Rita Horvath[121], Patrick F. Chinnery[121, 122], Thiloka Ratnaike[123], Fei Gao[121], Katherine Schon[121, 124]

Catalan Institute of Oncology, Barcelona: Gabriel Capella[125], Laura Valle[125]

KU Munich: Elke Holinski-Feder[126], Andreas Laner[127], Verena Steinke-Lange[126]

TU Dresden: Evelin Schröck[128], Andreas Rump[128, 129]

Koç University: Ayşe Nazlı Başak[130]

Ghent University Hospital: Dimitri Hemelsoet[131, 132], Bart Dermaut[132-134], Nika Schuermans[132-134], Bruce Poppe[132-134], Hannah Verdin[133]

University Hospital Meyer, Florence: Davide Mei[135], Annalisa Vetro[135], Simona Balestrini[135, 136], Renzo Guerrini[135]

KU Leuven: Kristl Claeys[137, 138]

LUMC: Gijs W.E. Santen[139], Emilia K. Bijlsma[139], Mariette J.V. Hoffer[139], Claudia A.L. Ruivenkamp[139]

Ludwig Boltzmann Institute for Rare and Undiagnosed Diseases, Vienna: Kaan Boztug[140-144], Matthias Haimel[140-142]

Institute of Pathology and Genetics, Gosselies, Belgium: Isabelle Maystadt[145, 146]

Technical University Munich: Isabell Cordts[147], Marcus Deschauer[147]

Neurology/Neurogenetics Laboratory University of Crete, Heraklion, Crete, Greece: Ioannis Zaganas[148], Evgenia Kokosali[148], Mathioudakis Lambros[148], Athanasios Evangeliou[149], Martha Spilioti[150], Elisabeth Kapaki[151], Mara Bourbouli[151]

IRCCS G. Gaslini: Pasquale Striano[152, 153], Federico Zara[153, 154], Antonella Riva[153, 154], Michele Iacomino[154, 155], Paolo Uva[155], Marcello Scala[152, 153], Paolo Scudieri[153, 154]

Cliniques universitaires Saint-Luc (CUSL): Maria-Roberta Cilio[156], Evelina Carpancea[156], Chantal Depondt[157], Damien Lederer[158], Yves Sznajer[159], Sarah Duerinckx[160], Sandrine Mary[158]

Institute of Human Genetics, University Hospital Essen: Christel Depienne[161, 162], Andreas Roos[111, 163, 164]
University of Luxembourg: Patrick May[165]

Affiliations

1. Institute of Medical Genetics and Applied Genomics, University of Tübingen, Tübingen, Germany.
2. Centre for Rare Diseases, University of Tübingen, Tübingen, Germany.
3. NGS Competence Center Tübingen (NCCT), University of Tübingen, Tübingen, Germany.
4. Department of Neurodegeneration, Hertie Institute for Clinical Brain Research (HIH), University of Tübingen, Tübingen, Germany.
5. German Center for Neurodegenerative Diseases (DZNE), Tübingen, Germany.
6. Department of Neurology and Epileptology, Hertie Institute for Clinical Brain Research (HIH), University of Tübingen, Tübingen, Germany.
7. Department of Human Genetics, Radboud University Medical Center, Nijmegen, The Netherlands.
8. Donders Institute for Brain, Cognition and Behaviour, Radboud University Medical Center, Nijmegen, The Netherlands.
9. Department of Clinical Genetics, Maastricht University Medical Centre, Maastricht, the Netherlands.
10. Radboud Institute for Molecular Life Sciences, Nijmegen, The Netherlands.
11. Department of Internal Medicine and Radboud Center for Infectious Diseases (RCI), Radboud University Medical Center, Nijmegen, the Netherlands.

12. Center for Molecular and Biomolecular Informatics, Radboud University Medical Center, Nijmegen, the Netherlands.
13. Department of Neurology, Radboud University Medical Center, Nijmegen, The Netherlands.
14. Department of Genetics and Genome Biology, University of Leicester, Leicester, UK.
15. John Walton Muscular Dystrophy Research Centre, Translational and Clinical Research Institute, Newcastle University and Newcastle Hospitals NHS Foundation Trust, Newcastle upon Tyne, UK.
16. Primary Immunodeficiency Group, Translational and Clinical Research Institute, Newcastle University and Newcastle upon Tyne Hospitals NHS Foundation Trust, Newcastle upon Tyne, UK.
17. Division of Evolution, Infection and Genomics, School of Biological Sciences, Faculty of Biology, Medicine and Health, University of Manchester, Manchester M13 9WL, UK.
18. Manchester Centre for Genomic Medicine, St Mary's Hospital, Manchester University Hospitals NHS Foundation Trust, Health Innovation Manchester, Manchester M13 9WL, UK.
19. Dijon University Hospital, Genetics Department, Dijon, France.
20. Dijon University Hospital, Centre of Reference for Rare Diseases: Development disorders and malformation syndromes, Dijon, France.
21. Inserm - University of Burgundy-Franche Comté, UMR1231 GAD, Dijon, France.
22. Dijon University Hospital, FHU-TRANSLAD, Dijon, France.
23. Dijon University Hospital, GIMI institute, Dijon, France.
24. University of Burgundy-Franche Comté, Dijon Economics Laboratory, Dijon, France.
25. University of Burgundy-Franche Comté, FHU-TRANSLAD, Dijon, France.
26. CNAG-CRG, Centre for Genomic Regulation (CRG), The Barcelona Institute of Science and Technology, Baldiri Reixac 4, Barcelona 08028, Spain.
27. Universitat Pompeu Fabra (UPF), Barcelona, Spain.
28. EURORDIS-Rare Diseases Europe, Sant Antoni Maria Claret 167 - 08025 Barcelona, Spain.
29. EURORDIS-Rare Diseases Europe, Plateforme Maladies Rares, 75014 Paris, France.
30. INSERM, US14 - Orphanet, Plateforme Maladies Rares, 75014 Paris, France.
31. Institut National de la Santé et de la Recherche Medicale (INSERM) U1127, Paris, France.

32. Centre National de la Recherche Scientifique, Unité Mixte de Recherche (UMR) 7225, Paris, France.
33. Unité Mixte de Recherche en Santé 1127, Université Pierre et Marie Curie (Paris 06), Sorbonne Universités, Paris, France.
34. Institut du Cerveau - ICM, Paris, France.
35. Ecole Pratique des Hautes Etudes, Paris Sciences et Lettres Research University, Paris, France.
36. Centre de Référence de Neurogénétique, Hôpital de la Pitié-Salpêtrière, Assistance Publique-Hôpitaux de Paris (AP-HP), Paris, France.
37. Hôpital de la Pitié-Salpêtrière, Assistance Publique-Hôpitaux de Paris (AP-HP), Paris, France.
38. Sorbonne Université, Inserm, Institut de Myologie, Centre de Recherche en Myologie, F-75013 Paris, France.
39. AP-HP, Centre de Référence de Pathologie Neuromusculaire Nord, Est, Ile-de-France, Institut de Myologie, G.H. Pitié-Salpêtrière, F-75013 Paris, France.
40. Institut de Myologie, Equipe Bases de données, G.H. Pitié-Salpêtrière, F-75013 Paris, France.
41. AP-HP, Unité Fonctionnelle de Cardiogénétique et Myogénétique Moléculaire et Cellulaire, G.H. Pitié-Salpêtrière, F-75013 Paris, France.
42. Department of Biology and Medical Genetics, Charles University Prague-2nd Faculty of Medicine and University Hospital Motol, Prague, Czech Republic.
43. Department of Paediatrics and Inherited Metabolic Disorders, First Faculty of Medicine, Charles University and General University Hospital in Prague, Prague, Czech Republic.
44. Department of Ophthalmology, First Faculty of Medicine, Charles University and General University Hospital in Prague, Prague, Czech Republic.
45. Centre for Paediatric Rheumatology and Autoinflammatory Diseases, Department of Paediatrics and Inherited Metabolic Disorders, 1st Faculty of Medicine, Charles University and General University Hospital in Prague, Czech Republic.
46. European Bioinformatics Institute, European Molecular Biology Laboratory, Wellcome Genome Campus, Hinxton, Cambridge, United Kingdom.
47. Jackson Laboratory for Genomic Medicine, Farmington, CT 06032, USA.
48. Florence Nightingale Faculty of Nursing, Midwifery & Palliative Care, King's College, London, UK.

49. Society and Ethics Research, Connecting Science, Wellcome Genome Campus,
Hinxton, UK.
50. Genomics England, Queen Mary University of London, Dawson Hall, EC1M 6BQ, London, UK.
51. MRC Centre for Neuromuscular Diseases and National Hospital for Neurology and Neurosurgery, UCL Queen Square Institute of Neurology, London, UK.
52. Department of Neuromuscular Diseases, UCL Queen Square Institute of Neurology, London, UK.
53. Department of Clinical and Movement Neurosciences, UCL Queen Square Institute of Neurology, University College London, WC1N 3BG.
54. Department of Clinical and Experimental Epilepsy, UCL Queen Square Institute of Neurology, London, UK.
55. Dementia Research Centre, Department of Neurodegenerative Disease, UCL Queen Square Institute of Neurology, London, UK.
56. Dubowitz Neuromuscular Centre, UCL Great Ormond Street Hospital, London, UK.
57. NIHR Great Ormond Street Hospital Biomedical Research Centre, London, United Kingdom.
58. Peripheral Neuropathy Research Group, University of Antwerp, Antwerp, Belgium.
59. Laboratory of Neuromuscular Pathology, Institute Born-Bunge, University of Antwerp, Antwerpen, Belgium.
60. Translational Neurosciences, Faculty of Medicine and Health Sciences, University of Antwerp, Belgium.
61. Neuromuscular Reference Centre, Department of Neurology, Antwerp University Hospital, Antwerpen, Belgium.
62. VIB-CMN, Applied and Translational Neurogenomics Group.
63. Dipartimento di Medicina di Precisione, Università degli Studi della Campania "Luigi Vanvitelli", Napoli, Italy.
64. Telethon Institute of Genetics and Medicine, Pozzuoli, Italy.
65. Istituto di Scienze Applicate e Sistemi Intelligenti "E.Caianiello" - ISASI - CNR.
66. Unit of Medical Genetics, Department of Medical Sciences, University of Ferrara, Italy.
67. Institute of Human Genetics, Medical Faculty, University of Bonn, Bonn, Germany.
68. Center for Hereditary Tumor Syndromes, University Hospital Bonn, Bonn, Germany.

69. i3S - Instituto de Investigação e Inovação em Saúde, Universidade do Porto, Portugal.
70. IPATIMUP - Institute of Molecular Pathology and Immunology of the University of Porto, Portugal.
71. Faculty of Medicine, University of Porto, Portugal.
72. Doctoral Programme in Biomedicine, Faculty of Medicine, University of Porto, Portugal.
73. Doctoral Programme in BiotechHealth, School of Medicine and Biomedical Sciences, University of Porto, Portugal.
74. Doctoral Programme in Computer Science, Faculty of Sciences, University of Porto, Portugal.
75. CHUSJ, Centro Hospitalar e Universitário de São João, Porto, Portugal.
76. Departament of Genetics, Faculty of Medicine, University of Porto, Portugal.
77. Faculty of Sciences, University of Porto, Portugal.
78. Department of Genetics, Genomics Coordination Center, University Medical Center Groningen, University of Groningen, Groningen, The Netherlands.
79. Department of Genetics, University Medical Center Groningen, University of Groningen, Groningen, The Netherlands.
80. ERN-GENTURIS.
81. ERN-RITA: European Reference Network for Immunodeficiency, Autoinflammatory, Autimmune and Paediatric Rheumatic diseases, Utrecht, Netherlands.
82. Ada Health GmbH, Karl-Liebknecht-Str. 1, 10178 Berlin, Germany.
83. College of Health, Well-being and Life-Sciences, Sheffield Hallam University, Sheffield, UK.
84. Dept of Genetics, Assistance Publique-Hôpitaux de Paris - Université de Paris, Robert DEBRE University Hospital, 48 bd SERURIER, Paris, France.
85. INSERM UMR 1141 "NeuroDiderot", Hôpital Robert DEBRE, Paris, France.
86. Department of Genetics, Assistance Publique-Hôpitaux de Paris - Sorbonne Université, Pitié-Salpêtrière University Hospital, 83 Boulevard de l'Hôpital, Paris, France.
87. Reference center of rare diseases "intellectuel disability of rare causes", Paris, France.
88. Institut du Cerveau (ICM), UMR S 1127, Inserm U1127, CNRS UMR 7225, Sorbonne Université, 75013, Paris, France.

89. Univ. Bordeaux, MRGM INSERM U1211, CHU de Bordeaux, Service de Génétique Médicale , F-33000 Bordeaux, France.
90. Laboratoire de Génétique Moléculaire, Service de Génétique Médicale, CHU Bordeaux – Hôpital Pellegrin, Place Amélie Raba Léon, 33076 Bordeaux Cedex, France.
91. Institute of Rare Diseases Research, Spanish Undiagnosed Rare Diseases Cases Program (SpainUDP) & Undiagnosed Diseases Network International (UDNI), Instituto de Salud Carlos III, Madrid, Spain.
92. Molecular Genetics and Functional Genomics, Ospedale Pediatrico Bambino Gesù, IRCCS, Rome, Italy.
93. Med Biotech Hub and Competence Center, Department of Medical Biotechnologies, University of Siena, Italy.
94. Medical Genetics, University of Siena, Italy.
95. Genetica Medica, Azienda Ospedaliero-Universitaria Senese, Italy.
96. Institute of Genomic Medicine and Rare Diseases, Semmelweis University, Budapest, Hungary.
97. Clinical Institute of Genomic Medicine, University Medical Centre Ljubljana, Slovenia.
98. Institute of Systems Motor Science, University of Lübeck, Ratzeburger Allee 160, 23562, Lübeck, Germany.
99. Institute of Neurogenetics, University of Lübeck, Ratzeburger Allee 160, 23562, Lübeck, Germany.
100. Department of Neurology, University Hospital Schleswig Holstein, Ratzeburger Allee 160, 23562, Lübeck, Germany.
101. Pediatric Neurology Research Group, Vall d’Hebron Research Institute, Universitat Autònoma de Barcelona, Barcelona, Spain.
102. Institute of Neuroscience, Universitat Autònoma de Barcelona, Barcelona, Spain.
103. Diagnostic Immunology Research Group, Vall d’Hebron Research Institute (VHIR), Barcelona, Spain.
104. Immunology Division, Genetics Department. Vall d'Hebron University Hospital (HUVH), Barcelona, Spain.
105. Infection in Immunocompromised Pediatric Patients Research Group, Vall d’Hebron Research Institute (VHIR), Barcelona, Spain.
106. Pediatric Infectious Diseases and Immunodeficiencies Unit, Vall d’Hebron University Hospital (HUVH),Barcelona, Spain.
107. Immunology Unit. Department of Cell Biology, Physiology and Immunology. Autonomous University of Barcelona (UAB), Bellaterra, Spain.

108. Neuromuscular Disorders Unit , Department of Pediatric Neurology. Hospital Sant Joan de Déu, Barcelona, Spain
109. Department of Neuropediatrics and Muscle Disorders, Medical Center, Faculty of Medicine, University of Freiburg, Freiburg, Germany.
110. Centro Nacional de Análisis Genómico (CNAG-CRG), Center for Genomic Regulation, Barcelona Institute of Science and Technology (BIST), Barcelona, Spain.
111. Children's Hospital of Eastern Ontario Research Institute, University of Ottawa, Ottawa, Canada.
112. Institute for Immunodeficiency, Center for Chronic Immunodeficiency (CCI), Medical Center, Faculty of Medicine, Albert-Ludwigs-University of Freiburg, Germany.
113. Clinic of Rheumatology and Clinical Immunology, Center for Chronic Immunodeficiency (CCI), Medical Center, Faculty of Medicine, Albert-Ludwigs-University of Freiburg, Germany.
114. DZIF – German Center for Infection Research, Satellite Center Freiburg, Germany.
115. CIBSS – Centre for Integrative Biological Signalling Studies, Albert-Ludwigs University, Freiburg, Germany.
116. RESIST – Cluster of Excellence 2155 to Hanover Medical School, Satellite Center Freiburg, Germany.
117. Nuffield Department of Clinical Neurosciences, University of Oxford, UK.
118. Folkhälsan Research Centre and Medicum, University of Helsinki, Helsinki, Finland.
119. Tampere Neuromuscular Center, Tampere, Finland.
120. Vasa Central Hospital, Vaasa, Finland.
121. Department of Clinical Neurosciences, University of Cambridge, Cambridge, UK.
122. Medical Research Council Mitochondrial Biology Unit, University of Cambridge, Cambridge, UK.
123. Department of Paediatrics, University of Cambridge, Cambridge, UK.
124. East Anglian Medical Genetics Service, Cambridge University Hospitals NHS Foundation Trust, Cambridge, UK.
125. Bellvitge Biomedical Research Institute (IDIBELL), Barcelona, Spain.
126. Medizinische Klinik und Poliklinik IV – Campus Innenstadt, Klinikum der Universität München, Munich, Germany.
127. MGZ - Medical Genetics Center, Munich, Germany.
128. Institute of Clinical Genetics, University Hospital Carl Gustav Carus, Technical University Dresden, Dresden, Germany.

129. Center for Personalized Oncology, University Hospital Carl Gustav Carus, Technical University Dresden, Dresden, Germany.
130. Koç Universıty,School of Medicine, Translational Medicine Research Center, KUTTAM-NDAL Istanbul Turkey.
131. Dpt. of Neurology, Ghent University Hospital, Ghent, Belgium.
132. Program for Undiagnosed Rare Diseases (UD-PrOZA), Ghent University Hospital, Ghent, Belgium.
133. Center for Medical Genetics, Ghent University Hospital, Ghent, Belgium.
134. Department of Biomolecular Medicine, Faculty of Medicine and Health Sciences, Ghent University, Ghent, Belgium.
135. Neuroscience Department, Children's Hospital A. Meyer-University of Florence, 50139, Florence, Italy.
136. Department of Clinical and Experimental Epilepsy, UCL Queen Square Institute of Neurology, and Chalfont Centre for Epilepsy, Gerrard Cross, UK.
137. Department of Neurology, University Hospitals Leuven, Leuven, Belgium.
138. Laboratory for Muscle Diseases and Neuropathies, Department of Neurosciences, and Leuven Brain Institute (LBI), KU Leuven - University of Leuven, Leuven, Belgium.
139. Department of Clinical Genetics, Leiden University Medical Center, Leiden, The Netherlands.
140. Ludwig Boltzmann Institute for Rare and Undiagnosed Diseases, Vienna, Austria.
141. St. Anna Children’s Cancer Research Institute (CCRI), Vienna, Austria.
142. CeMM Research Center for Molecular Medicine of the Austrian Academy of Sciences, Vienna, Austria.
143. Department of Pediatrics and Adolescent Medicine, Medical University of Vienna, Vienna, Austria.
144. St. Anna Children's Hospital, Department of Pediatrics and Adolescent Medicine, Medical University of Vienna, Vienna, Austria.
145. Centre de Génétique Humaine, Institut de Pathologie et de Génétique, Gosselies, Belgium.
146. Département de Médecine, Université de namur (Unamur), Namur, Belgique.
147. Department of Neurology, Klinikum rechts der Isar, Technical University Munich, Munich, Germany.
148. Neurology / Neurogenetics Laboratory University of Crete, Heraklion, Crete, Greece.
149. Aristotle University of Thessaloniki, Thessaloniki, Greece.

150. 1st Department of Neurology, Aristotle University of Thessaloniki, University General Hospital of Thessaloniki, AHEPA, Thessaloniki, Greece.
151. Neurochemistry and Biomarker Unit, 1st Department of Neurology, School of Medicine, National and Kapodistrian University of Athens, Eginition Hospital, Athens, Greece.
152. Pediatric Neurology and Muscular Disease Unit, IRCCS Istituto Giannina Gaslini, Genoa, Italy.
153. Department of Neurosciences, Rehabilitation, Ophthalmology, Genetics, Maternal and Child Health, University of Genoa, Genoa, Italy.
154. Unit of Medical Genetics, IRCCS Istituto Giannina Gaslini, Genoa, Italy.
155. Clinical Bioinformatics, IRCCS Istituto Giannina Gaslini, Genoa, Italy.
156. Pediatric Neurology Department, Saint-Luc University Hospital, Université Catholique de Louvain, Brussels, Belgium.
157. Neurology Department, Erasme Hospital, Université Libre de Bruxelles , Bruxelles, Belgium.
158. Institute of Pathology and Genetics, Charleroi, Belgium.
159. Human Genetics Department, Saint-Luc University Hospital, Université Catholique de Louvain, Brussels, Belgium.
160. Institute of Interdisciplinary Research in Human and Molecular Biology, Human Genetics, IRIBHM, Université Libre de Bruxelles, Brussels, Belgium.
161. Institute of Human Genetics, University Hospital Essen, University Duisburg-Essen, Essen, Germany.
162. Institut du Cerveau et de la Moelle épinière (ICM), Sorbonne Université, UMR S 1127, Inserm U1127, CNRS UMR 7225, F-75013 Paris, France.
163. Department of Pediatric Neurology, Developmental Neurology and Social Pediatrics, Children's Hospital University of Essen, Essen, Germany.
164. Department of Neurology, Heimer Institute for Muscle Research, University Hospital Bergmannsheil, Ruhr-University Bochum, 44789 Bochum, Germany.
165. Luxembourg Centre for Systems Biomedicine, University of Luxembourg, Esch-sur-Alzette, Luxembourg.

Figure 1

Click here to access/download;Figure;Figure1_v2.jpg

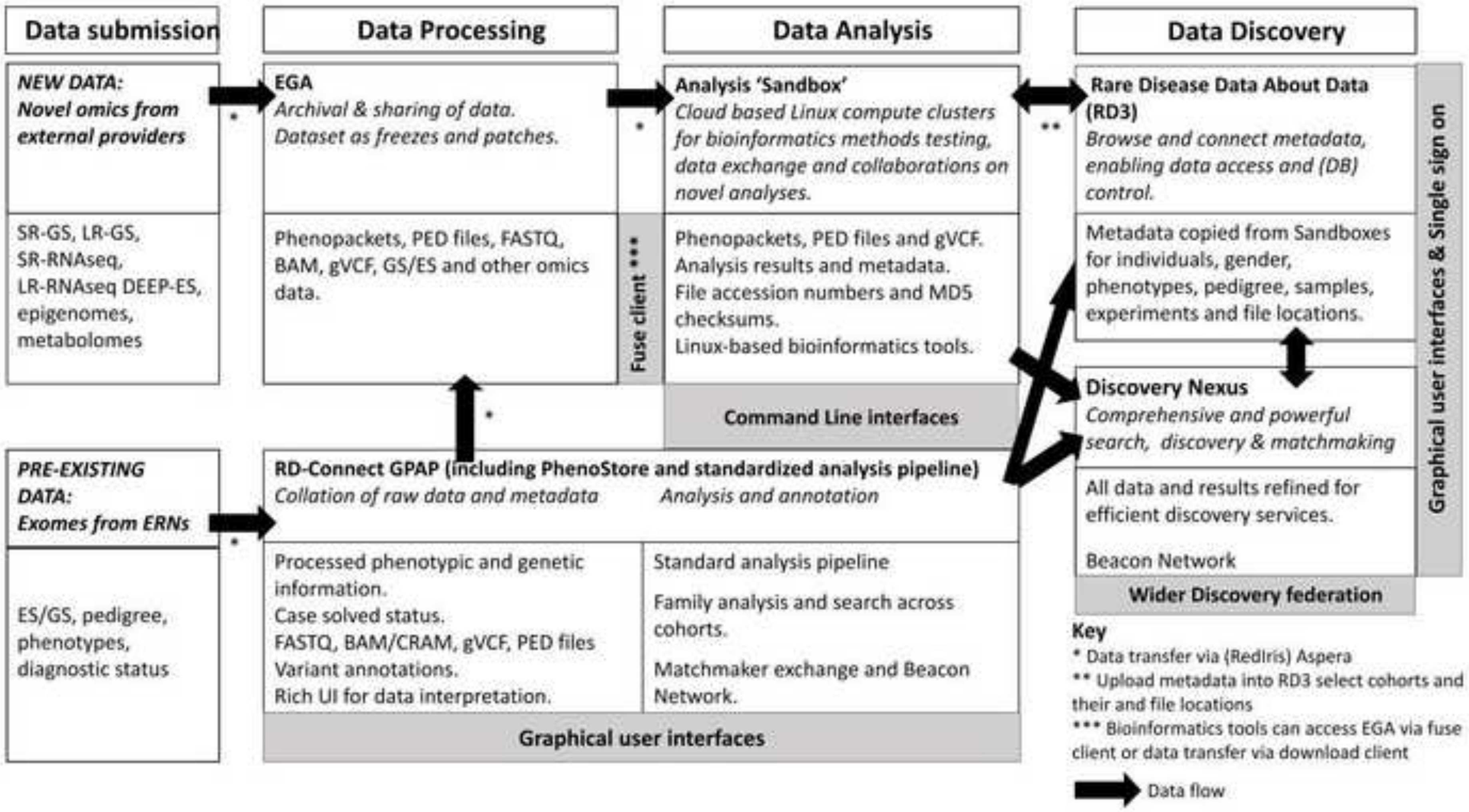

Figure 2

Click here to access/download;Figure;Figure2_scheme_folderstructure_sandbox.jpg

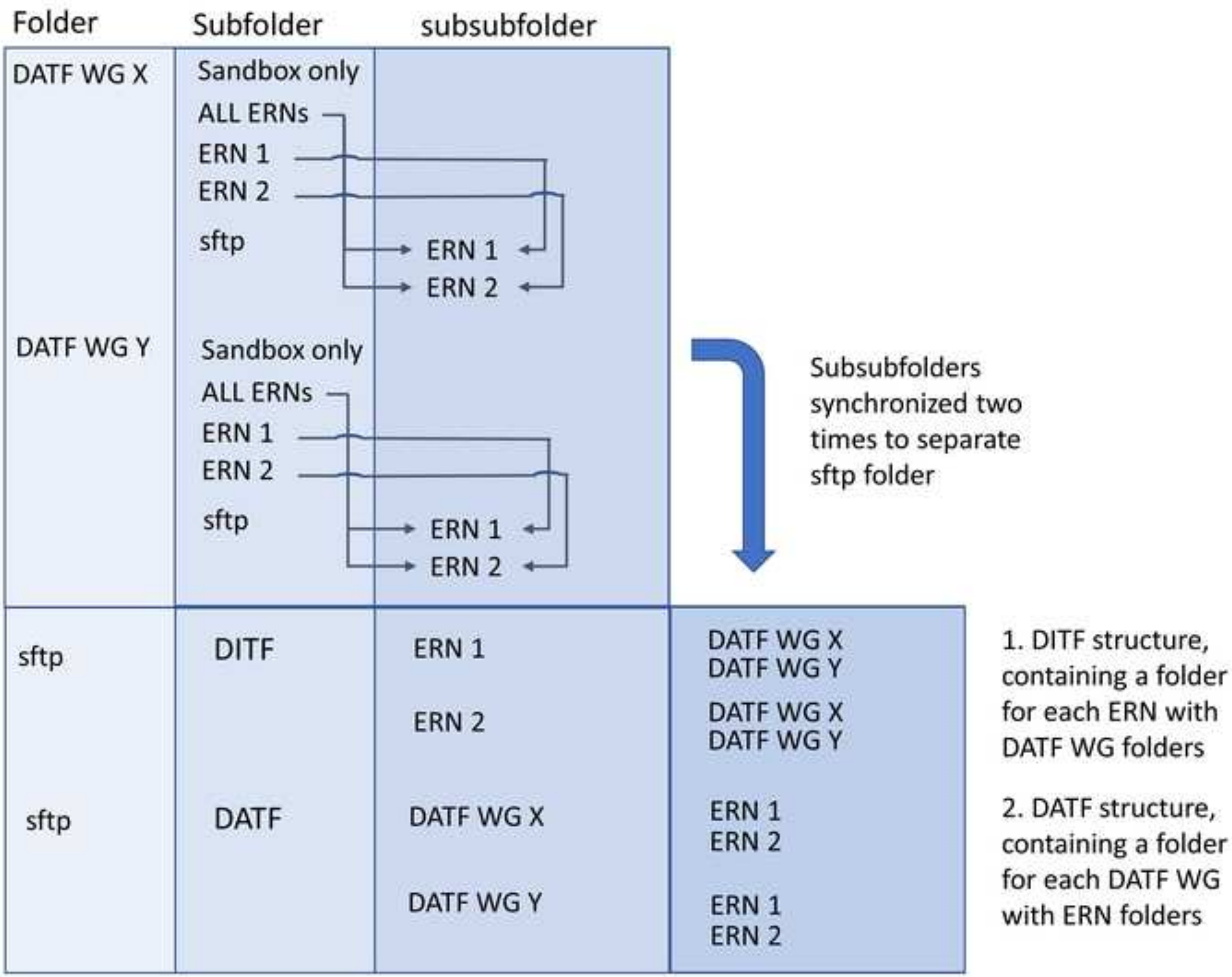

Figure 3

Click here to access/download;Figure;Figure3_300dpi.jpg

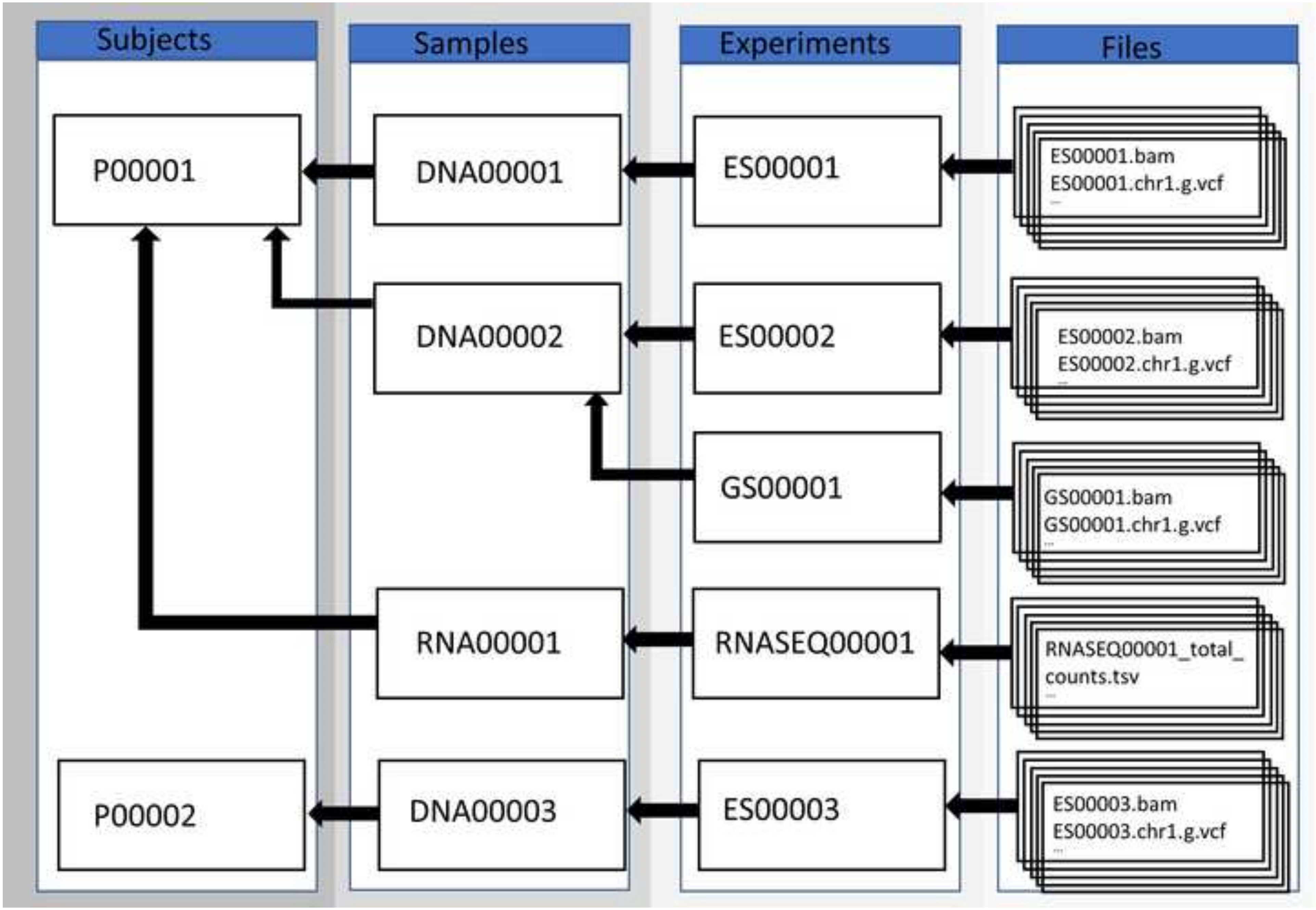

Figure 4

Click here to access/download;Figure;Figure4_300dpi.tif

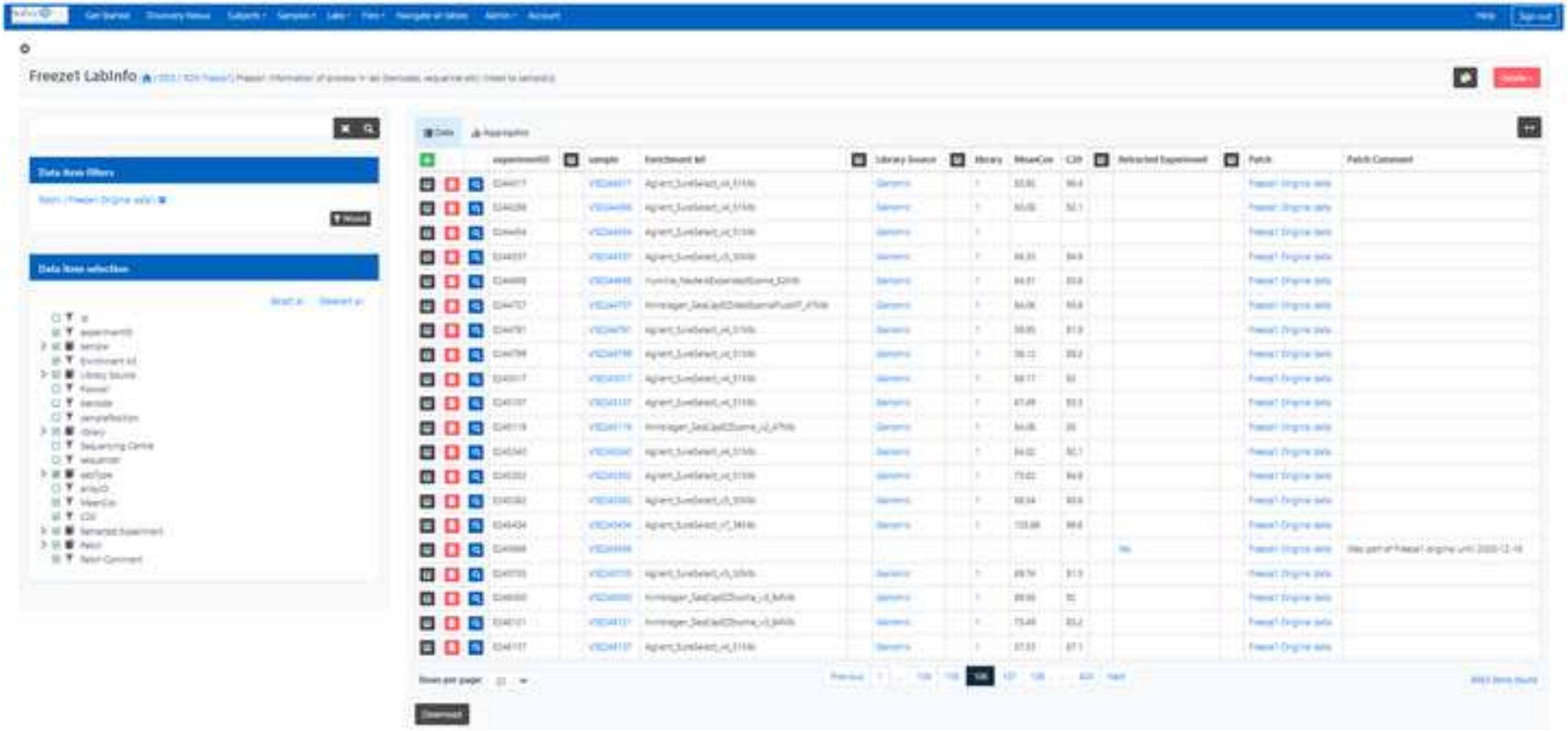

RD3_LabInfo_2.tif

Figure 5a

Click here to access/download;Figure;Figure5A_300dpi.jpg

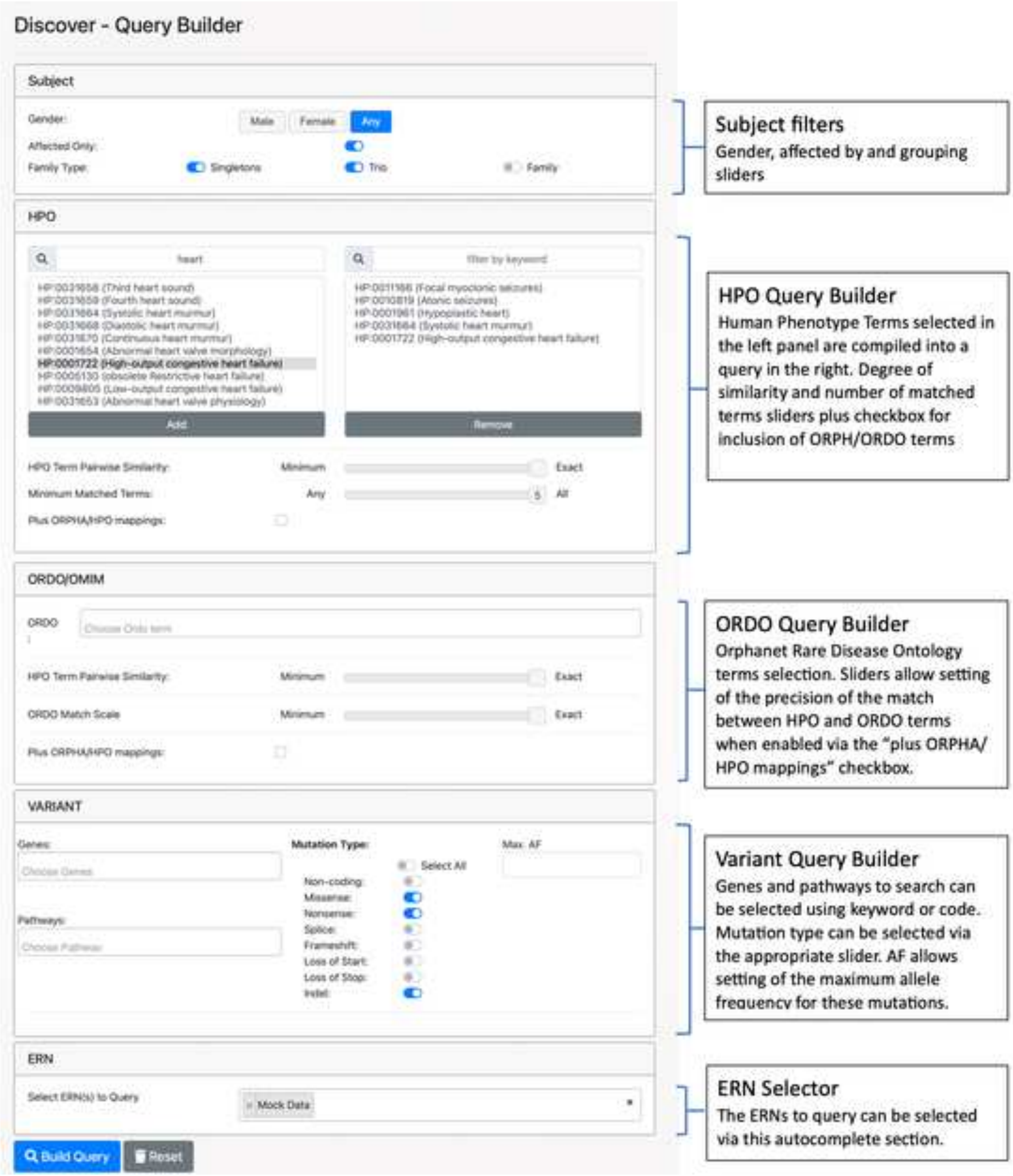

Figure 5b

Click here to access/download;Figure;Figure5B 300_dpi.jpg

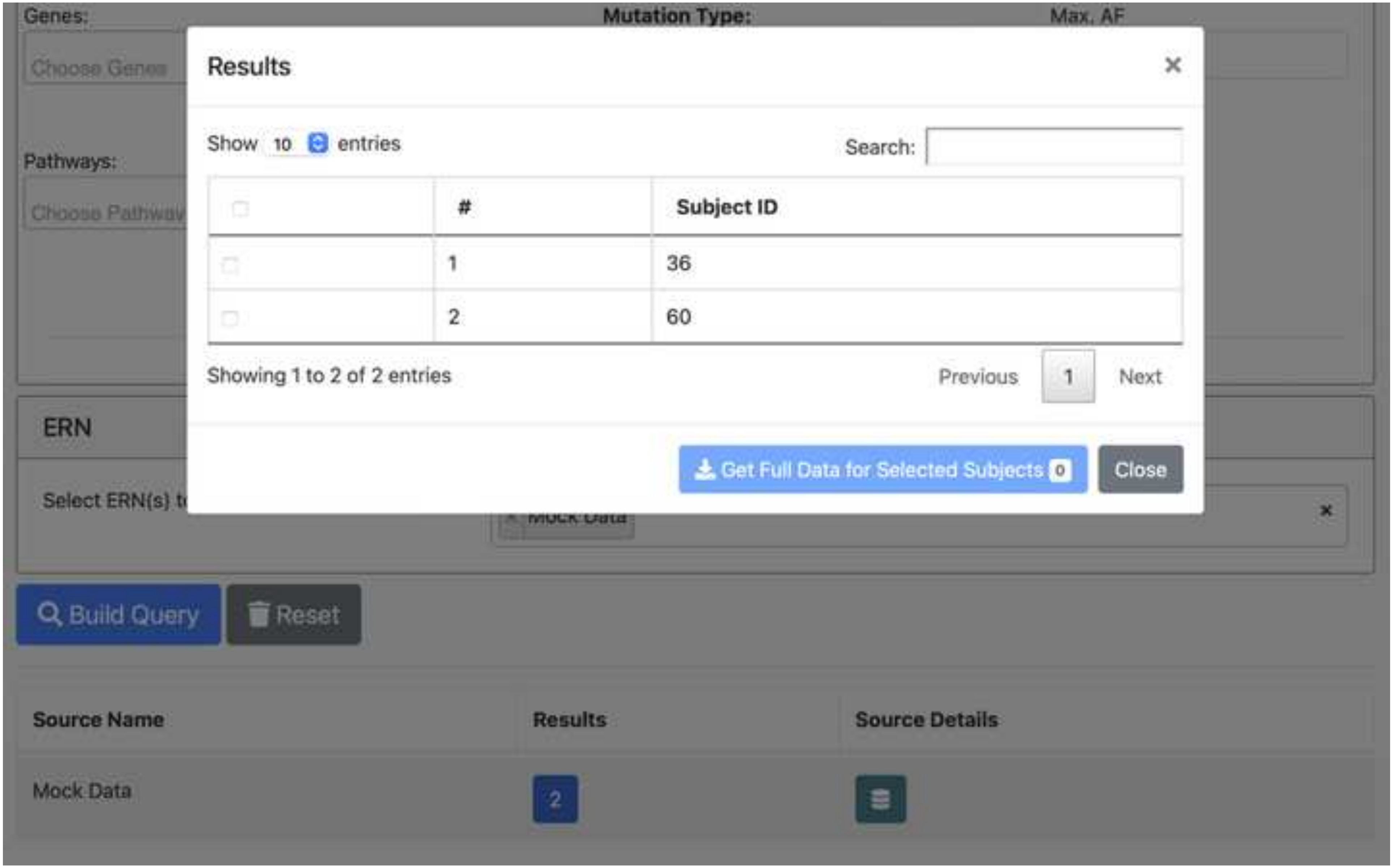

Supplementary Table S1

Click here to access/download
Supplementary Material
Table_S1_Supplementary Material.xlsx

Click here to access/download
Supplementary Material
Supplementary_information_1_Supplementary Material.pdf

Supplementary Figure S1

Click here to access/download
Supplementary Material
Figure_S1_Supplementary Material.png

Fig S2

Click here to access/download

Supplementary Material

Supplementary_information_S2_dataset_specific_conditions.docx

Fig S3

Click here to access/download
Supplementary Material
Supplementary_information_S3_Data_access_agreement.docx
