# Supplementary material for: An interconnected data infrastructure to support large-scale rare disease research
Source: Gigascience. 2024 Sep 20;13:giae058. doi: 10.1093/gigascience/giae058 (PMC11413801; doi:10.1093/gigascience/giae058)
Supplement: giae058_Supplemental_Files [file giae058_supplemental_files.zip › Supplementary_information_S2_dataset_specific_conditions.docx]

**Appendix A: Dataset Specific Conditions**

**Version 1 (10.10.2023)**

The patient samples for the Solve-RD Project were in some cases pre-existing prior to the start of the project but in most cases have been collected within the project. While they have all been approved for analysis and use in the Project, and for the resulting data to be used by others according to the Solve-RD Data Sharing Policy which this Data Access Agreement implements, in some cases the original consents restrict the uses to which the data can be put.

For the sake of clarity, we list here all datasets, and any restrictions on use that apply. We also list the correct way to reference the origin of each sample set, as required in acknowledgements.

Some of the Solve-RD disease (not cohort) studies have Research Ethical Committee approval to feedback to individual research participants genetic results that cause the clinical phenotype that is being studied. We encourage researchers who believe that they have identified a causal variant(s) for the disease under investigation by the Solve-RD project to contact the Solve-RD Project at [solve-RD@med.uni-tuebingen.de](mailto:solve-RD@med.uni-tuebingen.de) who will ensure that the information is passed on to the relevant sample custodian, for their consideration.

Please note that NONE of the Solve-RD Projects have Research Ethical Committee approval to feedback to individual research participants genetic results that do not pertain to the clinical phenotype under investigation (so-called 'Incidental Findings'), and so such results SHOULD NOT be returned to the Data Access Committee, or directly to members of the Solve-RD Project or sample custodians.

If the datasets that You are requesting access for are not listed below, then You must obtain a more recent version of this appendix from [www.solve-rd.eu](http://www.solve-rd.eu) and refer to that in Your access application.

| **Solve-RD_ITHACA_cohort-1_DF1+2_V1** | |
| --- | --- |
| EGA Study ID: EGAS00001003851 \| EGA dataset ID: EGAD00001009770 | |
| **Brief description:** | This dataset includes genomic (WES and WGS) and phenotypic data as well as pedigree information from unsolved rare disease patients with developmental diseases (and their relatives) which have been contributed by ERN-ITHACA partners and the SpainUDP to Solve-RD cohort 1 (the unsolved cases; for a description of the Solve-RD cohorts see [www.solve-rd.eu/fact-sheet-solve-rd-cohorts](http://www.solve-rd.eu/fact-sheet-solve-rd-cohorts)).  The dataset includes:  No. of experiments: 5270 (5026 WES / 244 WGS)  No. of individuals: 5127  No. of affected individuals: 2195  No. of families: 2192 |
| **Conditions:** | No additional constraints. |
| **Acknowledgement:** | Contributing groups: Bordeaux, France (Didier Lacombe); Dijon, France (Laurence Faivre); Madrid, Spain (Manuel Posada De la Paz); Manchester, UK (Siddharth Banka); Naples (Vincenzo Nigro); Nijmegen, the Netherlands (Lisenka Vissers); Paris, France (Alain Verloes); Prague, Czech Republic (Milan Macek); Rome, Italy (Marco Tartaglia); Siena, Italy (Alessandra Renieri); Tübingen, Germany (Olaf Riess).  Use the following paragraph to acknowledge the use of this dataset: “*This study makes use of data collated and/or generated by the Solve-RD project, derived from the dataset EGAD00001009770. The Solve-RD project has received funding from the European Union’s Horizon 2020 research and innovation programme under grant agreement No 779257. This study was supported by the European Reference Network ERN-ITHACA (*[*https://ec.europa.eu/health/ern/networks_en)*](https://ec.europa.eu/health/ern/networks_en))*.*”  For group authorship use “Solve-RD DITF-ITHACA”. The most recent version of this group author list including all authors and their affiliations can be requested from the [Solve-RD DAC office](mailto:solve-RD@med.uni-tuebingen.de). |

| **Solve-RD_RND_cohort-1_DF1+2_V1** | |
| --- | --- |
| EGA Study ID: EGAS00001003851 \| EGA dataset ID: EGAD00001009769 | |
| **Brief description:** | This dataset includes genomic (WES and WGS) and phenotypic data as well as pedigree information from unsolved rare disease patients (and their relatives) which have been contributed by ERN-RND partners to Solve-RD cohort 1 (the unsolved cases; for a description of the Solve-RD cohorts see [www.solve-rd.eu/fact-sheet-solve-rd-cohorts](http://www.solve-rd.eu/fact-sheet-solve-rd-cohorts)).  The dataset includes:  No. of experiments: 3174 (2981 WES / 193 WGS)  No. of individuals: 3115  No. of affected individuals: 2697  No. of families: 2511 |
| **Conditions:** | No additional constraints. |
| **Acknowledgement:** | Contributing groups: Antwerp, Belgium (Vincent Timmerman); Barcelona, Spain (Alfons Macaya); Budapest, Hungary (Peter Balisza); Ljubljana, Slovenia (Ales Maver); London, UK (Henry Houlden); Lübeck, Germany (Katja Lohmann); Nijmegen, the Netherlands (Bart van de Warrenburg); Paris, France (Alexis Brice); Tübingen, Germany (Holger Lerche, Olaf Riess, Ludger Schöls, Rebecca Schüle, Matthis Synofzik).  Use the following paragraph to acknowledge the use of this dataset: “*This study makes use of data collated and/or generated by the Solve-RD project, derived from the dataset EGAD00001009769. The Solve-RD project has received funding from the European Union’s Horizon 2020 research and innovation programme under grant agreement No 779257. This study was supported by the European Reference Network ERN-RND (*[*https://ec.europa.eu/health/ern/networks_en)*](https://ec.europa.eu/health/ern/networks_en))*.*”  For group authorship use “Solve-RD DITF-RND”. The most recent version of this group author list including all authors and their affiliations can be requested from the [Solve-RD DAC office](mailto:solve-RD@med.uni-tuebingen.de). |

| **Solve-RD_NMD_cohort-1_DF1+2_V1** | |
| --- | --- |
| EGA Study ID: EGAS00001003851 \| EGA dataset ID: EGAD00001009768 | |
| **Brief description:** | This dataset includes genomic (WES and WGS) and phenotypic data as well as pedigree information from unsolved rare disease patients (and their relatives) which have been contributed by ERN Euro-NMD partners to Solve-RD cohort 1 (the unsolved cases; for a description of the Solve-RD cohorts see [www.solve-rd.eu/fact-sheet-solve-rd-cohorts](http://www.solve-rd.eu/fact-sheet-solve-rd-cohorts)).  The dataset includes:  No. of experiments: 2483 (2342 WES / 141 WGS)  No. of individuals: 2441  No. of affected individuals: 1909  No. of families: 1728 |
| **Conditions:** | No additional constraints. |
| **Acknowledgement:** | Contributing groups: Antwerp, Belgium (Vincent Timmerman); Barcelona, Spain (Alfons Macaya); Barcelona, Spain (Andres Nascimiento Osorio); Cambridge, UK (Rita Horvath); Ferrara, Italy (Alessandra Ferlini); Liverpool, UK (Rajesh Madhu); London, UK (Henry Houlden, Francesco Muntoni); Montpellier, France (Mireille Cossee); Naples, Italy (Vincenzo Nigro); Newcastle, UK (Volker Straub); Ottawa, Canada (Hanns Lochmüller); Oxford, UK (David Beeson); Paris, France (Gisèle Bonne); Tampere, Finland (Bjarne Udd); Tübingen (Olaf Riess).  Use the following paragraph to acknowledge the use of this dataset: “*This study makes use of data collated and/or generated by the Solve-RD project, derived from the dataset EGAD00001009768. The Solve-RD project has received funding from the European Union’s Horizon 2020 research and innovation programme under grant agreement No 779257. This study was supported by the European Reference Network ERN Euro-NMD (*[*https://ec.europa.eu/health/ern/networks_en)*](https://ec.europa.eu/health/ern/networks_en))*.*”  For group authorship use “Solve-RD DITF-EURO-NMD”. The most recent version of this group author list including all authors and their affiliations can be requested from the [Solve-RD DAC office](mailto:solve-RD@med.uni-tuebingen.de). |

| **Solve-RD_GENTURIS_cohort-1_DF1+2_V1** | |
| --- | --- |
| EGA Study ID: EGAS00001003851 \| EGA dataset ID: EGAD00001009767 | |
| **Brief description:** | This dataset includes genomic (WES and WGS) and phenotypic data as well as pedigree information from unsolved rare disease patients (and their relatives) which have been contributed by ERN-GENTURIS partners to Solve-RD cohort 1 (the unsolved cases; for a description of the Solve-RD cohorts see [www.solve-rd.eu/fact-sheet-solve-rd-cohorts](http://www.solve-rd.eu/fact-sheet-solve-rd-cohorts)).  The dataset includes:  No. of experiments: 401 (380 WES / 21 WGS)  No. of individuals: 401  No. of affected individuals: 385  No. of families: 364 |
| **Conditions:** | No additional constraints. |
| **Acknowledgement:** | Contributing groups: Barcelona, Spain (Gabriel Capella); Bonn, Germany (Stefan Aretz); Dresden, Germany (Evelin Schröck); Munich, Germany (Elke Holinski-Feder); Nijmegen, the Netherlands (Richarda de Voer and Nicoline Hoogerbrugge); Porto, Portugal (Carla Oliveira); Tübingen, Germany (Olaf Riess).  Use the following paragraph to acknowledge the use of this dataset: “*This study makes use of data collated and/or generated by the Solve-RD project, derived from the dataset EGAD00001009767. The Solve-RD project has received funding from the European Union’s Horizon 2020 research and innovation programme under grant agreement No 779257. This study was supported by the European Reference Network ERN-GENTURIS (*[*https://ec.europa.eu/health/ern/networks_en)*](https://ec.europa.eu/health/ern/networks_en))*.*”  For group authorship use “Solve-RD DITF-GENTURIS”. The most recent version of this group author list including all authors and their affiliations can be requested from the [Solve-RD DAC office](mailto:solve-RD@med.uni-tuebingen.de). |
