# Supplementary material for: An interconnected data infrastructure to support large-scale rare disease research
Source: Gigascience. 2024 Sep 20;13:giae058. doi: 10.1093/gigascience/giae058 (PMC11413801; doi:10.1093/gigascience/giae058)
Supplement: giae058_Supplemental_Files [file giae058_supplemental_files.zip › Supplementary_information_S3_Data_access_agreement.docx]

**Solve-RD Data Access Agreement**

**This agreement governs the terms on which access will be granted to the sequence and genotype data, “-omics” *Data* (such as transcriptomics, proteomics or metabolomics *Data*) as well as accompanying phenotype *Data* generated by the Solve-RD *Consortium*.**

**For the sake of clarity, the terms of access set out in this agreement apply to all of the *User*, *Authorised Personnel* within the *User’s* research group, and the *User Institution* (as defined below). Within the Agreement “You” and “Your” shall be construed to refer to all these. In signing this agreement, You are agreeing to be bound by the terms and conditions of access set out in this agreement.**

**Title of *Research Project***

120 characters maximum – this will be made public with Your name, institution and project description in lay terms on access being granted

**Name of applicant (*User*), including affiliation and contact details**

| **Name with title:** |  |
| --- | --- |
| **Position:** |  |
| ***User Institution*:** |  |
| **Institutional postal address:** |  |
| **Institutional email address:** |  |
| **Signature:** |  |
| **Date:** |  |

**Name of authorised representative of the *User Institution*,** **including affiliation and contact details**

| **Name with title:** |  |
| --- | --- |
| **Position:** |  |
| **User Institution:** |  |
| **Institutional postal address:** |  |
| **Institutional email address:** |  |
| **Signature:** |  |
| **Date:** |  |

**For the Solve-RD Data Access Committee**

| **Name:** |  |
| --- | --- |
| **Signature:** |  |
| **Date:** |  |

**Datasets for which access is requested**

Please list EGA (European Genome-Phenome Archive) dataset ID(s) (EGAD number).

**Version number of Appendix A that You are including ____**

Appendix A lists any specific conditions attached to individual datasets. You must be using a version that covers all the sets for which You are requesting access.

**Description of proposed *Research Project***

Please provide a clear description of Your *Research Project* and its specific aims in no more than 500 words. This should include specific details of **what You plan to do with the *Data*** and include key references.

If applying to use datasets that have restrictions on the way that they may be used (e.g. must only be used to investigate a specific condition, or may not be used for control purposes), then please clearly state how You plan to use [named datasets] as controls, and that You will respect the [specified] constraints on the use of [named] non-control datasets.

**Short summary in lay terms**

Please provide a short summary of Your project and its aims **in lay terms** using a maximum of 150 words. This will be made public with Your name, institution and project title on access being granted:

***User’s* publication record**

Please list up to 5 relevant publications of which You were an author or co-author, demonstrating Your experience and competence to analyse datasets of this type. If You do not have relevant publications please demonstrate Your expertise and responsibility with respect to human subject’s genetic data analysis.

**List of *Authorised Personnel*** (please refer to definitions section)

| Name with title: |  |
| --- | --- |
| Position: |  |
| Institutional email address: |  |

| Name with title: |  |
| --- | --- |
| Position: |  |
| Institutional email address: |  |

| Name with title: |  |
| --- | --- |
| Position: |  |
| Institutional email address: |  |

(Repeat as necessary)

**Ethics**

*User* and the *User Institution* are aware that some countries/regions do not require ethics approval for use of EGA Data. Depending on the nature of Your research project it is possible, however, that such approval is needed in Your country. If You are uncertain as to whether Your research project needs ethics approval to use EGA Data, we suggest that You contact Your local institutional review board/ research ethics committee (IRB/REC) to clarify the matter.

Please choose from one of the following options:

You represent and warrant that Your country/region does not require Your research project to undergo ethics review.

Your country/region requires Your research project to undergo ethics review, and therefore, this research project has been approved by an IRB/REC formally designated to approve and/or monitor research involving humans.

The approval letter(s) needs to be provided before data access can be given. However, the ethics review process can be started once this data access request has been approved. An institutional approval number should also be provided, if available.

PLEASE NOTE: The EGA and the Solve-RD *Consortium* are not responsible for the ethics approval/monitoring of individual research projects and bear no responsibility for the applicant’s failure to comply with local/national ethical requirements.

**Definitions**

*Authorised Personnel:* Additional individuals who have an affiliation within the research group of the *User* at the *User Institution* including postdocs, students and any visitors. All *Authorised Personnel* must have an email address within the *User Institution*. If multiple research groups within the same institution require access they must each apply. Core IT and other administrative personnel at the *User Institution* who need to have access to the *Data* for data security and management purposes are automatically treated as *Authorised Personnel* and bound by the institutional acceptance of this Agreement**.**

*Collaborator:* A *Collaborator* of the *User*, including both someone working at a different institution from the *User Institution* and someone working in a separate research group in the same *User Institution*.

*Consortium* means the Solve-RD consortium, a list of which can be found as an annex of the Solve-RD Publication Policy on the *Project* website [www.solve-rd.eu](http://www.solve-rd.eu).

*Data:* means all and any human *Data* including but not limited to genomics, metabolomics, lipidomics and phenotype *Data* obtained from the managed access datasets of the *Solve-RD Project*.

*Data Access Committee (DAC):* Access to Solve-RD *Data* will be managed with oversight from the Solve-RD DAC. The constitution of the DAC can be found on the *Project* website [www.solve-rd.eu](http://www.solve-rd.eu).

*Data Producer(s):* The *Solve-RD Project* partners, responsible for the development, organisation, and oversight of the Data.

*Data Subject* means a person, who has been informed of the purpose for which the *Data* is held and has given his/her informed consent thereto.

*Solve-RD Project* or *Project:* Solve-RD is a research project funded by the European Union’s Horizon 2020 research and innovation programme under grant agreement No 779257 from January 2018 to March 2024 (also see *Consortium).*

*Publications:* Includes, without limitation, articles published in print journals, electronic journals, reviews, books, abstracts submitted to conferences, posters and other written and verbal presentations of research.

*Research Project*: shall mean research that is seeking to advance the understanding of genetics and genomics, including the treatment and mechanisms of disorders, and work on statistical methods that may be applied to such research. Further specific conditions apply to particular datasets as listed in Appendix A.

*User:* An applicant having signed this Data Access Agreement, whose *User Institution* has co-signed this Data Access Agreement, both of them having received acknowledgement of its acceptance.

*User Institution:* Institution at which the *User* is employed, affiliated or enrolled. A representative of it has co-signed this Data Access Agreement with the *User* and received acknowledgement of its acceptance.

**Terms and Conditions:**

In signing this agreement, the *User* and the *User Institution*:

1. Agree to only use the *Data* for *Research Projects*, subject to any dataset specific conditions listed in Appendix A, according to the consent obtained from sample donors.
2. Agree to preserve, at all times, the confidentiality of the information and Data. In particular, You undertake not to use, or attempt to use the *Data* to compromise or otherwise infringe the confidentiality of information on *Data Subjects*.
3. Agree to protect the confidentiality of *Data Subjects* in any *Publications* that You prepare by taking all reasonable care to limit the possibility of identification.
4. Agree not to attempt to link or combine the *Data* provided under this Agreement to other information or archived data available for the datasets provided, even if access to that *Data* has been formally granted to You, or it is freely available without restriction, unless specific permission to do so has been received from the relevant access committee(s) or sample custodians.
5. Agree not to transfer or disclose the Data, in whole or part, or any material derived from the *Data* beyond that in *Publications*, to any non-*Authorised Personnel*. Should the *User* or the *User Institution* wish to share the *Data* with a *Collaborator*, the *Collaborator* must complete a separate *Application for Access to the Data*.
6. Agree to use the *Data* for the approved *Research Project* as described in Your *Research Project* description; use of the *Data* for a new purpose or project will require a new application and approval.
7. Accept that *Data* may be reissued from time to time, with suitable versioning. If the reissue is at the request of sample donors and/or other ethical scrutiny, You will replace earlier versions of the *Data* and use the most recent version for subsequent analysis and *Publication*. Notwithstanding, You will be permitted to use the earlier versions of the *Data* to complete Your analysis and to publish it, if necessary. Otherwise, earlier versions of the *Data* are to be destroyed/discarded unless obliged to retain *Data* for archival purposes in conformity with Institutional policy.
8. Agree that in the event of the discovery of a novel gene or of a newly solved case resulting from the analysis of the *Data*, You are required to notify the data submitters without delay (via the Solve-RD DAC office [solve-RD@med.uni-tuebingen.de](mailto:solve-RD@med.uni-tuebingen.de)) In addition, the procedure in section 9 must be taken into account. Information on data submitters and/or group authors is provided in the dataset description (Appendix A) and can be requested from the Solve-RD DAC office.
9. Agree to contact data submitters (via the Solve-RD DAC office [solve-RD@med.uni-tuebingen.de](mailto:solve-RD@med.uni-tuebingen.de)) as soon as possible ahead of *Publication* and invite them to provide input as co-authors. You are strongly encouraged to share key authorship positions with the submitting teams based on the value and amount of *Data* contributed to the *Publication*. Where a *Publication* makes use of *Data* from a large number of submitters of the Solve-RD cohort, a group authorship for Solve-RD should be considered in order to acknowledge the role of all data submitters equally.
10. Agree to acknowledge in any work based in whole or part on the *Data*, the published paper from which the *Data* derives, the version of the *Data*, and the role of the Solve-RD *Consortium* and the relevant primary collectors and their funders.

Suitable wording for such acknowledgement is provided in the dataset description (Appendix A).

1. Agree that the Solve-RD *Consortium*, the original *Data Producers*, Data depositors, copyright holders, and all other parties involved in the creation, funding or protection of any part of the *Data* supplied:
   1. Make no warranty or representation, express or implied as to the accuracy, quality or comprehensiveness of the *Data*;
   2. Exclude to the fullest extent permitted by law all liability for actions, claims, proceedings, demands, losses (including but not limited to loss of profit), costs, awards damages and payments made by You that may arise (whether directly or indirectly) in any way whatsoever from Your use of the *Data* or from the unavailability of, or break in access to, the *Data* for whatever reason and;
   3. Bear no responsibility for the further analysis or interpretation of these *Data*.
2. Understand and acknowledge that the *Data* is protected by copyright and other intellectual property rights, and that duplication, except as reasonably required to carry out Your research with the *Data*, or sale of all or part of the *Data* on any media is not permitted.
3. Recognise that nothing in this Agreement shall operate to transfer to the *User Institution* any intellectual property rights relating to the *Data*.
4. Accept that the *User Institution* has the right to develop intellectual property based on comparisons with their own data, but may not make intellectual property claims on the *Data* nor use intellectual property protection in ways that would prevent or block access to, or use of, any element of the *Data*, or conclusion drawn directly from the *Data*.
5. Agree that You will submit an annual report to the *Data Access Committee*, prior to the one (1) year of the project anniversary, on completion of the agreed *Research Project*. The *Data Access Committee* agrees to treat the report and all information, data, results, and conclusions contained within such report as confidential information belonging to the *User Institution*.
6. Agree that access to the requested dataset(s) is granted for a period of one (1) year, with the option to renew access or close-out a project at the end of that year. *Data* access may be renewed upon certification of a new Data Access Agreement. *Data* access renewal requests will be reviewed for compliance with the terms and conditions of this Data Access Agreement.
7. Agree that *Data* that has been downloaded from the EGA will be permanently deleted/discarded from all local or cloud-based machines when research is completed or it is no longer used for the approved research or this Data Access Agreement is expired, whichever comes first, unless obliged to retain the *Data* for archival purposes in conformity with Institutional policy.
8. Agree that if results arising from the *User* and the *User Institution* use of the *Data* could provide health solutions for the benefit of people in the developing world, the *User* and the *User Institution* agree to offer non-exclusive licenses to such results on a reasonable basis for use in low income and low-middle income countries (as defined by the World Bank) to any party that requests such a license solely for uses within these territories.
9. Agree to update the list of *Authorised Personnel* to reflect any changes or departures in affiliated researchers and personnel within 30 days of the changes made. These changes can be made by emailing [solve-RD@med.uni-tuebingen.de](mailto:solve-RD@med.uni-tuebingen.de).
10. Agree to distribute a copy of this Agreement and explain its content to any person mentioned in the list of *Authorised Personnel*, including any additions made according to paragraph 19.
11. Agree that You will notify the *Data Access Committee* as soon as You become aware of a breach of the terms or conditions of this Agreement, of any unauthorized data sharing, breaches of data security, or inadvertent data releases that may compromise data confidentiality within 24 hours of when the incident is identified. As permitted by law, notifications should include any known information regarding the incident and a general description of the activities or process in place to define and remediate the situation fully. Within three business days of the *Data Access Committee* notification, You agree to submit to the DAC a detailed written report including the date and nature of the event, actions taken or to be taken to remediate the issue(s), and plans or processes developed to prevent further problems, including specific information on timelines anticipated for action.
12. Agree that this agreement expires at the end of the data access period. This agreement may be terminated by either party for any material or persistent breach of the obligations set out in this agreement, by giving thirty (30) days’ written notice to the other of its intention to terminate. The notice shall include a detailed statement describing the nature of the breach. If the breach is capable of being remedied and is remedied within the thirty-day notice period, then the termination shall not take effect. If the breach is incapable of remedy, then the termination shall take effect at the end of the thirty-day notice period in any event. Confidentiality obligations shall continue to apply after termination and continue for five years beginning after the end of the *Solve-RD Project* i.e. from 31 March 2024.
13. Agree that You are responsible for ensuring that all uses of the *Data* are consistent with national and state laws and regulations, as appropriate, as well as relevant institutional policies and procedures for managing sensitive data. All *Data* use shall be in accordance with the Regulation (EU) 2016/679 of the European Parliament and of the Council, of 27 April 2016 (hereinafter, the “GDPR”).
14. Agree that You are responsible for ensuring via technical and organizational measures that only the *User*, the *User Institution* or any *Authorised Personnel* listed in this application document receive access to the datasets.
15. Accept that it may be necessary for the Solve-RD *Consortium* or its appointed agent to alter the terms of this Agreement from time to time. In this event, the Solve-RD *Consortium* or its appointed agent will contact You to inform You of any changes, and You may be required to enter into a new version of the Agreement.
16. If requested, You will allow data security and management documentation to be inspected to verify that You comply with the terms of this Agreement.
17. Agree that the Solve-RD Office may publish the title and project description in lay terms along with the *User's* name and the *User Institution*, as listed in this DAA, on the *Solve-RD Project* website [www.solve-rd.eu](http://www.solve-rd.eu).
18. Agree that amendments to this Data Access Agreement must be in writing and signed by authorized representatives of all parties.

### Understand that this Agreement (and any dispute, controversy, proceedings or claim of whatever nature arising out of this Agreement or its formation) shall be construed, interpreted and governed by the laws of Belgium and shall be subject to the exclusive jurisdiction of the Belgian courts.

1. Agree that during the *Solve-RD Project* and for a period of one (1) year after the project i.e. until 31 March 2025 every paper that is published and includes *Data* produced and/or collated within Solve‐RD has to be confirmed by the Solve‐RD *Consortium*.

You will inform the Solve-RD *Consortium* (via the Solve-RD office [solve-RD@med.uni-tuebingen.de](mailto:solve-RD@med.uni-tuebingen.de)) of any planned *Publication* at least 45 calendar days before the intended date of submission. Any objection to the planned *Publication* following the above notification shall be made in writing within thirty (30) calendar days after receipt of the notice. If no objection is made within the time limit stated above, the *Publication* is permitted.

The objection has to include a precise and reasonable request for necessary modifications, it being specified that any such modifications shall not harm the scientific content of the proposed *Publication*.

**WHEN SUBMITTING THIS DOCUMENT, PLEASE INCLUDE ALL PAGES OF THE AGREEMENT WITH THE SIGNATURE PAGE**

**Appendix A: Data Set Specific Conditions**

**Version 1**
