# Supplementary figures and images for: An interconnected data infrastructure to support large-scale rare disease research
Source: Gigascience. 2024 Sep 20;13:giae058. doi: 10.1093/gigascience/giae058 (PMC11413801; doi:10.1093/gigascience/giae058)

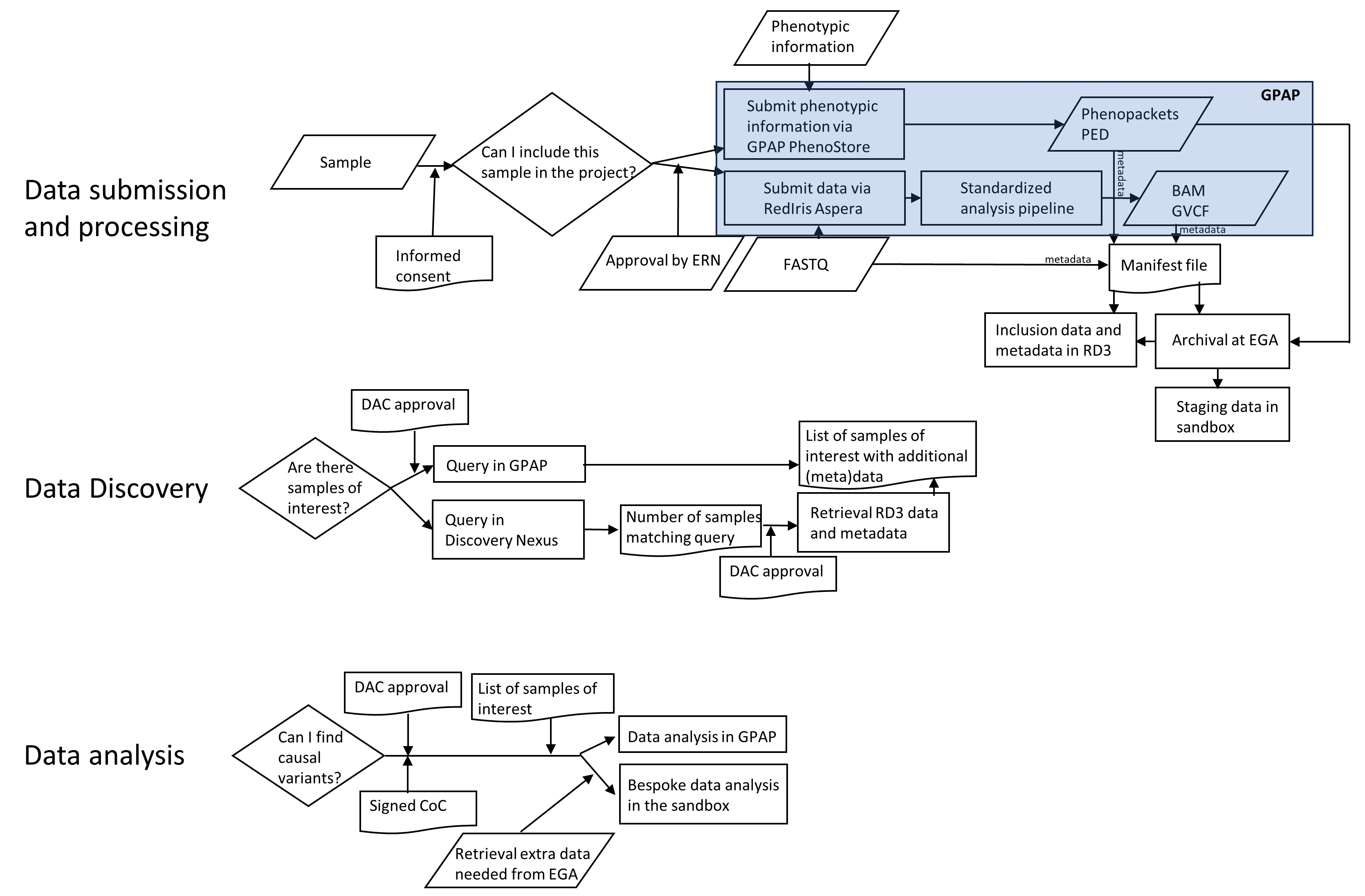

Supplement: giae058_Supplemental_Files [file giae058_supplemental_files.zip › Figure_S1_Supplementary Material.png]
