# Supplementary material for: An interconnected data infrastructure to support large-scale rare disease research
Source: Gigascience. 2024 Sep 20;13:giae058. doi: 10.1093/gigascience/giae058 (PMC11413801; doi:10.1093/gigascience/giae058)
Supplement: giae058_Response_to_Reviewer_Comments_Original_Submission [file giae058_response_to_reviewer_comments_original_submission.pdf]

Author's Response To Reviewer Comments

Reviewer #1: The manuscript describes Solve-RD, an infrastructure gathers a set of reusable tools and best practices for multi-omics analyses in the context of Rare Diseases. The work was realized within the context of four European Reference Networks and two associated ERNs and demonstrates long-standing efforts made during several months.
The manuscript is long and complete, however the choice on how to divide topics and present the architecture results into a quite cryptic explanation. In invite the authors to make further efforts to make the manuscript more understandable for the readership, especially since the goal is that of establishing 'a blueprint for other projects that need to analyse large amounts of heterogeneous data'.

We thank the reviewer for this comment. We have chosen this set-up to focus in the results on the infrastructure as a whole, its setup and the flow of the data. In the methods we describe the separate components and their details. We understand that too many details specific to the current set-up were present in the results. We have now removed those to make it more general, and give more pointers. Even though the focus on rare disease analysis is still prominent in the results, we hope it will now be easier to follow for researchers in different fields and as such also be more useful as a blueprint for data analysis infrastructure.

A few specific comments follow:

1) I am not convinced with the use of the word "proven" (referring to the infrastructure design) as this would require a user validation study or some kind of evaluation against similar infrastructures.

We have replaced the word proven in the abstract with 'succesfully deployed and useful'.

2) In the introduction, I read "Furthermore, to enable researchers from different centers to work together on the same dataset a cloud infrastructure is needed accessible by all researchers." After reading this, I was expecting a specific discussion on how the project dealt with data integration and data FAIRification challenges. These are mentioned sporadically (e.g., when speaking about "appropriate metadata" that is collected), but not systematically. I think a proper FAIR principles assessment of the infrastructure would give the manuscript an interesting section.

We agree with the reviewer that the FAIRness of the infrastructure is an important message of the paper. In the discussion we have a short section on this. We have made a few adjustments to make clearer how the infrastructure and processes connect to the FAIR components. In addition, we added a box in the results section to explicitly name the FAIR parts of the infrastructure.

3) The description of the two external services RD3 and Discovery Nexus and how they interact with the other sandboxes could be made more explicit

We have now written in the results: "Via RD3 and the advanced discovery layer 'Discovery Nexus', DATF bioinformaticians can find samples and data of interest. To do this they formulate queries that identify file identifiers (EGAF) for relevant data stored in EGA, to then access these data in the sandbox, in GPAP, or in their local cluster." This should make the relation between RD3, the EGA file identifiers and the analysis clusters/GPAP more clear.

4) I have some concerns about figures. In a quite long manuscript such as this one, it is essential that they are readable, understandable and capable of summarize contributions.
- Fig 1 should guide the reader when reading the phases described in the results. However, I find the correspondence quite hard. As an example, there is no mention of the GPAP PhenoStore or of the RedIris

Aspera server in the image. The image should be aligned to the text or vice versa. This seems more an internal sketch rather than a proper workflow for a publication. Mixing input/output formats, used techniques or tools, without a proper semantics in the arrows among blocks.

We thank the reviewer for pointing out the need to understand figure 1 in combination with the text of the paper. We acknowledge that the figure is conveying various messages, making it difficult to understand. We have made changes to the figure to better align it to the text and add the missing pieces and aligning it to the paper sections.
• In response to a comment of reviewer 2: Changed WES and WGS to ES and GS, respectively
• Add '(RedIris)' to the 'Aspera' key information.
• We have now stated in the figure that RD-Connect GPAP includes PhenoStore and the standardized analysis pipeline
• We have added an arrow between GPAP and RD3 to indicate the connection between the components.
• We have added Beacon Network to RD-Connect GPAP and Discovery Nexus.
• We have added the black arrow to the list of keys to indicate that it represents the data flow.
We agree that figure 1 does not show a workflow. It is meant as an overview of the infrastructure and its connections, because multiple workflows are possible using the same infrastructure. To accommodate this we have added a supplementary figure (S1) showing archetypical workflow that can be performed on the infrastructure.

- Authors mention that "The many data types and relationships in Solve-RD are summarised in Figure 3." I would define this as a data model of the Solve-RD package" employed to organize information within the project.

We have now rewritten the sentence to "The data model of the Solve-RD project describing how data is organized is summarised in Figure 3."

- Figure 4 and 5a/b are hardly readable. This may be due to some loss of quality during the submission process but I invite authors to check or to provide figures separately as Suppl. Materials.

Unfortunately, the resolution of the figures was indeed too low for figure 5. We have submitted these in 300dpi. Figure 4 was already submitted at 300 dpi, so indeed the rendering of the pdf may have lowered the quality. We are unsure if the original submitted images are available for the reviewers next to the pdf. We will ask the editor if this is possible.

Minor: Check the sentence at line 298 - 200 page 14. It does not read well.

We thank the reviewer for noticing this sentence.
We have now changed it to "Using this infrastructure, the Solve-RD project has already made >500 new diagnoses, and many analyses powered by novel omics data are still ongoing."

Reviewer #2: This paper is about the architecture of the data and analytics infrastructure that supports the Solve-RD project for improving the diagnostic rate in rare diseases.
The Solve-RD project is a large multinational consortium of 51 institutes from 15 (European) countries, and is impressive in its scale and ambition.

Comments

The paper does not explicitly state its intended audience, however I think a statement of the intended audience would be beneficial. Based on the material presented and the level of technical detail, it seems mostly aimed at an audience that is familiar with modern genomics technologies and it interested in how

the infrastructure is built from a fairly high-level perspective.
We agree with the author that parts of the manuscript, especially the methods, are quite detailed. As stated in the last sentence of the background section: “We believe the resulting infrastructure could provide a template which future large scale RD analysis projects can start from.” we intended as the audience consortia wishing to perform a similar project in which large amounts of data provided by many service providers are jointly analyzed. Parts of the template can also be used for projects with other topics than rare disease, however most tools and models are created specifically for rare disease research. We have added the sentence “Most of the components are tailored for RD research, however, the general design and some components of the infrastructure can also be of use for groups focusing on other topics.” to state this more explicitly.

The paper does not include scientific outcomes from the project, which is fine, however, it would be useful to have a brief overview of the outcomes to date, such as how many previously unsolved rare disease cases have been solved by the project.

The main scientific outcomes of the project are stated in a separate paper (Laurie, S. et al. Genomic Reanalysis of a Pan-European Rare Disease Resource Yields >500 New Diagnoses) which is currently under submission. In the first section of the discussion, we refer to this paper stating that already >500 new diagnoses have been made. We feel that going into more detail would not be adding to the goal of our paper.

There are several key parts to the infrastructure, and upon first reading it was difficult to keep all of the parts clear in my mind, especially because there are also a lot of associated acronyms to remember. I had to re-read the paper a few times to properly understand the parts. Ideally Figure 1 should have been more helpful in tracking the purpose of all the parts. However, I found Figure 1 to be rather unhelpful upon first reading. Perhaps it is because Figure 1 has a lot of text in it, and there is no obvious order in which to read the parts of the diagram. Additionally, it is not entirely clear upon first viewing what the arrows are supposed to mean (yes, it is some kind of data flow), but the kind of data flow is unclear. Perhaps Figure 1 could be simplified by reducing the amount of text and replacing the text with some very high-level descriptions of the roles that each part plays in the infrastructure?

We acknowledge that figure 1 is not easy to understand because it represents the entire infrastructure including data flow. We have tried to describe the role of each of the components in the italics text in the top boxes of each component. In addition, as explained in the answer to question 4 of reviewer 1, we have added missing components to the figure to better align it with the main text. We appreciate that the acronyms are difficult to understand, however, apart from RD-Connect GPAP, EGA and RD3 these are laboratory techniques and file types that are common within the field of genetics. Providing full names would in our opinion make the figure more difficult to understand. We have now added the black arrow to the key section to describe that it refers to a data flow. We have not included the type of data flowing from one component to the next. However, the normal text in the bottom box of the components describes which data is available for the component.

The title of the paper says "unified" but some aspects of the system appear to be more distributed. For example, the system relies on the EGA for some parts of data access management. Some aspects of the data analysis rely on somewhat external HPC resources. All of these are fine, and make sense from a design point of view, but it doesn't match my expectation of what "unified" means. Perhaps the authors could provide more discussion about what makes the system unified?

We agree with the reviewer that the term ‘unified’ suggests that the parts of the infrastructure are packaged together more closely than they are. We have now replaced ‘unified’ with ‘interconnected’.

It can be difficult to review complex platforms such as this without the ability to log in and test it out as a user. However, I realise that this can be very challenging for maintaining security and privacy and integrity of peer review. Figures 4,5a,5b attempt to remedy this somewhat by providing screenshots of the user

interface. Unfortunately they were hard to read for me due to being low resolution. But they also don't really convey the workflow that a user would typically follow. I think it would be useful to have a figure that showed the most typical user workflows, perhaps as a flow chart (screen shots not necessary). e.g. The user logs in through W. They browse the data through X. They find data they are interested in and submit a data request through Y. If granted access they analyse the data through Z.

As mentioned in the reaction to the comments of reviewer 1, unfortunately during the automatic creation of the pdf upon submission the quality of the high-resolution images has been reduced. Hopefully you are able to access the original pictures. We will ask the editor if this is the case.

Regarding the second remark, the infrastructure supports many different possible workflows. However, overall they can be boiled down to three different workflows matching the subheaders in the results and methods section. We have added these workflows as supplementary Figure 1 to which we refer in the discussion. In addition, we have added a short section in the Discussion to look at the infrastructure from different user perspectives.

Related to the above point, it is not clear to me what is the name of the overall platform. Is it the "RD-Connect Genome-Phenome Analysis Platform (GPAP)"? I would normally expect the name to be in the title of the paper or at least mentioned at the very beginning, ie "In this paper we describe XXX, ..." Having a clearly defined name for the whole system would also make it easier to discuss.

The infrastructure itself does not have a specific name, other than ‘the Solve-RD project infrastructure’. We have now referred to the infrastructure as such in the last section of the background. Note that GPAP is one of the components of the project infrastructure, although it can be considered as an infrastructure itself.

We have decided to keep the title more general, because the paper is meant to provide a template to create similar infrastructures and not to ‘sell’ the infrastructure as a whole.

It is very encouraging to see that the platform is built primarily from existing components and standards where possible. It would be useful to have a table that summarises all the components that are used to build the system. This table could also include the version numbers, license information and link to the code repository. It could also include any novel software that was created specifically for the project.

We thank the reviewer for this suggestion. We have included this table (Table 1) at the beginning of the methods section.

I did notice that some of the components used in the system have not been updated for a while. These include MOLGENIS and MME. Looking at the MOLGENIS github suggests that it has been superseded by MOLGENIS-EMX2. What does this mean for others who might be thinking of adopting the infrastructure described here? Would it be necessary to migrate over to MOLGENIS-EMX2?

The reviewer is correct that MOLGENIS has been updated to the EMX2 version during the project. Currently an EMX2 version of RD3 is being created in the context of a different project (https://demo-recon41md.molgeniscloud.org/demo/tables/#/). A new project may choose to use the current model of RD3, however we would suggest using the latest available version. We have now included the sentence “As Solve-RD partners continue to use the infrastructure to perform many multi-omics analyses, the operational support teams are actively synergising with related projects, ensuring sustainability and further development of the different infrastructure components” in the discussion to make clear to the reader that many of the components are under active development.

MME is a tool that is used within some of the components to provide the possibility to match Solve-RD cases to cases outside of the project to aid Solving those cases. MME is not developed or maintained by any of the groups involved in this project. It is true that the model behind MME has been stable for a long

time, however the MME Participants are active nodes, meaning that new cases are regularly available for matching.

The authors state that "We believe the resulting infrastructure could provide a template which future large scale RD analysis projects can start from", which I think is true, but also why limit the scope to RD analysis? A lot of projects beyond RD could probably benefit from parts of the platform.

As indicated in our answer to your first comment we agree that also other projects could benefit from this template, although the components we are using here are tailored towards RD analysis. We have added the extra sentence: “Most of the components are tailored for RD research, however, the general design and some components of the infrastructure can also be of use for groups focusing on other topics.” to indicate that the template is also usable to design infrastructure for research on other topics.

It is not clear to me if/how external users can request data access through the system? Does the platform have a DAC management component? Something like REMS or custom built?

Because of the primary focus on the infrastructure we had initially omitted the information on data access. However, we understand that this may indeed be of interest. We have now added an extra paragraph to the discussion explaining the set-up of the data access procedure. This procedure is outside the infrastructure described in the paper.

“The Solve-RD omics data (i.e. pre-existing unsolved exomes and genomes as well as omics data generated within the project) are archived in the European Genome-phenome Archive (EGA) and will be made available to other rare disease researchers via a controlled access mechanism, governed by the Solve-RD Data Access Committee (DAC). The DAC consists of one representative per ERN that contributed data and/or samples to Solve-RD as well as a patient representative. Researchers who would like to access a specific Solve-RD dataset need to request access from the Solve-RD DAC. To do this they have to fill and sign the Solve-RD Data Access Agreement (DAA), which is available at https://solve-rd.eu/results/solve-rd-data/, and send it to the DAC office. The DAA lays out the terms under which access to Solve-RD data (including sequence and genotype data, other omics data, phenotypic data and pedigree information) is being granted.”

The discussion becomes a bit repetitive of the points made in the results section. Perhaps this could be made more concise? Likewise in the methods section. It seems to repeat a lot of the material from the previous sections, with more elaboration. The paper would benefit from being less repetitive.

Regarding the methods section. We have deliberately written a relatively extensive methods section with the same main sections as the results to explain the technical background of the infrastructure to enable bioinformaticians to create a similar structure. The results section is aimed at the users of the infrastructure. Because the general flow is identical it can indeed read as being repetitive. We have critically looked at the different sections to remove unnecessary parts of the text (especially in the results section). Together with the adaptations made to figure 1 this should make the text easier to follow.

There is a brief section in the discussion about the future of the project, although I found this too brief. It would be nice to know what are the plans for the platform and what its lifespan is likely to be. Oftentimes papers are written about platforms near the end of their lifespan, and readers can be disappointed to find out that the system is no longer supported. So it would be useful to have some indication of where this is headed.

The reviewer is correct that the Solve-RD project will soon end. The components used to create the infrastructure are sustained via different projects. We have added extra information on this at the end of the discussion. However, the main goal of this paper is to provide a blueprint for other projects to build their own infrastructure using these or similar components. The complete infrastructure as used throughout the project will come to an end, because after the end of the project analyses will not be done as part of the project, but outside. Therefore, for instance, the funding is too limited to have a large sandbox suitable

for analyses of high volumes of data. However, parts of the infrastructure and the data will remain available, so that the data is findable and accessible for researchers interested in the data analyzed within the Solve-RD project.

Minor comments

The paper uses ES and GS for exome and genome sequencing. In my experience the literature has tended to use WES and WGS for these terms. Perhaps ES and GS reflect a more modern nomenclature. If the authors consider ES and GS to be correct modern nomenclature then that is fine. However, if these terms are new to this paper then I suggest reverting to WES and WGS to follow the previous convention. I also note that Figure one uses the WES an WGS nomenclature instead of ES and GS.

We thank the reviewer for noticing the discrepancy in naming between figure 1 and the rest of the paper. We have chosen to use the terms ES and GS rather than WES and WGS to refer to exome and genome sequencing to remain consistent between this paper and the associated paper describing the project results (Laurie et al., in revision). The reason behind this is that not the entire exome or genome is covered because of limitations in capturing kit or sequencing procedure, and therefore is may still be the case that variants are not detected even though they reside in the exome or genome.
We have adapted figure 1 to now also state ES and GS.

(page 17) "the data is automatically imbibed". I've not seen the word "imbibed" used in this context before. More commonly I've seen "ingested". Imbibed seems like it might be suitable for the task, but perhaps ingested might be more standard?

We have replaced “imbibed” with “ingested”.

To the editor.
We have read, discussed and answered the questions of the two reviewers and adapted the manuscript accordingly. One of the comments was on the resolution of figures 4, 5a and 5b. We have now uploaded new images 5a and 5b with a higher resolution, although the quality of the images may also be lessened by the rendering into the pdf. Is it possible for the reviewers to download the original images as submitted?
We have added a table in the manuscript listing the different tools and if applicable their registration.
