# Supplementary material for: An interconnected data infrastructure to support large-scale rare disease research
Source: Gigascience. 2024 Sep 20;13:giae058. doi: 10.1093/gigascience/giae058 (PMC11413801; doi:10.1093/gigascience/giae058)
Supplement: giae058_Response_to_Reviewer_Comments_Revision_1 [file giae058_response_to_reviewer_comments_revision_1.pdf]

Author's Response To Reviewer Comments

We thank the editor and both reviewers for the work put in to the review and the further comments. We believe the manuscript has benefitted a lot from the useful comments.

Reviewer reports:
Reviewer #1: I appreciate the addition of Supplement Figure S1 which helps clarifying the different steps of the workflow. The higher quality figures allowed me to assess their information. All my other comments have been addressed.

Thank you.

Reviewer #2: Thank you to the authors for careful consideration of the comments made in the previous review.

I think the paper has benefitted substantially from the changes that have been made.

Overall I'm happy with the paper and only have some minor suggestions below.

I still think that the paper is probably a bit too long, so it would benefit from some further editing to make it a bit more concise, however, I will leave that to the authors and editors to decide on the appropriate length.

Here are my minor comments. I don't think another round of reviewing is needed if these items can be considered by the authors:

We have corrected the typo's

page 10 "Consortium Code Of Conduct" previously this was called "project code of conduct" maybe standardise on the name?

We now have also called it the project Code of Conduct on page 10.

page 11 "has also lit a beacon" I understand that "lighting a beacon" is the terminology used within the Beacon community, but I find it to be less helpful for those outside the community. Many readers will not know that this is special jargon in the community and it will be confusing. By all means, use the jargon, but it would be useful to have its meaning also spelled out for non-specialists.
We have changed "lit a beacon" to "created a beacon"

page 16 "the operational support teams are actively synergising with related projects" I'm not really sure what this means specifically, maybe it could be stated more clearly?
We have changed "synergising" to "working together"

page 17 "pseudonymised" perhaps you can elaborate on specifically what was done to pseudo-anonymise the data?
The pseudonimization process here means that only the submitter knows the full identifiers of a participant submitted to RD-Connect GPAP. Essentially the pseudonimization entails the assignment of a P-ID to each participant.
We have now added the sentence "The relation between P-ID and full personal identifiers is known only to the submitter." To the section on p17/18.
