# Supplementary material for: An interconnected data infrastructure to support large-scale rare disease research
Source: Gigascience. 2024 Sep 20;13:giae058. doi: 10.1093/gigascience/giae058 (PMC11413801; doi:10.1093/gigascience/giae058)
Supplement: giae058_Reviewer_1_Report_Original_Submission — Anna Bernasconi, Ph.D. -- 1/6/2024 Reviewed [file giae058_reviewer_1_report_original_submission.pdf]

Reviewer Report

Title: An interconnected data infrastructure to support large-scale rare disease research

Version: Original Submission **Date:** 1/6/2024

Reviewer name: Anna Bernasconi, Ph.D.

Reviewer Comments to Author:

The manuscript describes Solve-RD, an infrastructure gathers a set of reusable tools and best practices for multi-omics analyses in the context of Rare Diseases. The work was realized within the context of four European Reference Networks and two associated ERNs and demonstrates long-standing efforts made during several months.
The manuscript is long and complete, however the choice on how to divide topics and present the architecture results into a quite cryptic explanation. In invite the authors to make further efforts to make the manuscript more understandable for the readership, especially since the goal is that of establishing 'a blueprint for other projects that need to analyse large amounts of heterogeneous data'.
A few specific comments follow:
1) I am not convinced with the use of the word "proven" (referring to the infrastructure design) as this would require a user validation study or some kind of evaluation against similar infrastructures.
2) In the introduction, I read "Furthermore, to enable researchers from different centers to work together on the same dataset a cloud infrastructure is needed accessible by all researchers." After reading this, I was expecting a specific discussion on how the project dealt with data integration and data FAIRification challenges. These are mentioned sporadically (e.g., when speaking about "appropriate metadata" that is collected), but not systematically. I think a proper FAIR principles assessment of the infrastructure would give the manuscript an interesting section.
3) The description of the two external services RD3 and Discovery Nexus and how they interact with the other sandboxes could be made more explicit
4) I have some concerns about figures. In a quite long manuscript such as this one, it is essential that they are readable, understandable and capable of summarize contributions.
- Fig 1 should guide the reader when reading the phases described in the results. However, I find the correspondence quite hard. As an example, there is no mention of the GPAP PhenoStore or of the RedIris Aspera server in the image. The image should be aligned to the text or viceversa. This seems more an internal sketch rather than a proper workflow for a publication. Mixing input/output formats, used techniques or tools, without a proper semantics in the arrows among blocks.
- Authors mention that "The many data types and relationships in Solve-RD are summarised in Figure 3." I would define this as a data model of the Solve-RD package" employed to organize information within the project.
- Figure 4 and 5a/b are hardly readable. This may be due to some loss of quality during the submission process but I invite authors to check or to provide figures separately as Suppl. Materials.
Minor: Check the sentence at line 298 - 200 page 14. It does not read well.

Methods

Are the methods appropriate to the aims of the study, are they well described, and are necessary controls included? Choose an item.

Conclusions

Are the conclusions adequately supported by the data shown? Choose an item.

Reporting Standards

Does the manuscript adhere to the journal's guidelines on minimum standards of reporting? Choose an item.

Choose an item.

Statistics

Are you able to assess all statistics in the manuscript, including the appropriateness of statistical tests used? Choose an item.

Quality of Written English

Please indicate the quality of language in the manuscript: Choose an item.

Declaration of Competing Interests

Please complete a declaration of competing interests, considering the following questions:

- Have you in the past five years received reimbursements, fees, funding, or salary from an organisation that may in any way gain or lose financially from the publication of this manuscript, either now or in the future?
- Do you hold any stocks or shares in an organisation that may in any way gain or lose financially from the publication of this manuscript, either now or in the future?
- Do you hold or are you currently applying for any patents relating to the content of the manuscript?
- Have you received reimbursements, fees, funding, or salary from an organization that holds or has applied for patents relating to the content of the manuscript?
- Do you have any other financial competing interests?
- Do you have any non-financial competing interests in relation to this paper?

If you can answer no to all of the above, write 'I declare that I have no competing interests' below. If your reply is yes to any, please give details below.

I declare that I have no competing interests

I agree to the open peer review policy of the journal. I understand that my name will be included on my report to the authors and, if the manuscript is accepted for publication, my named report including any attachments I upload will be posted on the website along with the authors' responses. I agree for my report to be made available under an Open Access Creative Commons CC-BY license (http://creativecommons.org/licenses/by/4.0/). I understand that any comments which I do not wish to be included in my named report can be included as confidential comments to the editors, which will not be published.

Choose an item.

To further support our reviewers, we have joined with Publons, where you can gain additional credit to further highlight your hard work (see: https://publons.com/journal/530/gigascience). On publication of this paper, your review will be automatically added to Publons, you can then choose whether or not to claim your Publons credit. I understand this statement.

Yes Choose an item.
