# Supplementary material for: An interconnected data infrastructure to support large-scale rare disease research
Source: Gigascience. 2024 Sep 20;13:giae058. doi: 10.1093/gigascience/giae058 (PMC11413801; doi:10.1093/gigascience/giae058)
Supplement: giae058_Reviewer_2_Report_Original_Submission — Bernie Pope, Ph.D. -- 1/15/2024 Reviewed [file giae058_reviewer_2_report_original_submission.pdf]

Reviewer Report

Title: An interconnected data infrastructure to support large-scale rare disease research

Version: Original Submission **Date:** 1/15/2024

Reviewer name: Bernie Pope, Ph.D.

Reviewer Comments to Author:

This paper is about the architecture of the data and analytics infrastructure that supports the Solve-RD project for improving the diagnostic rate in rare diseases.
The Solve-RD project is a large multinational consortium of 51 institutes from 15 (European) countries, and is impressive in its scale and ambition.
Comments

The paper does not explicitly state its intended audience, however I think a statement of the intended audience would be beneficial. Based on the material presented and the level of technical detail, it seems mostly aimed at an audience that is familiar with modern genomics technologies and it interested in how the infrastructure is built from a fairly high-level perspective.
The paper does not include scientific outcomes from the project, which is fine, however, it would be useful to have a brief overview of the outcomes to date, such as how many previously unsolved rare disease cases have been solved by the project.
There are several key parts to the infrastructure, and upon first reading it was difficult to keep all of the parts clear in my mind, especially because there are also a lot of associated acronyms to remember. I had to re-read the paper a few times to properly understand the parts. Ideally Figure 1 should have been more helpful in tracking the purpose of all the parts. However, I found Figure 1 to be rather unhelpful upon first reading. Perhaps it is because Figure 1 has a lot of text in it, and there is no obvious order in which to read the parts of the diagram. Additionally, it is not entirely clear upon first viewing what the arrows are supposed to mean (yes, it is some kind of data flow), but the kind of data flow is unclear. Perhaps Figure 1 could be simplified by reducing the amount of text and replacing the text with some very high-level descriptions of the roles that each part plays in the infrastructure?
The title of the paper says "unified" but some aspects of the system appear to be more distributed. For example, the system relies on the EGA for some parts of data access management. Some aspects of the data analysis rely on somewhat external HPC resources. All of these are fine, and make sense from a design point of view, but it doesn't match my expectation of what "unified" means. Perhaps the authors could provide more discussion about what makes the system unified?
It can be difficult to review complex platforms such as this without the ability to log in and test it out as a user. However, I realise that this can be very challenging for maintaining security and privacy and integrity of peer review. Figures 4,5a,5b attempt to remedy this somewhat by providing screenshots of the user interface. Unfortunately they were hard to read for me due to being low resolution. But they also don't really convey the workflow that a user would typically follow. I think it would be useful to have a figure that showed the most typical user workflows, perhaps as a flow chart (screen shots not

necessary). e.g. The user logs in through W. They browse the data through X. They find data they are interested in and submit a data request through Y. If granted access they analyse the data through Z.
Related to the above point, it is not clear to me what is the name of the overall platform. Is it the "RD-Connect Genome-Phenome Analysis Platform (GPAP)"? I would normally expect the name to be in the title of the paper or at least mentioned at the very beginning, ie "In this paper we describe XXX, ..." Having a clearly defined name for the whole system would also make it easier to discuss.
It is very encouraging to see that the platform is built primarily from existing components and standards where possible. It would be useful to have a table that summarises all the components that are used to build the system. This table could also include the version numbers, license information and link to the code repository. It could also include any novel software that was created specifically for the project.
I did notice that some of the components used in the system have not been updated for a while. These include MOLGENIS and MME. Looking at the MOLGENIS github suggests that it has been superseded by MOLGENIS-EMX2. What does this mean for others who might be thinking of adopting the infrastructure described here? Would it be necessary to migrate over to MOLGENIS-EMX2?
The authors state that "We believe the resulting infrastructure could provide a template which future large scale RD analysis projects can start from", which I think is true, but also why limit the scope to RD analysis? A lot of projects beyond RD could probably benefit from parts of the platform.
It is not clear to me if/how external users can request data access through the system? Does the platform have a DAC management component? Something like REMS or custom built?
The discussion becomes a bit repetitive of the points made in the results section. Perhaps this could be made more concise? Likewise in the methods section. It seems to repeat a lot of the material from the previous sections, with more elaboration. The paper would benefit from being less repetitive.
There is a brief section in the discussion about the future of the project, although I found this too brief. It would be nice to know what are the plans for the platform and what its lifespan is likely to be. Oftentimes papers are written about platforms near the end of their lifespan, and readers can be disappointed to find out that the system is no longer supported. So it would be useful to have some indication of where this is headed.
Minor comments

The paper uses ES and GS for exome and genome sequencing. In my experience the literature has tended to use WES and WGS for these terms. Perhaps ES and GS reflect a more modern nomenclature. If the authors consider ES and GS to be correct modern nomenclature then that is fine. However, if these terms are new to this paper then I suggest reverting to WES and WGS to follow the previous convention. I also note that Figure one uses the WES an WGS nomenclature instead of ES and GS.
(page 17) "the data is automatically imbibed". I've not seen the word "imbibed" used in this context before. More commonly I've seen "ingested". Imbibed seems like it might be suitable for the task, but perhaps ingested might be more standard?

Methods

Are the methods appropriate to the aims of the study, are they well described, and are necessary controls included? Choose an item.

Conclusions

Are the conclusions adequately supported by the data shown? Choose an item.

Reporting Standards

Does the manuscript adhere to the journal's guidelines on minimum standards of reporting? Choose an item.

Choose an item.

Statistics

Are you able to assess all statistics in the manuscript, including the appropriateness of statistical tests used? Choose an item.

Quality of Written English

Please indicate the quality of language in the manuscript: Choose an item.

Declaration of Competing Interests

Please complete a declaration of competing interests, considering the following questions:

- Have you in the past five years received reimbursements, fees, funding, or salary from an organisation that may in any way gain or lose financially from the publication of this manuscript, either now or in the future?
- Do you hold any stocks or shares in an organisation that may in any way gain or lose financially from the publication of this manuscript, either now or in the future?
- Do you hold or are you currently applying for any patents relating to the content of the manuscript?
- Have you received reimbursements, fees, funding, or salary from an organization that holds or has applied for patents relating to the content of the manuscript?
- Do you have any other financial competing interests?
- Do you have any non-financial competing interests in relation to this paper?

If you can answer no to all of the above, write 'I declare that I have no competing interests' below. If your reply is yes to any, please give details below.

I declare that I have no competing interests

I agree to the open peer review policy of the journal. I understand that my name will be included on my report to the authors and, if the manuscript is accepted for publication, my named report including any attachments I upload will be posted on the website along with the authors' responses. I agree for my

report to be made available under an Open Access Creative Commons CC-BY license (http://creativecommons.org/licenses/by/4.0/). I understand that any comments which I do not wish to be included in my named report can be included as confidential comments to the editors, which will not be published.

Choose an item.

To further support our reviewers, we have joined with Publons, where you can gain additional credit to further highlight your hard work (see: https://publons.com/journal/530/gigascience). On publication of this paper, your review will be automatically added to Publons, you can then choose whether or not to claim your Publons credit. I understand this statement.

Yes Choose an item.
