# Supplementary material for: An interconnected data infrastructure to support large-scale rare disease research
Source: Gigascience. 2024 Sep 20;13:giae058. doi: 10.1093/gigascience/giae058 (PMC11413801; doi:10.1093/gigascience/giae058)
Supplement: giae058_Reviewer_2_Report_Revision_1 — Bernie Pope, Ph.D. -- 5/9/2024 Reviewed [file giae058_reviewer_2_report_revision_1.pdf]

Reviewer Report

Title: An interconnected data infrastructure to support large-scale rare disease research

Version: Revision 1 **Date:** 5/9/2024

Reviewer name: Bernie Pope, Ph.D.

Reviewer Comments to Author:

Thank you to the authors for careful consideration of the comments made in the previous review.
I think the paper has benefitted substantially from the changes that have been made.
Overall I'm happy with the paper and only have some minor suggestions below.
I still think that the paper is probably a bit too long, so it would benefit from some further editing to make it a bit more concise, however, I will leave that to the authors and editors to decide on the appropriate length.
Here are my minor comments. I don't think another round of reviewing is needed if these items can be considered by the authors:
page 5 "requiredfor"
page 6 ".." two full stops
page 6 "a secondary dools" -> "a secondary tool"?
page 7 ". ." two full stops
page 10 "Consortium Code Of Conduct" previously this was called "project code of conduct" maybe standardise on the name?
page 11 ". ." two full stops
page 11 "has also lit a beacon" I understand that "lighting a beacon" is the terminology used within the Beacon community, but I find it to be less helpful for those outside the community. Many readers will not know that this is special jargon in the community and it will be confusing. By all means, use the jargon, but it would be useful to have its meaning also spelled out for non-specialists.
page 16 "the operational support teams are actively synergising with related projects" I'm not really sure what this means specifically, maybe it could be stated more clearly?
page 17 "pseudonymised" perhaps you can elaborate on specifically what was done to pseudo-anonymise the data?

Methods

Are the methods appropriate to the aims of the study, are they well described, and are necessary controls included? Choose an item.

Conclusions

Are the conclusions adequately supported by the data shown? Choose an item.

Reporting Standards

Does the manuscript adhere to the journal's guidelines on [minimum standards of reporting?] Choose an item.

Choose an item.

Statistics

Are you able to assess all statistics in the manuscript, including the appropriateness of statistical tests used? Choose an item.

Quality of Written English

Please indicate the quality of language in the manuscript: Choose an item.

Declaration of Competing Interests

Please complete a declaration of competing interests, considering the following questions:

- Have you in the past five years received reimbursements, fees, funding, or salary from an organisation that may in any way gain or lose financially from the publication of this manuscript, either now or in the future?
- Do you hold any stocks or shares in an organisation that may in any way gain or lose financially from the publication of this manuscript, either now or in the future?
- Do you hold or are you currently applying for any patents relating to the content of the manuscript?
- Have you received reimbursements, fees, funding, or salary from an organization that holds or has applied for patents relating to the content of the manuscript?
- Do you have any other financial competing interests?
- Do you have any non-financial competing interests in relation to this paper?

If you can answer no to all of the above, write 'I declare that I have no competing interests' below. If your reply is yes to any, please give details below.

I declare that I have no competing interests

I agree to the open peer review policy of the journal. I understand that my name will be included on my report to the authors and, if the manuscript is accepted for publication, my named report including any attachments I upload will be posted on the website along with the authors' responses. I agree for my report to be made available under an Open Access Creative Commons CC-BY license (http://creativecommons.org/licenses/by/4.0/). I understand that any comments which I do not wish to be included in my named report can be included as confidential comments to the editors, which will not be published.

Choose an item.

To further support our reviewers, we have joined with Publons, where you can gain additional credit to further highlight your hard work (see: https://publons.com/journal/530/gigascience). On publication of this paper, your review will be automatically added to Publons, you can then choose whether or not to claim your Publons credit. I understand this statement.

Yes Choose an item.
